# Integrated chemical profiling and lipidomics reveal that Mailuoning oral liquid alleviates diet-induced MASLD via PPARα-mediated lipid homeostasis and COX-2 inhibition, with identification of potential active constituents

**DOI:** 10.3389/fphar.2026.1939197

**Published:** 2026-10-29

**Authors:** Sijia Wu, Wangya Jia, Xun Zhou, Linyan Zhu, Keke Wang, Jian Li, Lili Ji, Wei Liu, Hong Xu

**Affiliations:** 1 Institute of Chinese Materia Medica, Shanghai University of Traditional Chinese Medicine, The MOE Key Laboratory for Standardization of Chinese Medicines, Shanghai, China; 2 Department of Pharmacy, The SATCM Third Grade Laboratory of Traditional Chinese Medicine Preparations, Key Laboratory of Liver and Kidney Diseases (Ministry of Education), Shanghai Key Laboratory of Traditional Chinese Clinical Medicine, Shuguang Hospital Affiliated to Shanghai University of Traditional Chinese Medicine, Shanghai, China; 3 Institute of Chinese Medicinal Materials, Gansu Academy of Agricultural Sciences, Lanzhou, Gansu, China; 4 Department of Pharmaceutics, China Pharmaceutical University, Nanjing, Jiangsu, China; 5 Technology Center of Jinling Pharmaceutical Co., Ltd., Nanjing, Jiangsu, China

**Keywords:** bioactive constituents, COX-2, lipidomics, mailuoning oral liquid, MASLD, PPARα

## Abstract

**Background:**

Mailuoning oral liquid (MLN), a standardized five-herb formula, is used for ischemic vascular diseases and shows potential in ameliorating dyslipidemia. Our previous study showed MLN alleviates metabolic dysfunction-associated steatotic liver disease (MASLD) via peroxisome proliferator-activated receptor α (PPARα) activation, but its comprehensive chemical profile, systemic exposure, and bioactive constituents mediating both PPARα activation and cyclooxygenase-2 (COX-2) inhibition remain unclear.

**Methods:**

High-fat, high-fructose diet-fed rats received MLN (5.4 or 10.8 mL/kg/day) for 8 weeks. Ultra-high-performance liquid chromatography coupled with Q-Exactive Orbitrap high-resolution mass spectrometry (UHPLC-Q-Exactive Orbitrap HRMS) profiled MLN chemicals and identified serum/liver prototypes. Biochemical profiles, glucose tolerance, histopathology, untargeted lipidomics, and targeted polyunsaturated fatty acid (PUFA) analysis were performed. PPARα and COX-2 signaling were examined by quantitative real-time polymerase chain reaction (qPCR), western blotting, and immunohistochemistry. Key constituents were screened for PPARα activation via dual luciferase reporter assay and molecular docking; COX-2 inhibition was validated by enzyme assays and molecular dynamics.

**Results:**

A total of 143 compounds were characterized; 48 and 92 prototypes were enriched in serum and liver, respectively. MLN ameliorated dyslipidemia, glucose intolerance, steatosis, and inflammation. Lipidomics revealed bidirectional modulation of hepatic lipids, reversing triglyceride (TAG) accumulation and restoring phosphatidylcholine (PC) and lysophosphatidylcholine (LPC) levels. Targeted analysis showed increased eicosapentaenoic acid (EPA) and normalized COX-2-derived prostaglandins (PGD_2_, PGE_2_). MLN activated hepatic PPARα and downregulated COX-2. Seven serum-enriched compounds (chlorogenic acid, macranthoidin B, luteoloside, harpagide, angoroside C, oleanolic acid, betaine) were validated as direct PPARα agonists. Secologanoside and chikusetsusaponin IVa were identified as novel COX-2 inhibitors (half-maximal inhibitory concentration (IC_50_) = 14.61 and 120.9 nM).

**Discussion:**

MLN alleviates MASLD via dual PPARα activation and COX-2 inhibition, providing a mechanistic foundation for repurposing this herbal formula in early-stage MASLD.

## Graphical Abstract

**Figure:**
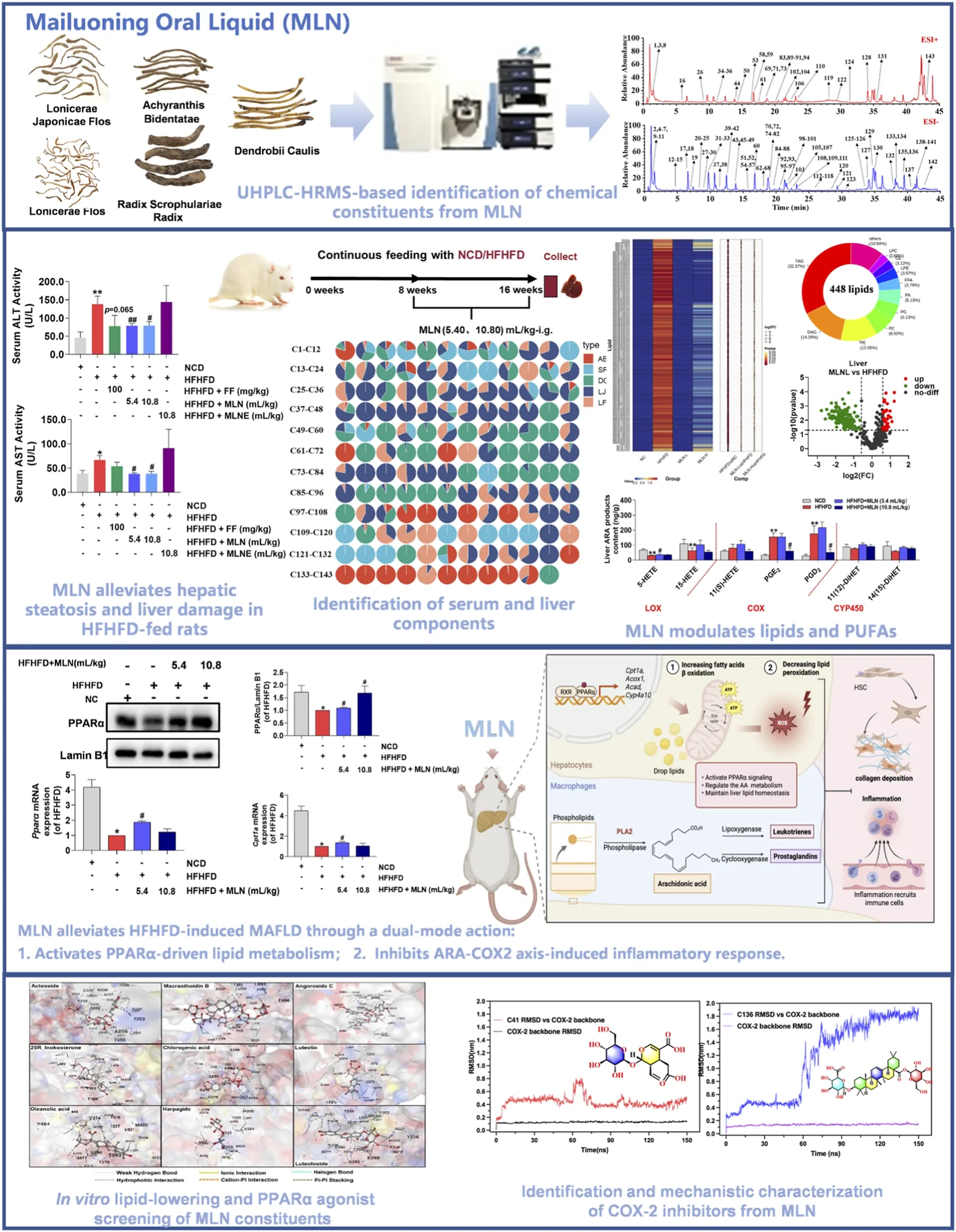

## 1 Introduction

Metabolic dysfunction associated steatotic liver disease (MASLD) is now recognized as a systemic disorder in which hepatic steatosis coexists with extrahepatic metabolic dysfunction, and may progress from simple steatosis to metabolic-dysfunction-associated-steatohepatitis (MASH), fibrosis, cirrhosis and even hepatocellular carcinoma (Han et al., 2023; Santos-Sánchez and Cruz-Chamorro, 2025). Over only 3 decades, the global prevalence of MASLD has risen from 25.3% (1990–2006) to 38.0% (2016–2019) (Wong et al., 2023). Despite this alarming trajectory, therapeutic options remain woefully inadequate: only one drug, Rezdiffra™ (resmetirom), has been approved for MASH with moderate-to-severe fibrosis (liver biopsy F2–F3, according to the NASH Clinical Research Network [NASH CRN] fibrosis stage), leaving the vast majority of patients with earlier disease stages without any licensed pharmacotherapy (Harrison et al., 2023). Consequently, there exists an urgent unmet medical need for safe, preventive interventions capable of restoring hepatic lipid homeostasis and interrupting inflammatory signaling prior to the onset of irreversible liver damage.

Dysregulation of hepatic lipid homeostasis is the earliest pathogenic event in MASLD. Excessive triglycerides (TAGs), diacylglycerols, ceramides and other bioactive lipids provoke lipotoxicity, oxidative stress, insulin resistance and chronic inflammation (Luukkonen et al., 2016). In contrast, glycerophospholipids, particularly phosphatidylcholine (PC) and phosphatidylethanolamine (PE), maintain membrane integrity and very-low-density lipoprotein secretion (Peng et al., 2021), while polyunsaturated fatty acids (PUFAs) such as arachidonic acid (ARA, 20:4 Ω-6) and eicosapentaenoic acid (EPA, 20:5 Ω-3) serve as precursors of pro- or anti-inflammatory eicosanoids, respectively (Djuricic and Calder, 2021). Over-expression of cyclooxygenase-2 (COX-2) drives ARA toward prostaglandin D_2_ and E_2_ (PGD_2_, PGE_2_), amplifying hepatic inflammation (Tilg and Moschen, 2010); selective COX-2 inhibitors are effective but carry cardiovascular caveats (Nissen, 2012), prompting the search for safer, naturally derived alternatives.

Peroxisome proliferator-activated receptor α (PPARα) is a master nuclear receptor that senses free fatty acids and their metabolites, orchestrating hepatic lipid turnover. Upon ligand binding, PPARα translocates to the nucleus, heterodimerizes with retinoid X receptor and trans-activates genes bearing peroxisome proliferator response element (PPRE) motifs, including carnitine palmitoyltransferase 1A (Cpt1a), acyl-CoA oxidase 1, lysophosphatidylcholine acyltransferase 3 and fibroblast growth factor 21, thereby accelerating mitochondrial and peroxisomal *β*-oxidation, promoting phospholipid remodeling, and suppressing lipogenic transcription factors such as sterol regulatory element-binding protein-1c (Bougarne et al., 2018). PPARα also cross-talks with nuclear factor-kappa B to downregulate COX-2 expression and reduce the generation of pro-inflammatory eicosanoids (Staels et al., 2013). Consistently, PPARα-null mice develop spontaneous hepatic steatosis and exhibit elevated ceramides and DAG species, whereas pharmacological PPARα agonists, such as fenofibrate (FF), restore PC/PE balance, increase EPA-containing phospholipids and decrease COX-2-derived prostaglandins in dietary models of MASLD (Francque et al., 2015). These findings establish PPARα as an attractive molecular hub that couples lipid homeostasis to inflammatory control. Despite the established efficacy of synthetic fibrates, their clinical use is constrained by muscle toxicity, unfavorable drug-drug interactions, and poor long-term patient adherence (Staels et al., 1998). These limitations have catalyzed interest in identifying natural products capable of selectively activating PPARα or inhibiting COX-2 without adverse systemic effects, particularly for early-stage MASLD where preventive strategies hold the greatest promise (Villarroel-Vicente et al., 2021).

Mailuoning oral liquid (MLN) is a modern formulation derived from the classic traditional Chinese medicine (TCM) prescription Simiao Yong’an Decoction, originally recorded in *Yan Fang Xin Bian* and comprising Lonicerae Japonicae Flos (LJF), Scrophulariae Radix (SR), Angelicae Sinensis Radix, and Glycyrrhizae Radix et Rhizoma (Zhao et al., 2020). MLN was developed by removing Angelicae Sinensis Radix and Glycyrrhizae Radix et Rhizoma, and adding Achyranthis Bidentatae Radix (ABR), Dendrobii Caulis (DC) and Lonicerae Flos (LF) to enhance blood-activating and yin-nourishing effects, yielding the current five-herb composition (State Food and Drug Administration, 2002). This optimized combination resulted in the current five-herb composition of MLN, which retains the core principle of “clearing heat and detoxifying” from Simiao Yong’an Decoction while expanding its therapeutic scope to include modern vascular and metabolic indications.

In its present form, MLN is a standardized, officially approved five-herb formula composed of ABR, SR, DC, LJF, and LF in a fixed ratio of 25:25:25:16:9 (Standard No.: WS_3_-B-3462–98-2011; State Food and Drug Administration, 2002). It has been clinically used in China for over 3 decades for ischemic cerebrovascular and peripheral vascular diseases. A Cochrane systematic review of 21 randomized controlled trials (*n* = 1,746) concluded that MLN is safe and improves neurological outcomes after acute ischemic stroke, establishing a substantial clinical safety profile (Yang et al., 2015). Notably, emerging clinical evidence suggests that MLN possesses significant lipid-lowering effects and improves metabolic parameters in patients with type 2 diabetes and hyperlipidemia (Lin and Li, 2010; Liu et al., 2000), providing a compelling rationale for its repurposing in metabolic disorders.

Capitalizing on this clinical potential, our group has systematically investigated the metabolic pharmacology of MLN. We first demonstrated that MLN alleviates methionine-choline-deficient (MCD) diet-induced MASLD. Through an integrated network pharmacology and *in vivo* validation approach, we found that this hepatoprotective effect was associated with the upregulation of hepatic peroxisome proliferator-activated receptor γ coactivator-1α, increased nuclear accumulation of PPARα protein, and the downregulation of key lipogenic genes, collectively identifying PPARα as a central functional target (Jia et al., 2022). A subsequent mechanistic study confirmed that MLN attenuates diet-induced obesity specifically by activating the PPARα/fibroblast growth factor 21 axis, which promotes adipose tissue thermogenesis (Jia et al., 2025). These findings establish that modulation of the PPARα pathway as a pivotal mechanism underlying the metabolic benefits of MLN. Parallel advances in phytochemical analysis have begun to map MLN’s complex chemical landscape. Recent studies have identified or tentatively characterized 60 major compounds in MLN, spanning phenolic acids, flavonoids, iridoids, and saponins, while establishing simultaneous quantification methods for 40 bioactive constituents, including markers such as neochlorogenic acid, loganic acid, chlorogenic acid, and isoacteoside, thereby providing a robust chemical and quality control framework (Tan et al., 2023).

To address these questions, we designed an integrated multi-omics and systems pharmacology study. First, we performed ultra-high-performance liquid chromatography coupled with Q-Exactive Orbitrap high-resolution mass spectrometry (UHPLC-Q-Exactive Orbitrap HRMS) to profile MLN and identify the prototype constituents that reach the systemic circulation and hepatic tissue. Using a high-fat, high-fructose diet (HFHFD)-induced rat model of early MASLD, we then integrated untargeted lipidomics with targeted PUFA metabolic flux analysis to test the hypothesis that MLN restores lipid homeostasis via PPARα-driven reprogramming while simultaneously suppressing the PUFA-COX-2 inflammatory axis. Finally, we employed *in vitro* enzyme/cell assays and molecular dynamics simulations to pinpoint the specific bioavailable molecules responsible for PPARα activation and COX-2 inhibition. This work provides comprehensive, lipid-centric mechanistic evidence to support the repurposing of MLN as a multi-target natural therapeutic for MASLD.

## 2 Materials and methods

### 2.1 Chemicals and reagents

Reference standards (>98% purity) were purchased from the following suppliers. Alfa Biotechnology Co., Ltd. (Chengdu, China) provided ajugol (Lot: AF21062703), secoxyloganin (Lot: AF21070609), secologanoside (Lot:AFDL1351), dipsacoside B (Lot: AF21121404), sweroside (Lot: AF21092003), geniposide (Lot: AF21020852), acteoside (Lot: AF20021101), cynaroside (Lot: AF20072618), aucubin (Lot: AF21061352), lonicerin (Lot: AF22031401), angoroside C (Lot: AF21052502), isochlorogenic acid C (Lot: AF20121801), macranthoside B (Lot: AF2113009), macranthoidin A (Lot: AF22021113), macranthoidin B (Lot: AF21081706), 1,3-Dicaffeoylquinic acid (Lot: AF21110203), salicylic acid (Lot: AF21052253), chikusetsusaponin iva (Lot: AF21050701), (+)-syringaresinol-β-D-glucoside (Lot: AFBG2704), loganin (Lot: AZ220411101), (−)-syringaresinol (Lot: AFBH2406), aurantiamide acetate (Lot: AFBH3011), apigenin-7-O-*D*-glucoside (Lot: AFBL0605) and (−)-syringaresinol-4-O-*β*-D-glucopyranoside (Lot: AFBH2407). Preferred Biological Technology Co., Ltd. (Chengdu, Sichuan, China) supplied betaine (Lot: 19101701), 3,4-dihydroxybenzoic acid (Lot: 20052804), quercetin (Lot: 21032401), cryptochlorogenic acid (Lot: 21061801), schaftoside (Lot: 1906401), chlorogenic acid (Lot: 21032502), neochlorogenic acid (Lot: 20123006), vicenin 3 (Lot: 17022005), rubrosterone (Lot: 21090603), gallic acid (Lot: 21012903), harpagoside (Lot: 21070503), cinnamic acid (Lot: 21032206), isochlorogenic acid B (Lot: 21012902), isorhamnetin (Lot: 20120204), caffeic acid (Lot: 20110501), amentoflavone (Lot: 19071101), luteolin (Lot: 20121604), 4-hydroxy-benzaldehyde (Lot: 21102903), moupinamide (Lot: 22022503), (+)-lyoniresinol 9′-O-glucoside (Lot: 22070602), rutin (Lot: 21010804), ferulic acid (Lot: 21011503) and sucrose (Lot: 20122806). Loganic acid (Lot: BP0883) was obtained from Biopurify Phytochemicals Ltd. (Chengdu, China). Meilun Biotechnology Co., Ltd. (Dalian, China) delivered 20-Hydroxyecdysone (Lot: MB6934), hesperidin (Lot: A1006AS), and harpagide (Lot: MB6660). Zhenzhun Co. (Shanghai, China) furnished (±)14,15- Epoxyeicosatrienoic acid (EET, Lot: 23Y141-C8), (±)11,12-EET (Lot: 23Y141-N4), (±)5-hydroxyeicosatetraenoic acid (HETE, Lot: 23Y140-D4), (±)15-HETE (Lot: 23Y140-G5), 11(S)-HETE (Lot: 23Y086-D3), PGE_2_ (Lot: 23Z296-J1), PGD_2_ (Lot: 23Z293-D1), (±)8,9-EET (DiHET, Lot:23Y141-F7), (±)14(15)- dihydroxyeicosatrienoic acid (DiHET, Lot), (±)11(12)-DiHET (Lot: 23Y141-F7), and ARA (Lot: 23Y052-P1). (25R)-Inokosterone (Lot: O20GB163868), (25S)-Inokosterone (Lot: O14GB163870), oleanolic acid (Lot: K15S5C1), DHA (docosahexaenoic acid, Lot: D25HS204727), and eicosapentaenoic acid (EPA, Lot: T90225) were obtained from Yuanye Biotechnology Co., Ltd. (Shanghai, China); Isoschaftoside (Lot: 18022831) was sourced from Tauto Biotech Co., Ltd. (Shanghai, China) and gastrodin (Lot: 151208) from Victory Biotechnology Co., Ltd. (Chengdu, China).

Assay kits for total cholesterol (TC, Lot: A111-1–1), non-esterified fatty acid (NEFA, Lot: A042-2–1), triglyceride (TG, Lot: A110-1–1), and alanine and aspartate aminotransferase (ALT, Lot: C009-1–1 and AST, Lot: C010-1–1) were purchased from Jiancheng Bioengineering Institute (Nanjing, China). A prostaglandin E_2_ (PGE_2_) ELISA kit (Lot: BY-WJZF1266) was obtained from Nanjing Byabscience Technology Co., Ltd. (Nanjing, China). The rat insulin ELISA kit was obtained from Millipore Corporation (Billerica, USA, Lot: RAB0904). Accu-Chek Performa test strips and Performa Connect were supplied by Roche (Basel, Switzerland). Normal chow diet (NCD, Lot: TP2834060C) and HFHFD (Lot: TP2834060) were provided by Trophic Animal Feed High-Tech Co., Ltd., (Nantong, China); FF came from Hengshan Pharmaceutical Co. (Shanghai, China). High performance liquid chromatography (HPLC) -grade formic acid and methanol were purchased from Fisher Scientific (USA). Antibodies against PPARα and LaminB1 were obtained from Santa Cruz Biotechnology (Dallas, USA, Lot:sc-398394) and Abways Biotechnology (Shanghai, China) respectively. SYBR green and reverse transcriptase kits were provided by Yisheng Biopharma (Shanghai, China, Lot: 11201ES08,11300ES93).

LF (Lot: Z084-2303–01), LJF (Lot: Z037-2303–04), SR (Lot: Z038-2303–04), ABR (Lot: Z039-2303–05), and DC (Lot: Z040-2212–30) were provided and authenticated by the Technology Center of Jinling Pharmaceutical Co., Ltd. (Nanjing, China). DC complied with the import drug registration standard issued by the State Food and Drug Administration (SFDA, No. JZ20160001), while the other four crude drugs met the quality specifications stipulated in the Chinese Pharmacopoeia Commission (2025) edition.

MLN, MLN excipients (MLNE), and the water extracts of ABR, SR, DC, LJF, and LF were prepared by Jinling Pharmaceutical Co., Ltd. (Nanjing, China). The water extracts of ABR, SR, DC, LJF, and LF were prepared using the identical manufacturing process as the MLN oral solution. The MLNE vehicle was also manufactured using the identical preparation process as the MLN oral solution, with the sole difference being the omission of the MLN herbal extract. The MLNE preparation contained exclusively the excipient components—Tween-80 (polysorbate 80, as a solubilizer) and stevioside (as a sweetener)—and was devoid of any constituents derived from the MLN herbal material. The quality of MLN was verified by HPLC quantification of harpagide, chlorogenic acid, *β*-ecdysterone, cymaroside, isochlorogenic acid A, harpagoside, macranthoidin B, and dipsacoside B at 0.160, 2.406, 0.222, 0.037, 0.327, 0.073, 4.317, and 0.090 mg/mL, respectively, according to a previously reported method (Jia et al., 2022). Representative HPLC chromatograms are provided in Supplementary Figure S1.

### 2.2 Development of UHPLC-Q-Exactive Orbitrap HRMS methodology

A UHPLC-Q-Exactive Orbitrap HRMS analytical method was developed using a Dionex Ultimate 3000 UHPLC system coupled with a Q-Exactive Orbitrap high-resolution mass spectrometer equipped with an electrospray ionization (ESI) source, to characterize the chemical constituents of MLN.

#### 2.2.1 Preparation of standard solutions

Each standard was accurately weighed, dissolved in methanol, and prepared as a stock solution with an approximate concentration of 1 mg/mL. Subsequently, a specific volume of each standard stock solution was diluted with 20% methanol to achieve a concentration of 40 μg/mL, thereby creating individual reference standard solutions. In addition, each individual solution was diluted with 20% methanol according to the calculated volume to prepare multiple mixed standard working solutions at a concentration of 2 μg/mL. All prepared solutions were stored at −20 °C for subsequent experimental use.

#### 2.2.2 Sample preparation

An appropriate volume of MLN was diluted with 20% methanol to a concentration of 200 mg/mL, based on the amount of raw medicinal materials, to obtain the MLN test solution. Aqueous extracts of ABR, SR, DC, LJF and LF, prepared according to the manufacturing process of MLN, were each taken in appropriate amounts and diluted with 20% methanol to a concentration of 100 mg/mL, based on the amount of raw medicinal materials, to prepare individual test solutions. All test solutions were filtered through 0.22 μm membrane filters.

#### 2.2.3 Chromatography and mass spectrometry conditions

Separation was achieved with an ACQUITY UPLC™ BEH C_18_ column (100 mm × 2.1 mm, 1.7 μm) maintained at 40 °C. The mobile phase comprised 0.1% formic acid in water (A) and methanol (B). Gradient elution occurred over 45 min at a flow rate of 0.3 mL/min, with an injection volume of 2 μL. Detailed elution conditions and mass spectrometry parameters are shown in Supplementary Tables S1 and S2.

Data acquisition and processing, including full-scan MS and MS^2^ data, were conducted using the Thermo Xcalibur 2.2 workstation. Peaks with intensities exceeding 10,000 were selected for subsequent identification to maximize the acquisition of ESI (electrospray ionization)-MS/MS fragment ions. The chemical formulas of deprotonated and protonated molecular ions were derived from precise mass measurements and possible elemental compositions, under the following restrictions: C [0–70], H [0–120], O [0–40], N [0–6], S [0–2], Na [0–2]. The mass deviation between the measured and calculated values was ensured to be <10 ppm.

### 2.3 HFHFD-MASLD model establishment and UHPLC-Q-Exactive Orbitrap HRMS analysis of MLN exposure in serum and liver

#### 2.3.1 Animals and experimental design

Thirty-six male Sprague-Dawley rats (200 ± 20 g) were obtained from the Shanghai Experimental Laboratory Animal Center of Chinese Academy of Science (Shanghai, China) (certificate number: SCXK (Hu) 2022–0004), and were housed at Shanghai University of Traditional Chinese Medicine (Laboratory Animal License Number: SYXK (Hu) 2020–0009) under a standard laboratory diet and tap water daily. Rat have free access to food and water and have received humane care according to the institutional animal care guidelines approved by the Experimental Animal Ethical Committee of Shanghai University of Traditional Chinese Medicine (Approved number: PZSHUTCM210226009).

Six groups were set up: (1) NCD group (*n* = 6), (2) HFHFD group (*n* = 6), (3) HFHFD + FF (100 mg/kg) group (*n* = 6), (4) HFHFD + MLN (5.4 mL/kg) group (*n* = 6), (5) HFHFD + MLN (10.8 mL/kg) group (*n* = 6), and (6) HFHFD + MLNE (10.8 mL/kg) group (*n* = 6). The rats were fed an HFHFD continuously for 16 weeks, while the NCD group was maintained on a low-fat control diet throughout the experiment. After 8 weeks of diet-induced modeling, the rats in the treatment group were administered orally with MLN, MLNE, or FF for an additional 8 weeks. During this period, we recorded the body weight and food intake of each rat, and serum and liver tissues were collected for biochemical analysis.

#### 2.3.2 Experimental protocol for evaluating MLN efficacy in HFHFD-MASLD rats

Glucose tolerance test: Rat ***s*** were fasted for 4 hours without access to water, and blood samples were collected from the tail vein at 30, 60, 90, and 120 min post-administration of an oral glucose solution (2 g/kg) to measure blood glucose levels.

Serum and liver biochemical analysis: Using appropriate assay kits, we determined the serum activities of ALT and AST, the serum levels of insulin, and the levels of TC and TG in the liver.

Histological observation of the liver: Fixed liver tissue was dehydrated, cleared, and embedded in paraffin, then sectioned and stained with Oil Red O. For immunohistochemistry, rat liver sections underwent antigen-repair and were then blocked with 5% bovine serum albumin (BSA), and incubated overnight with F4/80 antibody at 4 °C. Sections were treated with secondary antibodies at room temperature and developed with 3,3′-diaminobenzidine (DAB). Sections were then examined and photographed under a light microscope.

Real-time polymerase chain reaction (RT-PCR) and western blotting: RT-PCR and western blotting protocols were described in our previous study (Jia et al., 2022). Primers are given in Supplementary Table S3.

#### 2.3.3 Analysis of MLN components entering the serum and liver by UHPLC-Q-Exactive Orbitrap HRMS

Three serum samples from the MLN group (10.8 mL/kg) collected in Section 2.3.1 were randomly selected and mixed in equal amounts to obtain 100 μL of mixed serum samples for the treatment group. Next, we added 500 μL of methanol, vortexed for 30 s and centrifuged at 12,700 rpm for 10 min at 4 °C. The supernatant (480 μL) was transferred to a clean tube and dried at 37 °C under nitrogen. The residue was reconstituted with 80 μL of 20% methanol, vortexed for 30 s, and the supernatant was retained for analysis. Similarly, we took each sample of rat liver corresponding to the serum from Section 2.3.1 and mixed to obtain 100 mg; then, we added 1000 μL of methanol and steel beads. After homogenization for 2 min and centrifugation for 10 min, the supernatant (880 μL) was blown dry at 37 °C under nitrogen. The residue was reconstituted with 160 μL of 20% methanol and vortexed and centrifuged for 30 s. Next, 100 μL supernatant was collected and analyzed using the UHPLC-Q-Exactive Orbitrap HRMS method described in Section 2.2.3. Serum and liver samples from the HFHFD group were processed in an identical manner.

### 2.4 Non-targeted lipidomics analysis of serum and liver using the AB Sciex Triple Quad™ 4500

Untargeted lipidomic profiling of rat serum and liver tissue was executed with an AB Sciex Triple Quad™ 4500 (Zheng et al., 2020).

#### 2.4.1 Sample preparation for serum and liver tissue

For sample preparation, 20 μL of rat serum or liver homogenate (the liver was homogenized with saline at a 1:2 ratio) was combined with 700 μL of ice-cold isopropanol. The mixture was thoroughly combined, vortexed for 1 min, and then incubated at ambient temperature for 10 min prior to overnight storage at −20 °C. The following day, the sample was subjected to centrifugation at 12,700 rpm for 20 min at 4 °C. Then, 600 μL of the supernatant was carefully transferred and subjected to a second round of centrifugation under identical parameters to obtain the final supernatant for analysis. In addition, 20 μL aliquots from each sample were combined to formulate a quality control (QC) sample.

#### 2.4.2 Chromatography and mass spectrometry conditions

Chromatographic separation was executed on a Shimadzu LC-30AD system fitted with a Kinetex C_18_ column (100 mm × 2.1 mm, 2.6 μm; Phenomenex), which was maintained at 40 °C and operated at a flow rate of 0.3 mL/min. Mobile phase A was methanol-acetonitrile-water (1:1:1, v/v/v) with 5 mM ammonium acetate, and Mobile phase B was isopropanol. Supplementary Table S4 presents the detailed gradient elution conditions. The AB Sciex Triple Quad™ 4500 instrument was used to carry out multiple reaction monitoring. The atomizing gas (gas 1, N2), dry gas (gas 2, N2), and curtain gas were set to 60, 60, and 35 psi, respectively. In negative ionization mode, the ion spray voltage and declustering potential (DP) were set to −4500 V and −80 V, respectively. In positive ionization mode, the ISV and DP were adjusted to 5500 V and 60 V, respectively. Supplementary Table S5 shows mass spectral parameters for each compound in both modes.

### 2.5 Profiling of Ω-3 and Ω-6 PUFAs in serum and liver using the AB Sciex Triple Quad™ 4500

The detection of Ω-3 and Ω-6 PUFAs was performed using a method previously established by our research group on an LC-30AD tandem AB Sciex Triple Quad™ 4500 instrument (Zhang et al., 2025).

#### 2.5.1 Preparation of control solution

A stock solution (100 μg/mL) was diluted with the mixed solvent (H_2_O: methanol: acetonitrile, 2:1:1, v/v/v) to a concentration of 5 μg/mL. Serial dilutions were then performed to generate working solutions with concentrations of 1000, 400, 160, 64, 25.6, 10.24, 4.10, and 1.64 ng/mL.

#### 2.5.2 Serum and liver sample preparation

A 50 μL aliquot of serum was added to 250 μL of mixed solvent (methanol: acetonitrile, 1:1, *v*/*v*), vortex-mixed for 30 s, and centrifuged at 12,700 rpm at 4 °C for 10 min. The supernatant (240 μL) was collected, concentrated by vacuum drying at 35 °C, and reconstituted in 80 μL of mixed solvent (H_2_O: methanol: acetonitrile, 2:1:1, *v*/*v*/*v*). For liver tissue, a precise 50 mg portion was weighed, and 1000 μL of mixed solvent (methanol: acetonitrile, 1:1, *v*/*v*) and two steel beads were added. The mixture was homogenized at 65 Hz for two 1-min cycles, then centrifuged at 12,700 rpm at 4 °C for 10 min. The supernatant (800 μL) was collected, concentrated by vacuum drying at 35 °C, and reconstituted in 160 μL of mixed solvent (H_2_O: methanol: acetonitrile, 2:1:1, *v*/*v*/*v*). Both serum and liver samples were centrifuged under the same conditions after reconstitution, and the supernatant was collected for analysis.

#### 2.5.3 Chromatography and mass spectrometry conditions

Chromatographic separation was performed on an ACQUITY UPLC™ BEH column (100 mm × 2.1 mm, i.d. 1.7 μm) at 36 °C with a flow rate of 0.25 mL/min. Mobile phases A and B were 0.1% aqueous formic acid and acetonitrile, respectively. Negative ionization mode was used, and the ion spray voltage was set at −4500 V. The gradient elution procedure and the multiple reaction monitoring conditions of the PUFAs are shown in Supplementary Table S6.

### 2.6 Screening and validation of PPARα agonist activity

#### 2.6.1 BODIPY fluorescence staining

Intracellular lipid accumulation was assessed using a fluorescent fatty acid analog. HepaRG cells were seeded in 24-well plates at a density of 1 × 10^5^ cells per well. After attachment, the medium was replaced with RPMI-1640 containing 1% BSA. Cells were divided into the following treatment groups: (1) 1% BSA control; (2) NEFA model (0.5 mM NEFA, oleic acid:palmitic acid = 2:1); (3) NEFA + low-dose compound; (4) NEFA + high-dose compound. Test compounds were added to the drug groups, while equal volumes of DMSO were added to the control and model groups. All groups were treated for 24 h. After treatment, the medium was removed, cells were washed with PBS, fixed with 4% paraformaldehyde for 30 min, and then stained with BODIPY FL C16 (1 µM) and DAPI (1 μg/mL) for 15 min in the dark. Following three washes with PBS, images were acquired using a fluorescence microscope (IX81, Olympus, Japan), and intracellular lipid content was quantified based on relative fluorescence intensity.

#### 2.6.2 Molecular docking virtual screening

Three-dimensional structures of active compounds were obtained from the PubChem database and converted to pdbqt format. The crystal structure of the human PPARα ligand-binding domain (PDB ID: 3ET1) was retrieved from the Protein Data Bank (PDB). Protein preprocessing, including removal of water molecules and addition of hydrogen atoms, was performed using PyMOL. Semi-flexible molecular docking was conducted using AutoDock Vina, with PPARα as the receptor and each active compound as the ligand. Default parameters were used for docking calculations, and binding affinity was expressed as computed binding energy (ΔG, kcal/mol). The best-docked conformations were visualized and analyzed using PyMOL.

#### 2.6.3 PPRE-luciferase reporter gene assay

HEK-293T cells were seeded in 96-well plates at a density of 1.5 × 10^4^ cells per well. After overnight culture, cells were co-transfected with a PPRE-driven firefly luciferase reporter plasmid and a Renillaluciferase internal control plasmid (pRL-TK) using Lipofectamine 3000 according to the manufacturer’s instructions. Six hours post-transfection, the medium was replaced with complete culture medium. After an additional 24 h of incubation, the medium was replaced with fresh medium containing test compounds or vehicle control (DMSO). Following another 24 h of incubation, the medium was removed, and cells were lysed with Passive Lysis Buffer. The lysate was centrifuged at 12,000 × g for 5 min, and 20 µL of the supernatant was used to measure luciferase activity using a dual-luciferase reporter assay system. Firefly luciferase activity was normalized to Renillaluciferase activity to calculate PPRE transcriptional activation activity.

### 2.7 Screening of COX-2 inhibitors

COX-2 inhibitors were screened using a fluorescent probe, as follows. Compounds were co-incubated with COX-2 assay buffer, COX-2 probe, cofactor, substrate, and rhCOX-2 in a black 96-well plate. The cyclooxygenase activity of COX-2 oxidized the substrate, generating a fluorescent signal (excitation wavelength 560 nm/emission wavelength 590 nm) that was measured by a microplate reader. The inhibitory effects of different compounds on COX-2 were determined by comparing fluorescence values with those of the full-enzyme control group without added compounds.

#### 2.7.1 Molecular docking

The molecular structures of the compounds in mol2 format were retrieved from PubChem. The crystal structure of the COX-2 protein was sourced from the Protein Data Bank (PDB ID: 5IKQ) (https://www.rcsb.org). The docking protocol was aligned with previous methodologies (Jia et al., 2022) and successfully docked binding sites were utilized as starting points for molecular dynamics simulations.

#### 2.7.2 Molecular dynamics simulations

The protein-ligand complex was parameterized using the AMBER14SB force field and TIP3P water model, and the system was neutralized with Na^+^/Cl^−^ ions. Energy minimization was conducted using the conjugate gradient method, followed by a 300 ps NPT equilibration at 298 K and 1.0 bar. A 150 ns production molecular dynamics simulation was then run with a 2 fs time step to sample the dynamic behavior of the system. The stability and dynamics of the protein-ligand complex were assessed by Root Mean Square Fluctuation (RMSF), hydrogen bonding networks, and Free Energy Landscape (FEL) calculations. The Molecular Mechanics Poisson–Boltzmann Surface Area (MM-PBSA) method was employed to estimate the absolute binding free energy (ΔGbind), with snapshots extracted from the molecular dynamics simulation.

APO-referenced analysis of COX2 orthosteric pocket modulation: The APO system was built from 5IKQ Biological Assembly 1. Crystal hetero-components were removed while retaining the protein dimer to match sec.pdb and chi.pdb. AMBER99SB-ILDN/TIP3P, 0.15 M NaCl and 310 K were used for minimization, NVT, NPT and a 150 ns production run. Each state contributed 151 frames aligned on 1,102 common Cα atoms. The canonical pocket comprised 18 residues within 5 Å of any JMS heavy atom. Cα RMSF, local free volume, Arg120–Tyr355 and Tyr355–Ser530 distances, polar-contact occupancy and remote-to-orthosteric DCCM coupling were compared.

#### 2.7.3 Cell-based COX-2 inhibition assay

RAW264.7 macrophages were stimulated with LPS (100 ng/mL) for 1 h, followed by treatment with C41, C136, or celecoxib for 24 h. PGE_2_ levels in culture supernatants were measured using a PGE_2_ ELISA kit.

#### 2.7.4 Pocket prediction and visualization

PyMOL version 3.1 software was used to visualize molecular docking results. Then, we used specific software (https://proteins.plus) to predict potential active sites online and calculate relevant pocket parameters.

### 2.8 Statistical analysis and software applications

Data illustrating the therapeutic efficacy of MLN against MASLD and its underlying mechanisms are expressed as mean ± standard error of the mean (SEM). Statistical evaluations were performed using one-way analysis of variance (ANOVA) followed by least significant difference (LSD) *post hoc* multiple comparisons, with a significance threshold set at *p* < 0.05.

Liquid chromatograms and total ion chromatogram (TIC) plots were generated using Origin 2021 software. Raw data were processed for peak integration using SCIEX MultiQuant™ software (v3.0.3) and subsequently analyzed by MetaboAnalyst 6.0 (https://www.metaboanalyst.ca). Data visualizations were created using OmicStudio tools (https://www.omicstudio.cn/tool) and the Bioinformatics Platform (https://www.bioinformatics.com.cn), with additional statistical analyses performed in R software (v4.4.1).

## 3 Results

### 3.1 Comprehensive analysis of chemical constituents of MLN

#### 3.1.1 Systematic identification of chemical constituents based on UHPLC-Q-Exactive Orbitrap HRMS

To comprehensively characterize the chemical constituents of MLN, this study employed UHPLC-Q-Exactive Orbitrap HRMS to establish a systematic analytical strategy. Initially, an extensive literature review was conducted by searching the SciFinder database of the American Chemical Society, the PubMed database of the National Center for Biotechnology Information (NCBI), and the China National Knowledge Infrastructure (CNKI) database of Tsinghua University. Data on compounds related to the herbal components of MLN, including ABR, SR, DC, LJF, and LF, were systematically collected and organized. The database included compound name, chemical formula, exact molecular weight, primary fragments commonly detected by mass spectrometry, and secondary fragmentation information for each compound.

Under both positive and negative ionization modes, TIC of MLN demonstrated excellent separation of individual compounds (Figures 1A,B). Through comprehensive comparison of retention times (tR), exact molecular weights, and mass spectral fragmentation patterns, combined with direct comparison against 66 reference standards (Supplementary Table S8), a total of 143 compounds were confidently identified. Analysis of TIC profiles for the five individual medicinal materials (Supplementary Figure S2) revealed that LJF, LF, DC, SR, and ABR contributed 86, 79, 58, 44, and 38 chemical constituents, respectively, indicating both extensive overlap and species-specific chemical characteristics among the constituent herbs.

**FIGURE 1:**
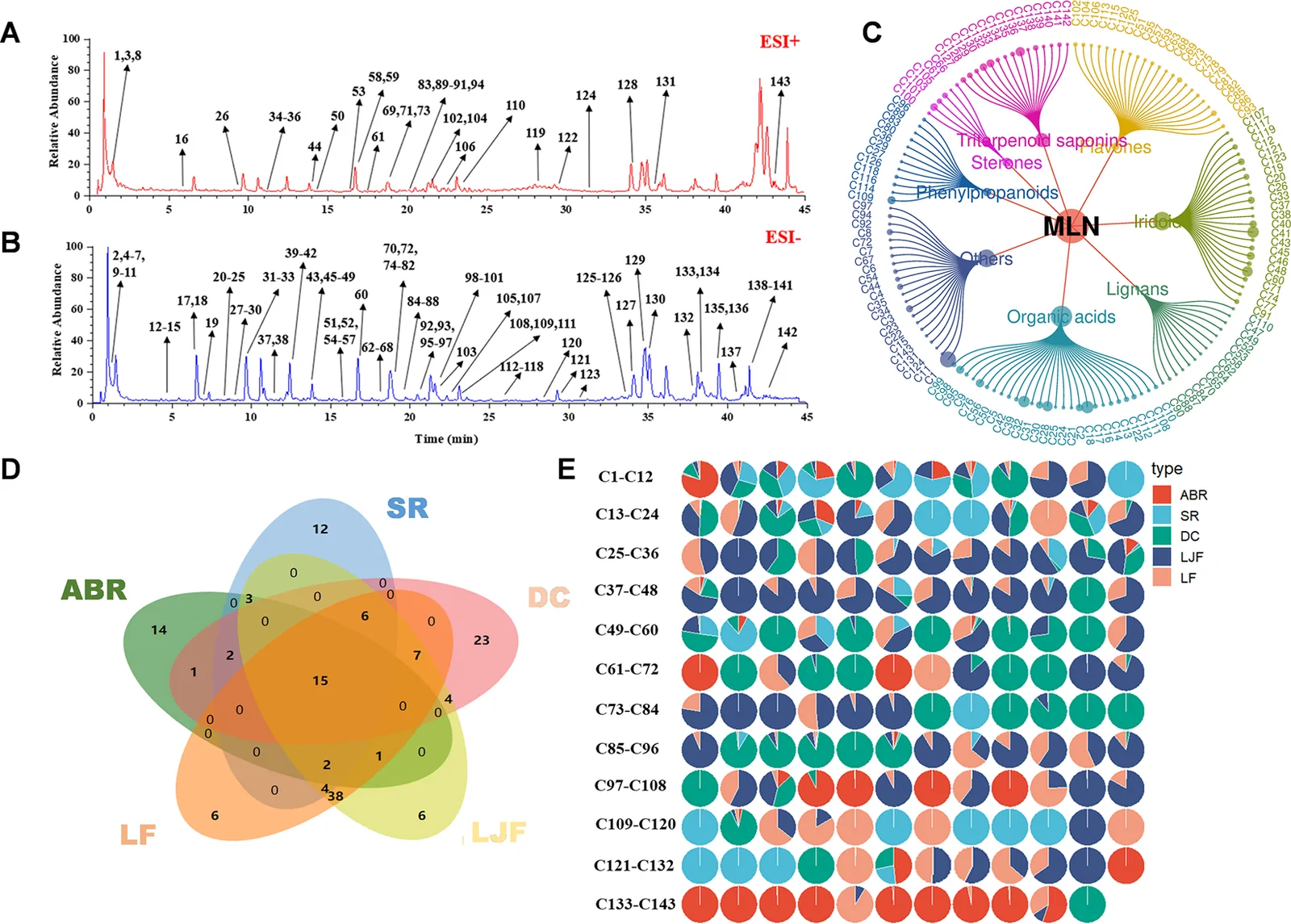
Total ion chromatogram (TIC) of Mailuoning oral liquid (MLN), as well as the types of chemical constituents identified from MLN and the individual herb attribution for each constituent. **(A)** TIC of MLN in positive ion mode. **(B)** TIC of MLN in negative ion mode. **(C)** Types of compounds identified in MLN. **(D)** Venn diagram of the herbal sources of the components of MLN. **(E)** Pie chart of the proportion of individual herb sources for each compound in MLN (based on peak area).

The identified compounds encompassed diverse structural classes, primarily including 28 organic acids, 23 flavonoids, 23 iridoids, 15 triterpenoids, 14 lignans, 12 phenylpropanoids, 6 sterones, 2 bibenzyls, and 20 other compounds (Figure 1C). Visualization analysis (Figures 1D,E) further elucidated the chemical characteristics of each medicinal material: only 15 components were shared among all five herbs. ABR was characterized by steroidal saponins and sterones; SR was enriched in phenylpropanoids and iridoids; DC featured flavonoids, lignans, and bibenzyls as distinctive constituents. The two Lonicera species, LJF and LF, exhibited highly similar chemical profiles, both abundant in organic acids, flavonoids, and triterpenoid saponins.

#### 3.1.2 Analysis *in vivo* exposure characteristics: Blood-entering and liver-entering components

To identify potential pharmacodynamic material bases, we further analyzed the components present in rat serum and liver following MLN administration. Serum and liver samples from the MLN group (10.8 mL/kg/day) and the HFHFD group were analyzed, and peak areas were recorded. Compared with the ions already present in the model group, 48 additional compounds were reproducibly detected in serum from the MLN group and were defined as blood-entering components. Similarly, 92 compounds absent in the livers of model group but detected after MLN treatment were defined as liver-entering components (Supplementary Table S8). Representative extracted ion chromatograms of serum and liver (including HFHFD model, and MLN-treated groups) are shown in Figures 2A–D.

**FIGURE 2:**
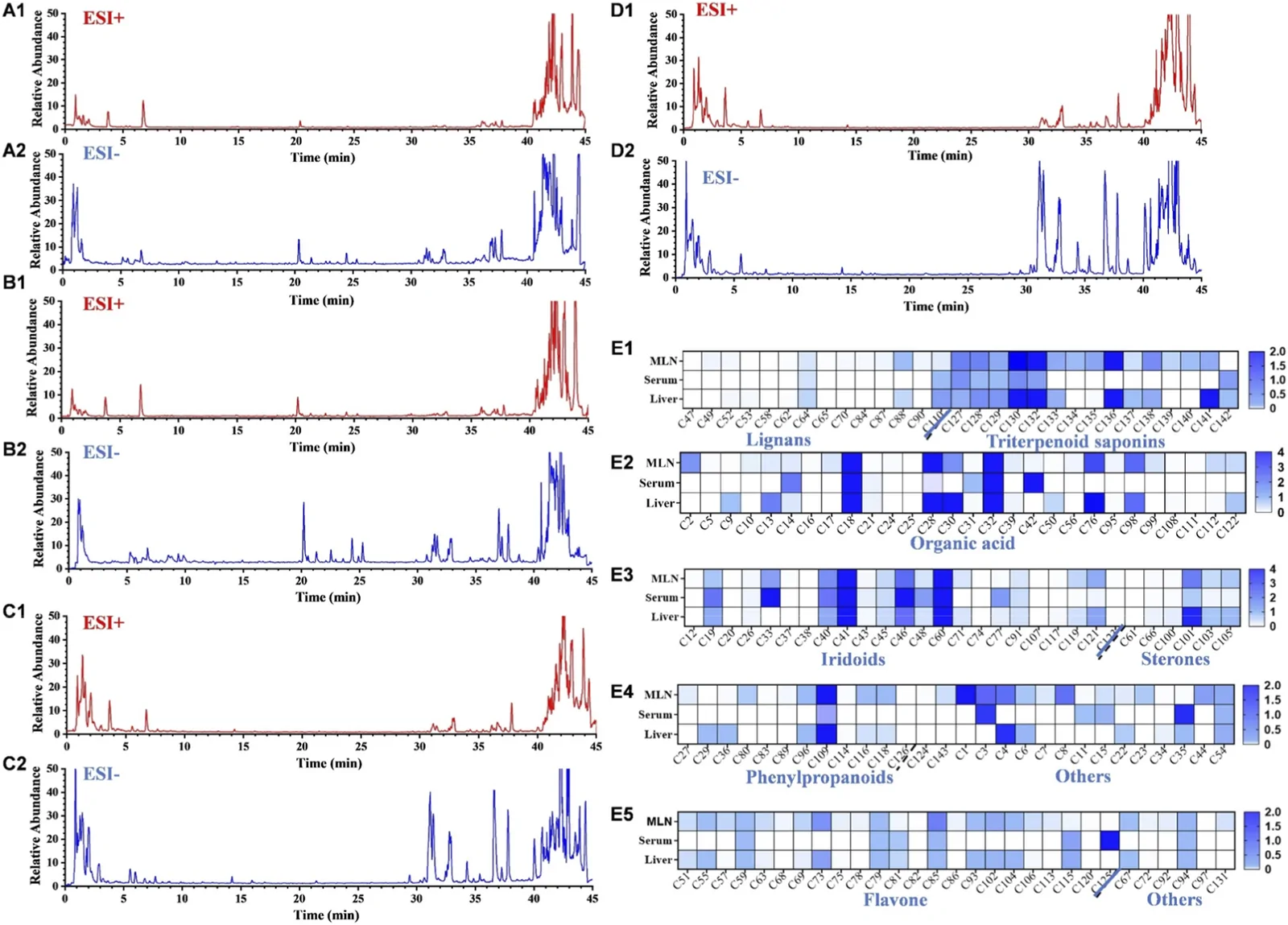
Analysis of *in vivo* exposure components of high-fat high-fructose high-cholesterol diet (HFHFD) and Mailuoning oral liquid (MLN) + HFHFD groups. **(A)** Representative total ion chromatogram (TIC) chromatograms of serum samples from the HFHFD group in positive (A1) and negative (A2) ion modes. **(B)** Representative TIC chromatograms of serum sample from the MLN + HFHFD group in positive (B1) and negative (B2) ion modes. **(C)** Representative TIC chromatograms of liver sample from the HFHFD group in positive (C1) and negative (C2) ion modes. **(D)** Representative TIC chromatograms of liver sample from the MLN + HFHFD group in positive (D1) and negative (D2) ion modes. **(E)** Heatmap showing the categories and relative abundances of MLN-derived components detected in serum and liver. E1. Lignans and triterpenoid saponins; E2. Organic acid; E3. Iridoids and sterones; E4. Phenylpropanoids and others; E5. Flavone and others). Color intensity (blue scale, 0–4) indicates relative abundance, with darker blue representing higher levels.

A heatmap was generated to visualize the categories and relative abundances of blood-entering and liver-entering components (Figure 2E). In the complete MLN formula, iridoids (20.65%), organic acids (19.58%), flavonoids (16.08%), constituted the highest proportions. In the target organ liver, flavonoids (17.39%) and organic acids (19.57%) were similarly the predominant component classes. In serum, iridoids accounted for the highest proportion (31.25%). Notably, secoxyloganin (**C60**) and secologanoside (**C41**) were the two iridoid components with the highest exposure levels in both serum and liver.

### 3.2 MLN ameliorates HFHFD-Induced MASLD metabolic phenotype and liver injury

To evaluate the therapeutic efficacy of MLN against MASLD, male Sprague-Dawley rats were fed an HFHFD for 16 weeks to induce early-stage MASLD, followed by drug intervention during the last 8 weeks. Animals were randomly divided into the following groups: normal chow diet (NCD) control, HFHFD model, MLN low-dose (5.4 mL/kg/d), MLN high-dose (10.8 mL/kg/d), FF (100 mg/kg/d) as positive control and MLNE (10.8 mL/kg/d) (Figure 3A). During the experimental period, no significant differences in food intake or body weight were observed among the NCD, HFHFD, and MLN-treated groups, whereas the FF group exhibited body weight reduction after 4 weeks of administration (Supplementary Figure S3).

**FIGURE 3:**
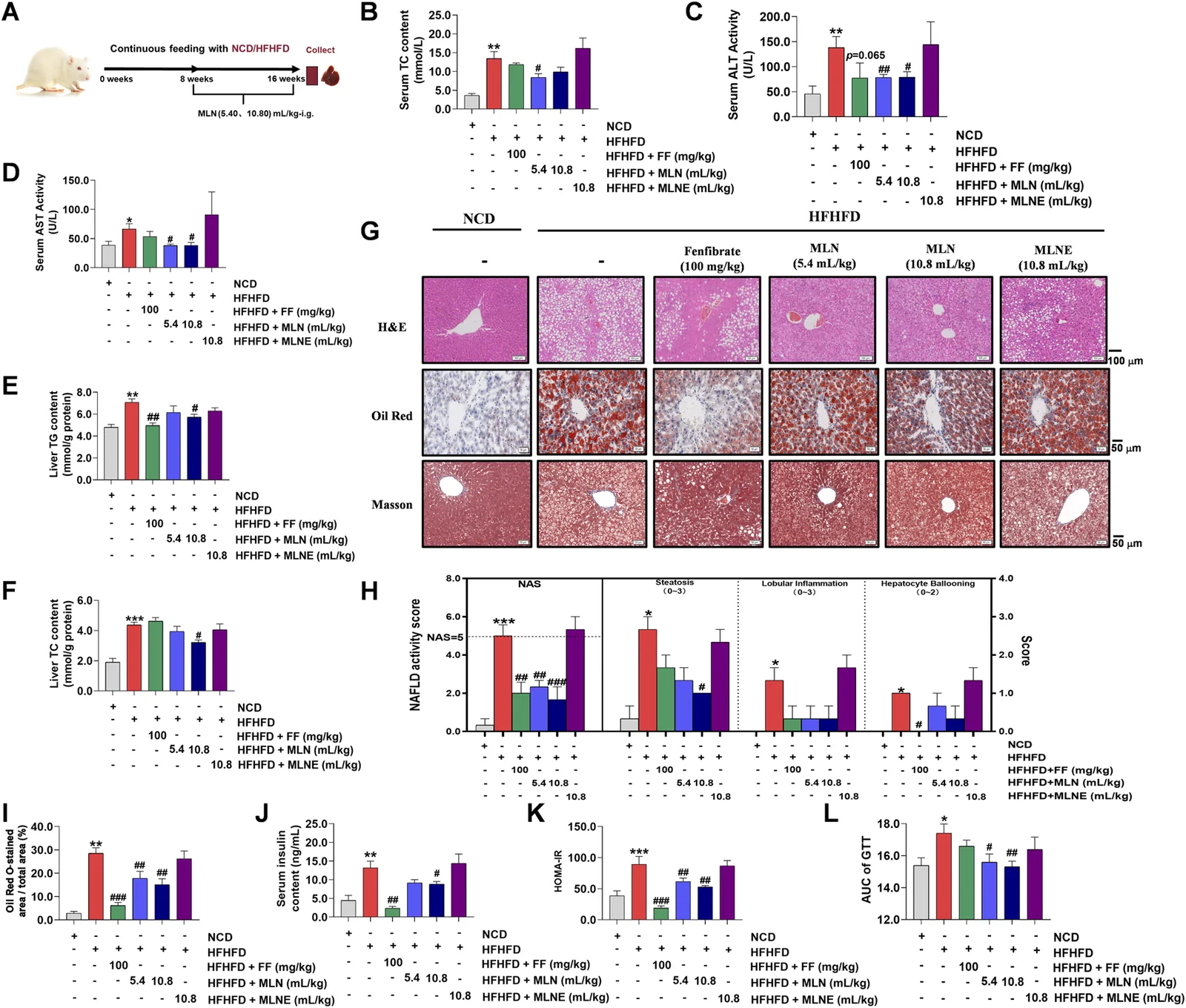
Mailuoning oral liquid (MLN) alleviates hepatic steatosis and metabolic dysfunction in high-fat high-fructose high-cholesterol diet (HFHFD)-fed rats. **(A)** Animal experiment schedule. **(B)** Serum total cholesterol (TC) levels (*n* = 5–6). **(C)** Serum alanine aminotransferase (ALT) activity (*n* = 5–6). **(D)** Serum aspartate aminotransferase (AST) activity (*n* = 6). **(E)** Hepatic triglyceride (TG) levels (*n* = 5–6). **(F)** Hepatic total cholesterol (TC) levels (*n* = 6). **(G)** Representative liver sections stained with HE, Oil Red O and masson (n = 3). **(H)** the quantitative analysis of NAS (*n* = 3). **(I)** Quantification of the percentage of Oil Red O-positive area (*n* = 3). **(J)** Serum insulin levels (*n* = 5–6). **(K)** HOMA-IR value (*n* = 5–6). **(L)** Area under the curve (AUC) of the oral glucose tolerance test (OGTT) (*n* = 6). Data = Mean ± S.E.M, *p < 0.05, **p < 0.01, ***p < 0.001 vs NCD; #*p* < 0.05, ##*p* < 0.01, ###*p* < 0.001 vs HFHFD, MLNE: ns vs HFHFD.

#### 3.2.1 MLN attenuates hepatic lipid accumulation and injury

MLN administration significantly reduced the metabolic dysregulation and liver injury induced by HFHFD. Compared to the HFHFD model group, treatment with MLN significantly lowered serum TC levels (5.4 mL/kg, *p* < 0.05; Figure 3B), ALT (5.4 and 10.8 mL/kg; *p* < 0.01, *p* < 0.05; Figure 3C) and AST (5.4 and 10.8 mL/kg; *p* < 0.05, *p* < 0.05; Figure 3D) levels. The HFHFD for 16 weeks induced severe hepatic steatosis and significant infiltration of inflammatory cells, as evidenced by histology. MLN treatment (10.8 mL/kg) significantly reduced the elevated levels of liver TG (*p* < 0.05; Figure 3E) and TC (*p* < 0.05; Figure 3F) levels. The positive control, FF (100 mg/kg) also reduced liver TG levels (*p* < 0.01) but had no significant effect on hepatic TC. Consistently, HE and Oil Red O staining confirmed extensive lipid accumulation in the HFHFD group, which was markedly alleviated by both MLN doses (5.40, 10.8 mL/kg) and FF (100 mg/kg) treatment (Figures 3G–I). Masson staining was used to evaluate collagen deposition in the liver. The results showed no significant differences among the groups (Figure 3G), indicating that the model was restricted to the early stage of MASH and had not yet progressed to hepatic fibrosis.

#### 3.2.2 MLN improves glucose-lipid homeostasis and insulin sensitivity

MLN also ameliorated HFHFD-induced glucose metabolism abnormalities. Administration of MLN (10.8 mL/kg) significantly reduced insulin levels (*p* < 0.05), and decreased HOMA-IR values compared with HFHFD group indicating that MLN alleviated insulin resistance in HFHFD rats (Figures 3J,K). A similar beneficial effect was also observed with FF treatment (100 mg/kg; *p* < 0.01), whereas MLNE showed no significant effects (Figures 3J,K).Furthermore, HFHFD feeding for 16 weeks induced significant glucose intolerance, which was alleviated by 8-week of MLN treatment (5.4, 10.8 mL/kg; *p* < 0.05, *p* < 0.01; Figure 3L). In contrast, FF and MLNE administration did not significantly alter glucose tolerance.

### 3.3 Lipidomics reveals the remodeling effect of MLN on hepatic lipid metabolism

The above improvements in metabolic phenotype and hepatic injury suggest that MLN exerts significant regulatory effects on hepatic lipid metabolism. To elucidate the underlying molecular mechanism, this study employed a pseudo-targeted lipidomics platform to comprehensively analyze rat liver and serum lipids. The established platform detected a total of 1,188 lipid molecules, encompassing six major lipid classes, with glycerophospholipids (609 species, 51.3%) and TAG (415 species, 35.0%) accounted for 86.3% of total detected lipids, systematically covering key biological processes including energy storage, membrane structure maintenance, and signal transduction.

#### 3.3.1 Bidirectional regulation of hepatic TAG accumulation and membrane phospholipid depletion

A total of 448 lipid molecules were detected in rat liver (Supplementary Table S5), including glycerolipids (213 species), glycerophospholipids (184 species), sphingolipids (23 species), fatty acyls (23 species), sterol lipids (14 species), and glycosyl lipids (1 species) (Figure 4A). HFHFD feeding induced significant alterations in the hepatic lipid profile (Figure 4B). Compared with the NCD group, 325 differentially expressed lipids were detected in the HFHFD group (219 upregulated, 106 downregulated; FC > 1.5 or <0.67, *p* < 0.05). The pathological features were characterized by massive deposition of TAG (130 upregulated TAG species, accounting for 59.4% of upregulated lipids) accompanied by depletion of specific glycerophospholipids, including PE, PC, phosphatidylglycerol (PG), phosphatidic acid (PA), phosphatidylserine (PS), and lysophosphatidy-lethanolamine (LPE) (Figure 4C), presenting a typical MASLD hepatic lipid profile of “energy-storage lipid accumulation vs. structural lipid depletion.”

**FIGURE 4:**
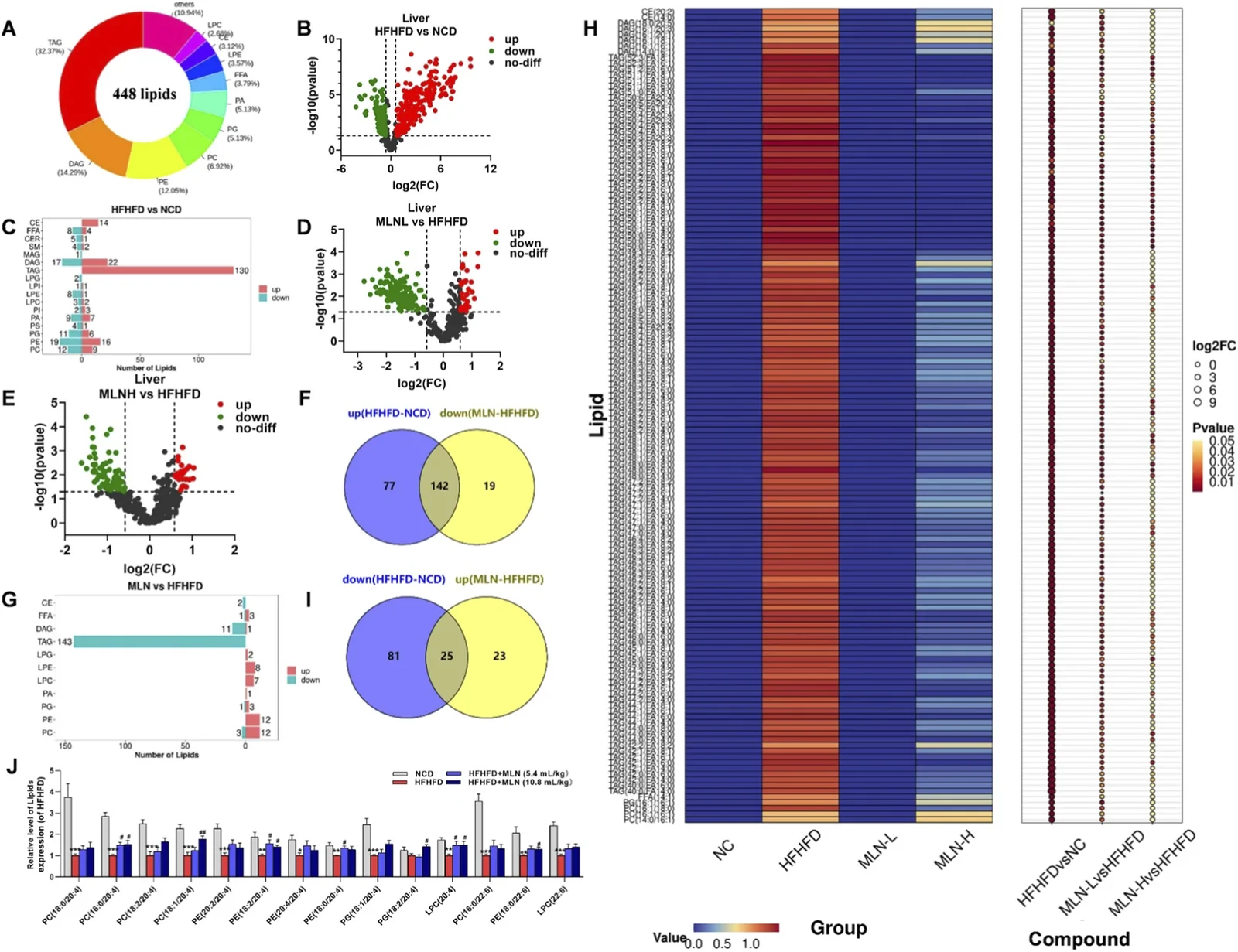
Hepatic lipidomics reveals Mailuoning oral liquid (MLN)-mediated bidirectional regulation of lipid metabolism. **(A)** Composition of 448 lipid species detected in liver. **(B)** Volcano plot of hepatic lipids differentially expressed in high-fat high-fructose high-cholesterol diet (HFHFD) vs. normal chow diet (NCD). Red: upregulated; green: downregulated. **(C)** Number of up/downregulated lipids within each class in HFHFD vs. NCD. **(D, E)** Volcano plots of hepatic lipids after MLN treatment at (D) 5.4 mL/kg and (E) 10.8 mL/kg vs. HFHFD. **(F)** Venn diagram: hepatic lipids upregulated by HFHFD and downregulated by MLN (combined doses). **(G)** Number and class distribution of downregulated lipids from (F) and upregulated lipids from (I) **(H)** Heatmap showing relative abundance of the 142 hepatic lipids upregulated by HFHFD and downregulated by MLN across experimental groups. Data are Z-score normalized, with color indicating relative lipid levels (blue: low, red: high). **(I)** Venn diagram: hepatic lipids downregulated by HFHFD and upregulated by MLN (combined doses). **(J)** Relative levels of key arachidonic acid (ARA)- and docosahexaenoic acid (DHA)-containing phospholipids in liver. Data = Mean ± S.E.M (*n* = 5), *p < 0.05, **p < 0.01, ***p < 0.001 vs NCD; #*p* < 0.05, ##*p* < 0.01 vs HFHFD.

MLN intervention effectively reversed this lipid imbalance. Compared with the HFHFD model group, both doses of MLN (5.4 and 10.8 mL/kg) induced extensive and diverse changes in the lipid profiles (Figures 4D,E). Notably, in terms of overall improvement, the low dose of MLN (5.4 mL/kg) exhibited a superior regulatory trend compared to the high dose (10.8 mL/kg). Integrating the regulatory effects of both doses, MLN collectively and significantly reduced 142 hepatic lipids that were upregulated in the HFHFD model (Figure 4F), including 129 TAGs, 6 diglycerides, and certain cholesteryl esters, such as cholesteryl esters (18:1) (Figure 4G). A heatmap analysis of these 142 downregulated lipids further revealed that HFHFD feeding significantly elevated their hepatic levels, and MLN treatment effectively reversed these changes, restoring their levels toward those in the NCD group (Figure 4H). Specifically, in correcting the abnormal accumulation of certain lipids (e.g., some cholesteryl esters), the low dose of MLN demonstrated a more targeted regulatory effect than the high dose. This indicates that MLN effectively alleviates excessive hepatic TAG accumulation and improves lipid metabolic disorders, with a complex dose-response relationship.

On the other hand, MLN significantly upregulated 25 membrane phospholipids that were downregulated by HFHFD (Figure 4I), primarily including PC, PE, LPE, and PG (Figure 4G). Notably, among these restored phospholipids, 11 species containing ARA (20:4) acyl chains, such as PC (16:0/20:4) and PE (18:0/20:4), were identified. These phospholipids serve as the primary intracellular storage form for lipid signaling molecules. Concurrently, MLN specifically reversed the depletion of 3 membrane phospholipids containing DHA (22:6) acyl chains, such as PE (18:0/22:6). As shown in Figure 4J, MLN dose-dependently and significantly restored the levels of these key ARA- and DHA-containing phospholipids, and the low dose of MLN also showed a stronger effect in restoring some DHA-phospholipids. DHA is a critical structural fatty acid for maintaining membrane fluidity and function.

#### 3.3.2 Serum lipidomics reveals limited response in peripheral circulation

To investigate whether hepatic lipid alterations are reflected in the peripheral circulation, we performed parallel serum lipidomics analysis. A total of 203 lipid species were detected in serum (equivalent to 45.3% of the hepatic lipidome), including 90 glycerophospholipids, 88 glycerolipids, 19 fatty acyls, 13 sphingolipids, 8 sterol lipids, and 1 glycosyl lipid (Figure 5A). HFHFD feeding induced significant alterations in the serum lipid profile (Figure 5B). Compared with the NCD group, 102 serum lipids were differentially expressed in the HFHFD group, with 74 upregulated and 28 downregulated (Figure 5C). In contrast to the extensive lipid remodeling observed in the liver, MLN intervention elicited a more limited modulation of the serum lipid profile. While the lipidomic profiles modulated by the two doses of MLN (5.4 and 10.8 mL/kg) showed partial overlap, the overall magnitude of change was modest (Figures 5D,E). Further analysis identified 19 key serum lipids associated with both MASLD development and MLN intervention (Figure 5F). Notably, all 19 key lipids showed a downregulation trend following MLN treatment, with no significantly upregulated lipids detected. As shown in Figure 5G, HFHFD feeding significantly elevated serum levels of certain cholesteryl ester, such as cholesteryl ester (18:1), and phospholipids, and MLN treatment effectively reversed these changes, restoring the levels of these key lipids toward those in the NC group. These 19 downregulated lipids were predominantly PC and PE species containing specific saturated and monounsaturated acyl chains, such as C16:0, C18:0, C16:1, and C18:1. MLN dose-dependently and significantly reduced the serum levels of these specific PC and PE subtypes.

**FIGURE 5:**
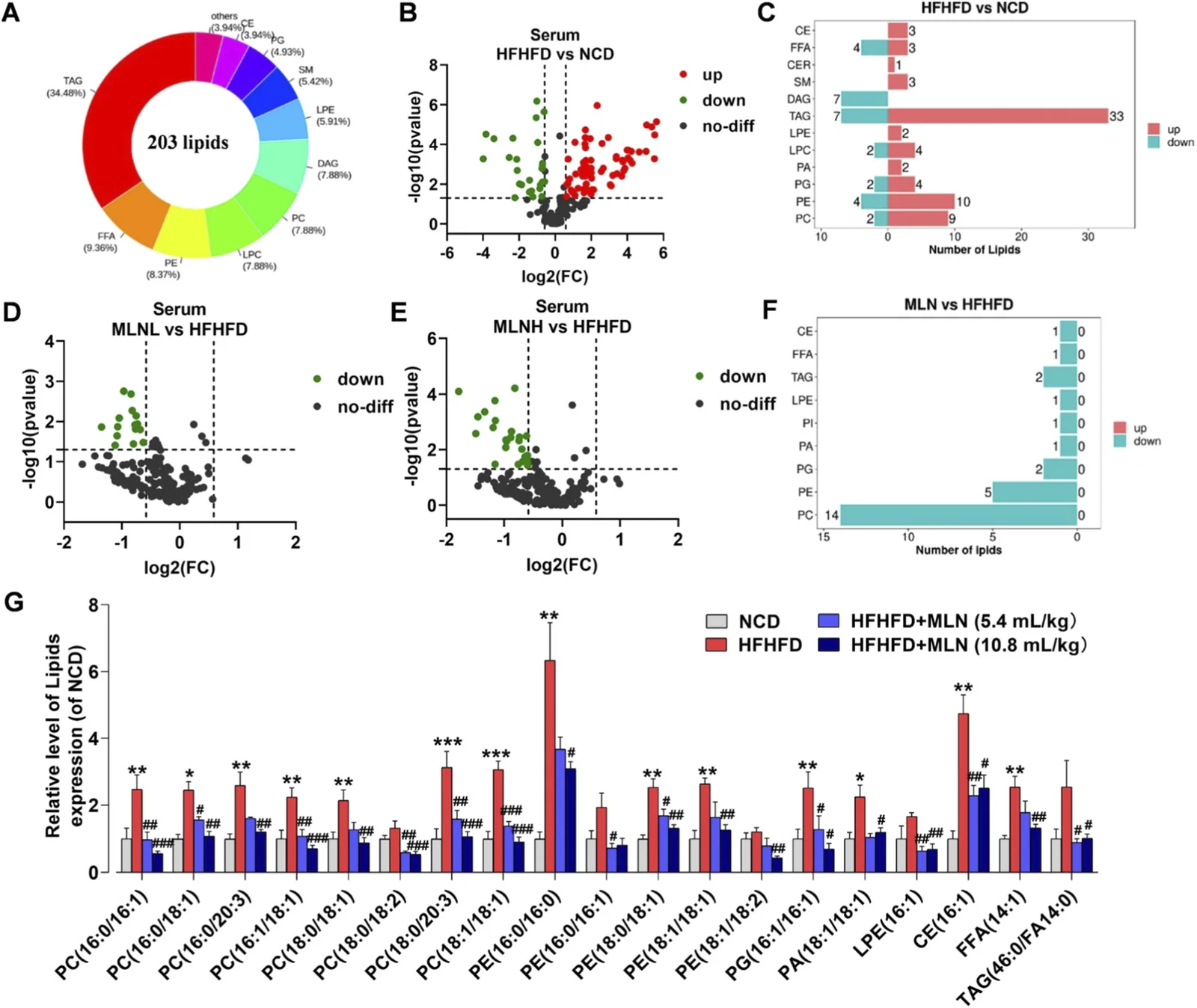
Serum lipidomic analysis. **(A)** Composition of the 203 lipid species detected in serum. **(B)** Volcano plot of serum lipids differentially expressed between the high-fat high-fructose high-cholesterol diet (HFHFD) and normal chow diet (NCD) groups. Red: up; green: down. **(C)** Number and class of up- and downregulated serum lipids in HFHFD vs. NCD. **(D, E)** Score plots of serum lipid profiles after Mailuoning oral liquid (MLN) (D) 5.4 mL/kg and (E) 10.8 mL/kg treatment, compared to the HFHFD model. **(F)** Venn diagram identifying serum lipids commonly associated with MASLD pathogenesis (HFHFD vs. NCD) and MLN intervention (MLN vs. HFHFD). **(G)** Serum levels of 19 specific phosphatidylcholine (PC)/phosphatidylethanolamine (PE) subspecies containing C16:0, C18:0, C16:1, or C18:1 acyl chain. Data = Mean ± S.E.M (*n* = 5), *p < 0.05, **p < 0.01, ***p < 0.001 vs NCD; #*p* < 0.05, ##*p* < 0.01, ###*p* < 0.001 vs HFHFD.

Although the number and magnitude of differential lipids in serum were substantially lower than in the liver, the identified specific PC subtypes (containing C16:0/C18:0 acyl chains) partially overlapped with the characteristics of hepatic membrane phospholipid remodeling. This suggests that these serum phospholipids may serve as potential non-invasive biomarkers reflecting hepatic membrane lipid synthesis status. However, the pronounced and predominant TAG accumulation observed in the liver (130 differential TAGs) was not adequately reflected in the serum. This finding indicates that for early MASLD screening, monitoring changes in serum structural phospholipids (e.g., PC and PE with specific acyl chain compositions) may provide greater indicative value than measuring classical neutral lipids (TAG, DAG), thereby compensating for the latter’s limited sensitivity in detecting early hepatic steatosis.

#### 3.3.3 PPARα pathway activation mediates fatty acid oxidation and lipid homeostasis restoration

The lipidomics-observed reduction in TAG and restoration of membrane phospholipids, particularly the increase in ARA-enriched phospholipids, suggests that MLN may regulate hepatic lipid metabolism by activating PPARα pathway. Given that PPARα is a key transcription factor in lipid metabolism, and our previous study has demonstrated that MLN can ameliorate MASLD through PPARα activation (Jia et al., 2022), this study further validated the central role of this pathway in the HFHFD-induced MAFLD model.

Western blot analysis showed that MLN (10.8 mL/kg) intervention significantly promoted PPARα nuclear translocation (Figure 6A). qPCR results further demonstrated that MLN significantly upregulated the mRNA expression of PPARα and its key downstream fatty acid oxidation genes, including Cpt1a, acyl-CoA dehydrogenase short chain (Acads), acyl-CoA dehydrogenase medium chain (Acadm), acyl-CoA dehydrogenase long chain (Acadl), and acyl-CoA dehydrogenase very long chain (Acadvl) (Figures 6B–G). These molecular changes corroborate the lipidomics phenotype of “reduced TAG accumulation and restored membrane phospholipids.” Together, they constitute a PPARα-mediated mechanism for the restoration of lipid homeostasis: activating the fatty acid β-oxidation pathway to reduce TAG accumulation, while potentially promoting the synthesis of specific membrane phospholipids (including ARA-phospholipids) to restore hepatocyte membrane structure and functional integrity.

**FIGURE 6:**
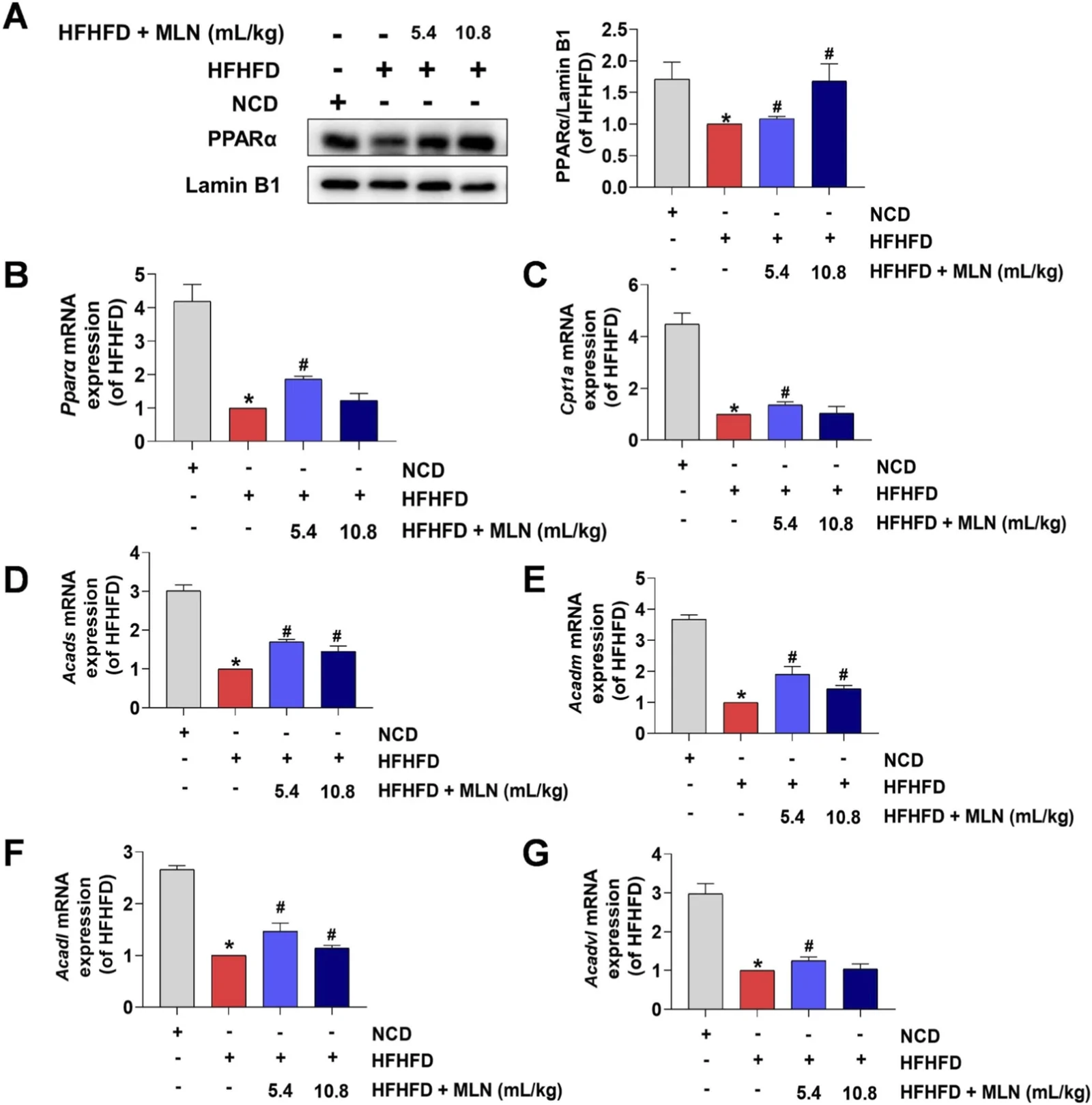
Mailuoning oral liquid (MLN) activates the hepatic peroxisome proliferator-activated receptor α (PPARα) signaling pathway. **(A)** Representative western blot images (left) and quantitative analysis (right) of nuclear PPARα protein levels in the liver. Lamin B1 served as the loading control for the nuclear fraction. (*n* = 3). **(B–G)** Hepatic mRNA levels of *Pparα* **(B)**, carnitine palmitoyltrans-ferase 1A (*Cpt1a*) **(C)**, Acyl-CoA dehydrogenase short chain (*Acads*) **(D)**, Acyl-CoA dehydrogenase medium chain (*Acadm*) **(E)**, Acyl-CoA dehydrogenase long chain (Acadl) **(F)**, and Acyl-CoA dehydrogenase very long chain (*Acadvl*) **(G)** determined by qPCR. Data are presented as mean ± SEM (*n* = 4). *p < 0.05 vs NCD; #*p* < 0.05 vs HFHFD.

#### 3.3.4 MLN precisely regulates PUFAs metabolism and selectively inhibits the COX-2 pathway

Lipidomics analysis has revealed that MLN significantly remodels hepatic phospholipid profiles, particularly enriching ARA-containing species. As the predominant PUFA in membrane phospholipids, ARA constitutes the central hub of the PUFA metabolic network, serving as the primary substrate for eicosanoid synthesis via COX, lipoxygenase (LOX), and cytochrome p450 (CYP450) pathways. Expansion of ARA-containing phospholipid pools may therefore reflect either increased total PUFA availability or selective redistribution among lipid classes, both with distinct functional implications for inflammatory mediator production. To dissect these possibilities, subsequent experiments simultaneously quantified free PUFA pools and their downstream metabolites, thereby elucidating whether MLN achieves anti-inflammatory effects through selective pathway inhibition or global metabolic suppression.

##### 3.3.4.1 MLN restores PUFAs pools and selectively modulates ARA metabolic flux

Based on the lipidomics finding that MLN significantly upregulates ARA-containing phospholipids, we hypothesized that MLN may exert anti-inflammatory effects by expanding the total ARA pool and regulating its metabolic flux. To test this hypothesis, we established a UHPLC-MS method targeting 16 PUFAs and their metabolites, systematically analyzing the levels of Ω-3/Ω-6 PUFAs and their downstream COX, LOX, and CYP450 pathway metabolites in liver and serum. The chemical structures of the detected PUFAs are shown in Figure 7A.

**FIGURE 7:**
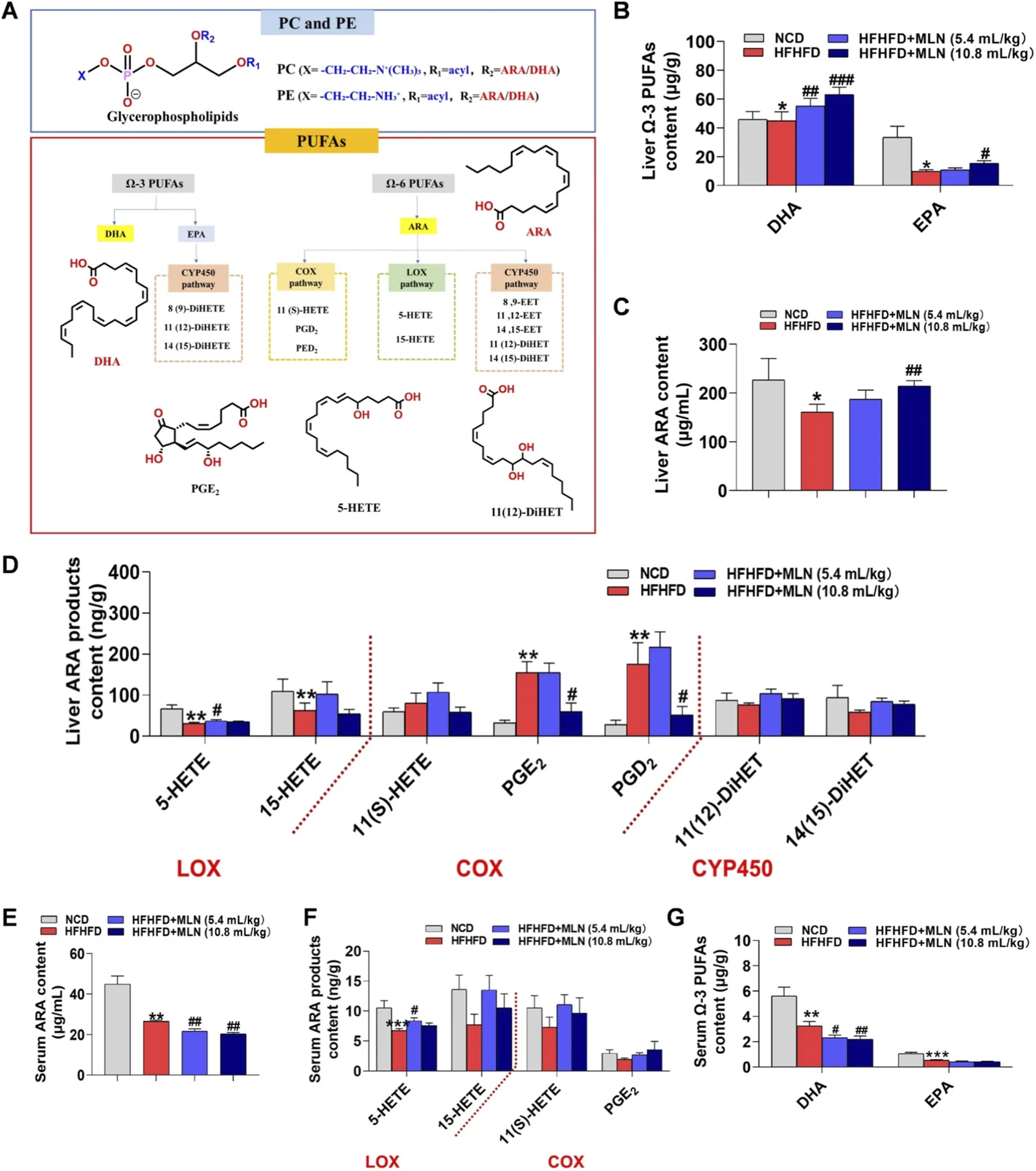
Mailuoning oral liquid (MLN) restores hepatic polyunsaturated fatty acids (PUFAs) pools and selectively modulates arachidonic acid (ARA) metabolism. **(A)** Chemical structures of the detected PUFAs and their downstream metabolites. **(B,C)** Hepatic levels of key Ω-3 PUFAs (B: docosahexaenoic acid (DHA), eicosapentaenoic acid (EPA)) and the Ω-6 PUFA ARA (C). **(D)** Hepatic levels of lipoxygenase (LOX), cyclooxygenase (COX) and cytochrome p450 (CYP450) pathway metabolites. **(E–G)** Serum levels of ARA **(E)**, ARA metabolites of COX and LOX pathway **(F)**, DHA and EPA **(G)**. Data are presented as mean ± SEM (*n* = 5). *p < 0.05, **p < 0.01, ***p < 0.001 vs NCD; #*p* < 0.05, ##*p* < 0.01, ###*p* < 0.001 vs HFHFD.

Ten PUFAs were detected in the livers of HFHFD model rats treated with MLN, including DHA, EPA, ARA, and 7 metabolites (5-HETE, 15-HETE, 11(S)-HETE, PGE_2_, PGD_2_, 11(12)-DiHET, 14(15)-DiHET). Compared with the NCD group, HFHFD induced significant depletion of PUFAs: hepatic levels of DHA, EPA, and ARA were significantly reduced (*p* < 0.05; Figures 7B,C), while levels of the COX pathway products PGE_2_ and PGD_2_ were significantly increased (*p* < 0.01; Figure 7D), reflecting ARA metabolic activation under inflammatory conditions. Following MLN intervention, DHA levels increased dose-dependently (*p* < 0.01, *p* < 0.001), and high-dose MLN also significantly elevated EPA and ARA levels (*p* < 0.05, *p* < 0.01; Figures 7B,C). The restoration of ARA levels was observed in both the membrane phospholipid-bound pool and the free fatty acid pool. Concurrently, MLN significantly reduced the elevated levels of PGE_2_ and PGD_2_ (*p* < 0.05; Figure 7D), while LOX pathway products (5-HETE, 15-HETE) and CYP450 pathway products (11,12-DiHET, 14,15-DiHET) showed no significant differences among groups. This pattern of “pool restoration, COX inhibition, and preservation of alternative pathways” indicates that MLN has a selective inhibitory effect on ARA metabolism, specifically targeting the COX pathway.

In serum, 7 target PUFAs were detected, including DHA, EPA, ARA, and 4 metabolites (5-HETE, 15-HETE, 11(S)-HETE, PGE_2_). Compared with the NCD group, serum levels of DHA, EPA, ARA, and the LOX pathway metabolite 5-HETE were significantly lower in the HFHFD group (*p* < 0.01 or *p* < 0.001; Figures 7E–G). However, after MLN administration, only 5-HETE was significantly restored (*p* < 0.05; Figures 7E–G), with no significant effects on the other PUFAs. This reveals the tissue-specific nature of MLN’s regulatory effects: comprehensively restoring PUFAs pools and selectively inhibiting COX pathway activation in the liver, while primarily modulating the LOX metabolic branch of ARA in the peripheral circulation.

##### 3.3.4.2 MLN ameliorates hepatic inflammation and modulates the COX-2 pathway in MASLD

To verify that the observed inhibition of the COX metabolic pathway effectively blocks downstream inflammatory cascades and elucidate its molecular basis, we further examined the effects of MLN on hepatic inflammatory phenotypes and the expression of key inflammatory mediators. Consistent with the increase in hepatic COX metabolites (PGE_2_, PGD_2_) induced by HFHFD, livers from HFHFD rats exhibited a marked inflammatory response, evidenced by significantly increased immunostaining-positive area for the macrophage marker F4/80 (Figure 8A) and elevated mRNA levels of the pro-inflammatory cytokine tumor necrosis factor (*Tnf*) (*p* < 0.01; Figure 8B). MLN treatment effectively reversed these inflammatory phenotypes, significantly reducing F4/80^+^ macrophage infiltration and *Tnf* mRNA expression (*p* < 0.05; Figures 8A,B).

**FIGURE 8:**
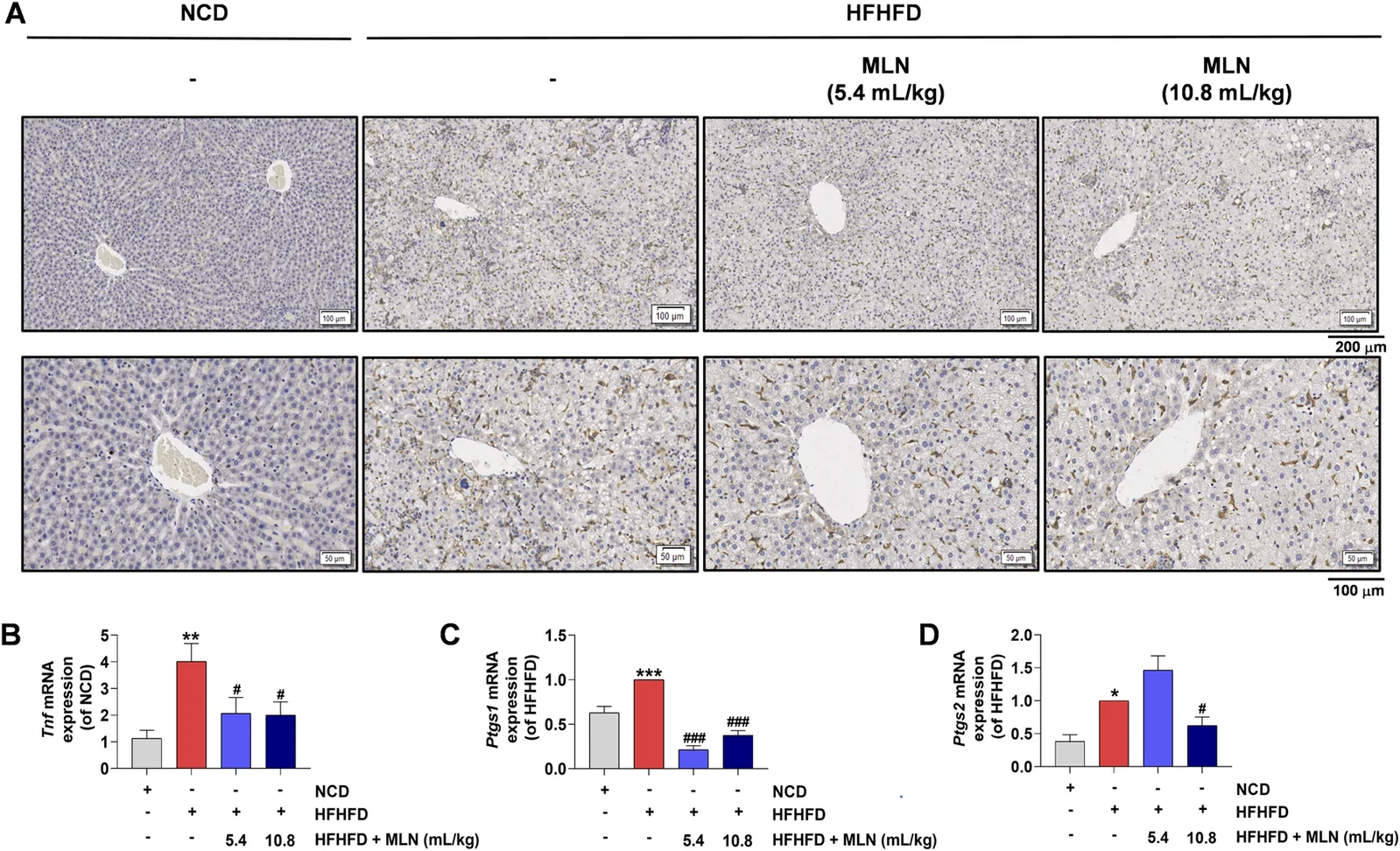
Effects of Mailuoning oral liquid (MLN) on the expression of inflammatory markers in the liver of rats with MASLD induced by a high-fat high-fructose diet (HFHFD). **(A)** Representative immunohistochemical (IHC) staining images of target proteins F4/80^+^ macrophage infiltration in liver tissues from different groups: normal chow diet (NCD), HFHFD model, HFHFD + MLN (5.40 mL/kg), and HFHFD + MLN (10.08 mL/kg). Scale bars: 100 μm (top row) and 50 μm (bottom row). **(B–D)** Quantification of mRNA expression levels of pro-inflammatory cytokines: (B) *Tnf* (tumor necrosis factor-alpha), (C) prostaglandin-endoperoxide synthase (*Ptgs1*), and (D) prostaglandin-endoperoxide synthase (*Ptgs2*) in liver tissues, normalized to the NCD group. Data are expressed as mean ± SEM (*n* = 4). Statistical significance: *p < 0.05, **p < 0.01, ***p < 0.001 vs. NCD; #*p* < 0.05, ###*p* < 0.001 vs. HFHFD.

At the molecular level, HFHFD feeding significantly upregulated the mRNA expression of the rate-limiting enzymes for prostaglandin synthesis, COX-1 (encoded by prostaglandin-endoperoxide synthase 1 (*Ptgs1*)) and COX-2, (encoded by prostaglandin-endoperoxide synthase 2 (*Ptgs2*)) (Figures 8C,D). MLN treatment significantly downregulated the transcriptional levels of both isoforms (*p* < 0.05, *p* < 0.001; Figures 8C,D). The downregulation of the inducible isoform COX-2 (*Ptgs2*) was particularly pronounced, which directly corresponds to the reduction in PGE_2_/PGD_2_ (Figure 7D) and the amelioration of inflammatory phenotypes. This provides evidence at the level of COX gene expression, confirming that MLN exerts its anti-inflammatory effect by preferentially inhibiting the COX-2 pathway, thereby reducing the generation of pro-inflammatory prostaglandins.

### 3.4 Screening for key active components in MLN targeting PPARα and COX-2

Previous integrative studies have demonstrated that MLN ameliorates lipid metabolism by activating PPARα and exerts anti-inflammatory effects by inhibiting COX-2. To further elucidate the direct-acting substance basis underlying these pharmacodynamic activities, this study further screened for active compounds capable of directly targeting PPARα and COX-2, starting from the systemically absorbed (blood) and liver-distributed components of MLN.

#### 3.4.1 Identification of PPARα agonists based on exposure profiles

To identify direct PPARα agonists, blood-exposed constituents of MLN were subjected to integrated screening combining cell-based phenotypic assays, molecular docking, and PPARα reporter gene experiments.

##### 3.4.1.1 Attenuation of lipid accumulation in HepaRG cells

Thirty-four blood-exposed constituents were evaluated for inhibition of NEFA-induced lipid accumulation in HepaRG cells. Ten compounds significantly reduced intracellular lipid content: chlorogenic acid, macranthoidin B, luteoloside, luteolin, 25R-inokosterone, harpagide, angoroside C, acteoside, oleanolic acid, and caffeic acid (Figures 9A,B), demonstrating direct lipid-lowering activity *in vitro*. Detailed information regarding the 34 compounds and the corresponding *in vitro* BODIPY screening results were shown in Supplementary Figure S4.

**FIGURE 9:**
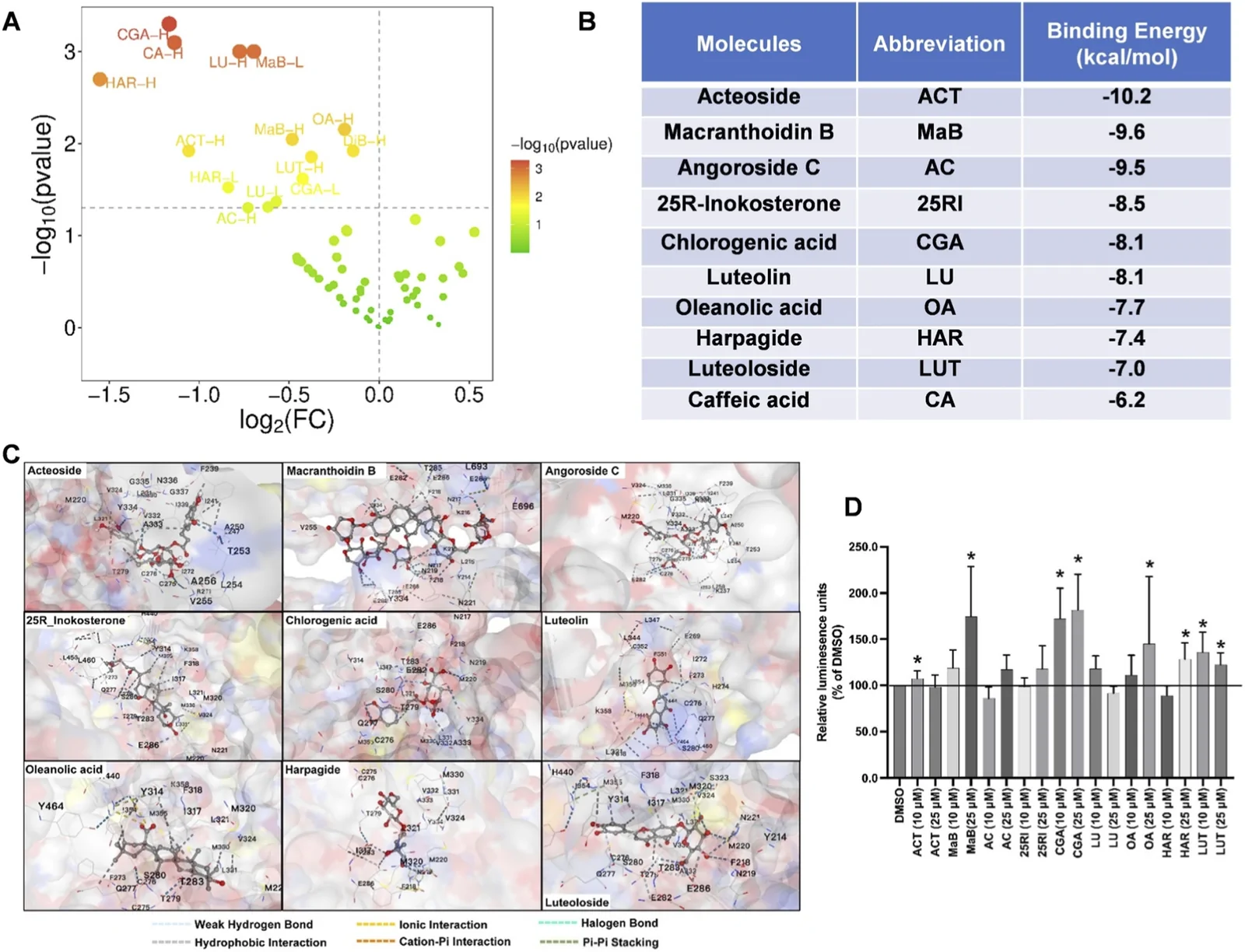
Lipid-lowering activity and PPARα agonist screening of major constituents from Mailuoning oral liquid (MLN). **(A)** Volcano plot of the *in vitro* lipid-lowering screening results; **(B)** Predicted binding energy of the molecular docking analysis; **(C)** Interaction diagrams of molecular docking (acteoside, macranthoidin B, angoroside C, 25R-inokosterone, chlorogenic acid, luteolin, oleanolic acid, harpagide, luteoloside); **(D)** Relative PPRE-luciferase activity. Data are presented as mean ± SEM (*n* = 3). *p < 0.05 *versus* the DMSO group.

##### 3.4.1.2 High-affinity binding to PPARα revealed by molecular docking

Molecular docking analysis demonstrated that 9 of 10 lipid-lowering constituents exhibited binding energies below −7 kcal/mol against the PPARα ligand-binding domain. Acteoside (−10.2 kcal/mol), macranthoidin B (−9.6 kcal/mol), and angoroside C (−9.5 kcal/mol) displayed the strongest binding affinities.

Binding mode analysis revealed distinct interaction patterns. Acteoside, angoroside C, 25R-inokosterone, and chlorogenic acid formed hydrogen bonds with the critical activation residue THR279 via hydroxyl or carboxyl groups, accompanied by hydrophobic interactions with surrounding residues (MET220, PHE239). Macranthoidin B and oleanolic acid established extensive hydrogen bond networks with polar residues; notably, the terminal carboxyl of oleanolic acid directly interacted with THR279. Luteoloside, but not its aglycone luteolin, engaged THR279 through glycosyl hydroxyl groups, indicating a stabilizing effect of glycosylation (Figure 9C). Harpagide exhibited an alternative binding pattern, bypassing THR279 to interact with THR283 and GLU286, suggestive of potential allosteric modulation.

##### 3.4.1.3 Key components activate PPARα transcriptional activity

PPRE-luciferase reporter assays confirmed that chlorogenic acid, macranthoidin B, luteoloside, harpagide, angoroside C, oleanolic acid, and betaine significantly activated PPARα transcriptional activity (Figure 9D), functionally validating their potential as PPARα agonists.

#### 3.4.2 Screening and characterization of key components with COX-2 inhibitory activity

##### 3.4.2.1 Identification of candidate components and enzyme activity inhibition

To investigate whether MLN contains key components capable of directly targeting the COX-2 to mediate selective inhibition and anti-inflammatory effects, high-exposure components were screened for COX-2 inhibitory activity based on their *in vivo* exposure characteristics. Seven components accounting for the top 50% of total hepatic exposure were selected: secoxyloganin (**C60**), secologanoside (**C41**), caffeic acid (**C30**), neochlorogenic acid (**C18**), cryptochlorogenic acid (**C32**), chlorogenic acid (**C28**), and chikusetsusaponin IVa (**C136**) (Figure 10A). Screening results revealed that **C41** and **C136** exhibited potent COX-2 enzyme inhibitory activity at sub-micromolar concentrations, with half-maximal inhibitory concentrations (IC_50_) of 14.61 nM and 120.9 nM, respectively; the positive control celecoxib showed an IC_50_ of 15.16 nM (Figure 10B). Notably, these two active components accounted for substantial proportions of hepatic exposure in MLN, with **C41** representing 9.50% of total exposure, and **C136** being the most highly exposed saponin representative. The cellular COX-2 inhibitory activity of C41 and C136 was further validated in LPS-stimulated RAW264.7 macrophages. C136 significantly reduced PGE_2_ production in a dose-dependent manner, while C41 also significantly suppressed PGE_2_ production at 50 µM (Figure 10C).

**FIGURE 10:**
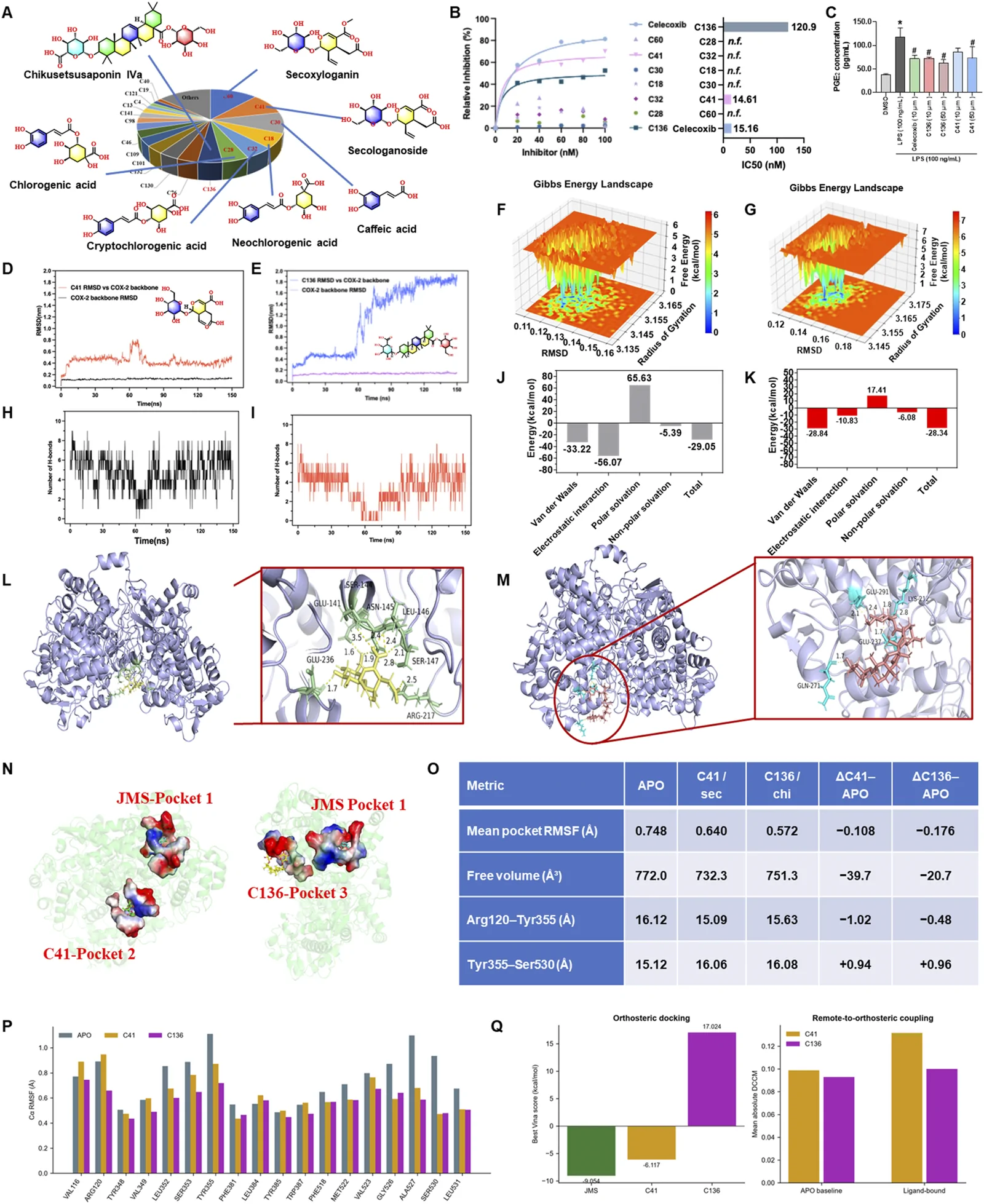
Screening and characterization of COX-2 inhibitors from Mailuoning oral liquid (MLN). **(A)** Chemical structure of seven high-exposure constituents and their proportional distribution in hepatic exposure. **(B)** Dose-response curves of COX-2 inhibition for secologanoside (**C41**), chikusetsusaponin IVa (**C136**), and celecoxib (left), and IC_50_ values of COX-2 inhibition for seven constituents; n.f., not found (IC_50_ > 100 μM) (right). **(C)**. PGE_2_ production in LPS-stimulated RAW264.7 macrophages treated with celecoxib (10 µM), **C136** (10 and 50 µM), or **C41** (10 and 50 µM), measured by ELISA (n = 3). **(D–E)** RMSD of molecular dynamics simulations for C41 (D) and C136 (E) binding to COX-2 backbone. **(F–G)** Gibbs free energy landscapes of C41 (F) and C136 (G) binding to COX-2. **(H–I)** Time-dependent hydrogen bond numbers between COX-2 and C41 (H) or C136 (I). **(J–K)** MM/GBSA binding free energy decomposition for C41 (J) and C136 (K). **(L–M)** Molecular docking visualization of C41 (L) and C136 (M) with COX-2; magnified views on the right display hydrogen bond interactions with key residues. **(N)** Visual representation of the binding pockets for C41, C136, and JMS on the COX-2 protein. **(O)** Effects of C41 and C136 on the flexibility and volume of JMS-defined pocket. **(P)** Residue-resolved Cα RMSF of the 18 JMS-defined pocket residues. **(Q)** Orthosteric docking scores (left) and remote-to-orthosteric dynamic coupling relative to the matched APO baseline (right). *p < 0.05 *versus* the DMSO group, ^#^ *p* < 0.05 *versus* the LPS group.

##### 3.4.2.2 High-affinity binding modes revealed by molecular dynamics simulation

To elucidate the binding characteristics of these active components with COX-2, 150 ns molecular dynamics simulations were performed. Results indicated that both ligands stably bound to the COX-2 active pocket: **C41** exhibited transient conformational fluctuations between 60 and 70 ns, with root-mean-square deviation (RMSD) maintained below 0.5 nm for the remaining duration (Figure 10D); **C136** reached binding equilibrium after 120 ns (Figure 10E).

Further binding mode analysis revealed that **C41** formed persistent hydrogen bond interactions with key residues Glu-141, Asn-145, Leu-146, Ser-147, and Glu-236 in the COX-2 active site (Figure 10L); whereas **C136** primarily formed hydrogen bonds with residues Lys-212, Glu-237, Gln-271, and Glu-291 (Figure 10M). The binding free energies calculated by MM/GBSA were −29.05 kcal·mol^-1^ for **C41** and -28.34 kcal·mol^-1^ for **C136** (Figures 10J,K), indicating high affinity driven primarily by van der Waals forces, electrostatic interactions, and nonpolar solvation effects. These data corroborate the *in vitro* COX-2 inhibitory activity results, supporting that **C41** and **C136** directly occupy the COX-2 active site with high affinity.

Binding pocket analysis predicted that the COX-2 cavity occupied by **C41** has a depth of approximately 18.5 Å, solvent-accessible surface area of 960 Å^2^, and internal volume of 926 Å^3^, with its rim and floor providing 19 hydrogen bond acceptors and 22 hydrogen bond donors, forming extensive complementary interactions with the multi-hydroxyl structure of **C41** (Supplementary Figure S5A). **C136** binds to the same active pocket, which provides 20 hydrogen bond acceptors and 11 donors; although with fewer hydrogen bond donors, its enlarged nonpolar surface can provide compensatory strong van der Waals interactions for this triterpenoid saponin (Supplementary Figure S5B). Thus, both predicted pockets possess the depth, surface area, and hydrogen bond networks required for high-affinity, shape-selective recognition of their respective ligands.

COX-2 has a canonical catalytic pocket (Pocket 1, where JMS binds); however, our results showed that C41 and C136 did not bind at the canonical pocket position (Figure 10N). To further investigate whether their binding sites exert allosteric regulation on the canonical catalytic pocket, we performed an allosteric analysis based on the APO molecular dynamics trajectories. According to the predicted results, relative to the APO state, both C41 and C136 reduced the mean pocket RMSF (−0.108 Å and −0.176 Å, respectively) and free volume (−39.7 Å^3^ and −20.7 Å^3^, respectively) of JMS-defined pocket. C41 shortened the Arg120–Tyr355 distance by 1.02 Å, whereas C136 shortened it by 0.48 Å; both increased the Tyr355–Ser530 distance by approximately 0.95 Å (Figure 10O).

The canonical COX-2 channel contains key residues including Arg120, Tyr355, Tyr385, Trp387, Phe518, Met522, and Ser530. Residue-level analysis showed particularly strong C136-associated reductions at Tyr355, Ala527, Ser530, Leu352, and Ser353. C41 also stabilized Ala527 and Ser530, while Val116, Arg120, and Leu384 showed modest increases, indicating retained local heterogeneity near the entrance (Figure 10P). The remote-to-orthosteric dynamic coupling analysis showed that the docking scores of C41 and C136 in JMS-defined pocket were −6.117 and 17.024 kcal/mol, respectively, while the positive control JMS scored −9.054 kcal/mol. This also indicates that both compounds have relatively low binding affinity for the known canonical catalytic pocket and cannot directly bind to the canonical pocket position (Figure 10Q). In addition, analysis of the coupling between the side pocket and the orthosteric pocket showed that C41 binding increased the mean absolute correlation between the remote site and the canonical pocket, with an increment approximately 4.6-fold greater than that observed for C136 (Figure 10Q).

## 4 Discussion

MASLD represents a global health challenge with limited pharmacotherapy options (Younossi et al., 2025). Using a physiologically relevant HFHFD model, this study provides a comprehensive mechanistic evaluation of the traditional formula MLN. Building upon previous work in a methionine-choline-deficient (MCD) diet model (Jia et al., 2022), an integrated “exposure-omics-validation” strategy was employed. This approach not only confirmed PPARα activation as a conserved mechanism but also substantially expanded the mechanistic understanding by linking it to specific lipidomic reprogramming and identifying a novel, complementary anti-inflammatory axis through direct COX-2 inhibition.

### 4.1 Identification of bioactive constituents and an overview of the integrated mechanism

A pivotal challenge in TCM research is to distinguish the multitude of compounds present in an extract from those that actually reach the target sites to exert biological effects (Tian et al., 2025). In this study, we first established a comprehensive chemical profile of MLN, identifying 143 constituents. Crucially, we then tracked their *in vivo* fate, detecting 48 serum- and 92 liver-enriched prototypes. This step of “bioavailability filtering” serves as a rigorous cornerstone of the serum pharmacochemistry strategy, effectively bridging the gap between the chemical complexity of the formula and its *in vivo* pharmacological reality (Wang et al., 2011). This approach fundamentally shifts the focus of mechanistic investigation from the numerous compounds present in the formula crude extract to the biologically relevant subset that is actually absorbed and enriched in the target organ. Consequently, it establishes a more credible and physiologically translatable link between the chemistry of MLN and its observed therapeutic efficacy. Notably, the MLNE (MLN excipients) group, which contained all non-active formulation components at concentrations matching those in the high-dose MLN group, did not produce significant improvements in any of the key pharmacodynamic parameters compared to the HFHFD group. This negative control further confirms that the observed therapeutic benefits are specifically attributable to the absorbed bioactive constituents of MLN rather than to the excipient vehicle, thereby reinforcing the specificity of the active ingredients identified through our serum pharmacochemistry screening.

### 4.2 PPARα-mediated restoration of lipid homeostasis and specific phospholipid remodeling

The integrated lipidomics analysis revealed that MLN elicits a dual corrective effect on the disturbed hepatic lipidome, significantly reducing accumulated TAGs while concurrently restoring depleted membrane phospholipids. This reversal of the pathological “high-TAG, low-PL” profile underscores MLN’s capacity to correct a fundamental lipid imbalance in MASLD.

Activation of PPARα pathway, a recognized strategy to enhance fatty acid oxidation, was identified as a central mechanism. MLN treatment robustly upregulated PPARα and its downstream *β*-oxidation genes. Crucially, this activity was deconvoluted to specific liver-enriched constituents. A suite of chemically diverse compounds, including phenolic acids (chlorogenic acid), iridoid glycosides (harpagide, macranthoidin B), and phenylethanoid glycosides (angoroside C), were validated as direct PPARα agonists. This discovery of multiple PPARα agonists within MLN provides a tangible molecular basis for its consistent anti-steatotic efficacy across different disease models.

Beyond gross lipid clearance, systems-level lipidomics revealed that PPARα activation is coupled with a precise remodeling of the hepatic phospholipidome. MLN specifically restored levels of PC (16:0/20:4) and lyso-phosphatidylcholine (20:4), species enriched in ARA. This “acyl-chain-specific” reprogramming suggests that MLN-driven PPARα activation channels metabolic flux toward the incorporation of long-chain polyunsaturated fatty acids into membranes, a process critical for hepatocellular integrity (Tian et al., 2024). This nuanced mechanism links agonist-induced transcriptional activation to a defined functional lipidomic outcome, advancing from mere pathway reporting.

The critical role of oxidative stress and lipid peroxidation in the pathogenesis of metabolic liver diseases has been systematically highlighted in a recent bibliometric analysis, which comprehensively mapped research trends in this area over the past 2 decades (Yao et al., 2024). Furthermore, a recent study demonstrated that flavonoid-rich mulberry leaf extract improves lipid metabolism, antioxidant capacity, and gut microbiota in high-fat diet-induced obesity through regulation of FGF21 and SOCS2 (Liu et al., 2024), providing a mechanistic precedent for how natural product-derived compounds can exert beneficial metabolic effects through pathways that may complement the PPARα-mediated mechanisms identified in our study.

### 4.3 Selective inhibition of the COX-2 axis by novel natural products

The beneficial increase in ARA-containing phospholipids also positions ARA as a substrate at the metabolism-inflammation interface. Liberated ARA can be metabolized via COX-2 to generate pro-inflammatory prostaglandins, a key driver in steatohepatitis progression (Tilg and Moschen, 2010). MLN treatment significantly suppressed this axis, reducing prostaglandin levels and hepatic COX-2 expression.

The major advance herein is the deconvolution of this effect to specific molecules. From the liver-enriched prototypes, the iridoid glycoside secologanoside and the triterpenoid saponin chikusetsusaponin IVa were identified and validated as direct, novel inhibitors of COX-2 enzyme activity *in vitro*. The translational relevance of these findings was further supported by cellular experiments, where both compounds effectively suppressed LPS-induced PGE_2_ production in RAW264.7 macrophages, confirming that their COX-2 inhibitory activity is preserved in a physiologically relevant cellular environment. Both C41 and C136 inhibit COX-2 activity through allosteric regulation, exerting side-pocket-mediated modulation by altering the flexibility and rigidity of the catalytic pocket.

This moves the understanding beyond pathway association to a definitive, reductionist pharmacological principle. The targeted COX-2 inhibition, coupled with the observed lipidomic shift favoring ARA incorporation into membranes, suggests that MLN coordinately modulates ARA flux, promoting its retention in structural phospholipids while limiting its diversion into pro-inflammatory eicosanoids. This exemplifies how different compound classes within a formula can engage complementary targets to address interconnected pathologies.

### 4.4 Methodological and ethnopharmacological implications

This study delineates a systematic, exposure-driven research paradigm for the mechanistic deconstruction of complex botanical formulations. The workflow progresses sequentially: it begins by mapping *in vivo* compound exposure via serum pharmacochemistry, then captures system-wide phenotypic responses through integrated lipidomics, and finally validates direct molecular targets using reductionist bioassays. This approach establishes a causal evidence chain that credibly links the holistic efficacy of a TCM formula to specific, identifiable pharmacological principles. This integrated strategy moves beyond the limitations of purely descriptive “omics” profiling or predictive network pharmacology, offering a robust and translatable framework for elucidating the composition-activity relationships of other multi-herb preparations.

In the context of ethnopharmacology, a primary objective is to provide a scientific rationale for traditional therapeutic applications. The findings herein contribute significantly to an evidence-based understanding of MLN. The proposed dual-pathway mechanistic framework, which involves PPAR*α*-mediated restoration of lipid homeostasis and COX-2-targeted suppression of inflammation, provides a contemporary pharmacological interpretation for the formula’s traditional use. This use historically addressed disorders linked to “blood stasis” and liver dysfunction, conditions that align with the modern pathophysiology of MASLD. By pinpointing specific bioactive constituents, such as phenylethanoid glycosides and secologanoside, and their direct molecular targets, this work not only validates the empirical knowledge embedded in MLN’s traditional use but also supports its potential rational development and modernization as a targeted phytotherapeutic agent for metabolic liver disease.

### 4.5 Non-monotonic dose-response relationship: Observations and mechanistic considerations

An unexpected finding of this study was that the low dose of MLN (5.4 mL/kg) consistently exhibited superior efficacy compared to the high dose (10.8 mL/kg) across multiple pharmacodynamic parameters, including hepatic lipid content, serum TC content, and serum ALT activity (Figures 3, 4). This non-monotonic dose-response relationship, while counterintuitive from a conventional pharmacological perspective, has been increasingly documented in natural product pharmacology, particularly for multi-component botanical extracts (Bondy, 2023; Chen et al., 2022).

Several interconnected mechanisms may account for this phenomenon. First, the hormesis phenomenon—characterized by biphasic dose-responses where low doses stimulate beneficial adaptive responses (e.g., Nrf2-mediated antioxidant defense) while high doses become inhibitory or less effective—has been well-described for various phytochemicals, including naringin and berberine (Calabrese et al., 2023; Yang et al., 2025), with similar biphasic dose-responses documented for plant flavonoids across diverse therapeutic contexts (Vazhappilly et al., 2025). Second, at higher concentrations, certain active compounds in MLN may approach saturation kinetics for key receptors such as PPARα, a well-validated core target of MLN (Jia et al., 2022; Jia et al., 2025), potentially leading to receptor desensitization or negative feedback regulation. Consistent with this possibility, our existing qPCR data (Figure 6) showed that the expression of several PPARα downstream targets (e.g., Cpt1a, Acadm) tended to be higher at the low dose than at the high dose, suggesting that higher concentrations may approach the upper limit of transcriptional activation. Third, as a multi-component botanical formulation, MLN contains diverse bioactive constituents whose pharmacokinetic interactions may become more complex and potentially counteractive at higher concentrations (Panossian, 2023). Nevertheless, we acknowledge that the mechanistic basis of this non-monotonic response remains incompletely defined. Future studies employing dose-titration designs, pharmacokinetic-pharmacodynamic modeling, and systems pharmacology approaches will be necessary to fully elucidate the complex interplay among the multiple active constituents of MLN across different dose levels.

### 4.6 Limitations and future perspectives

Several limitations of the screening strategy should be acknowledged. The initial phenotypic screening step—based on reduction of lipid accumulation in HepaRG cells—may not capture all compounds that directly target PPARα, particularly those that do not affect lipid accumulation *in vitro*. Conversely, compounds that reduce lipid accumulation through other pathways (e.g., AMPK activation, SREBP-1c inhibition, or ACC modulation) would proceed to the PPRE reporter assay but would be excluded at the PPARα confirmation stage, as they would not activate PPARα transcription. This two-stage approach was intentionally designed to prioritize compounds with both lipid-lowering efficacy and PPARα agonist activity, which are most likely to translate to therapeutic benefit in MASLD. Our primary goal was to deconvolute the active constituents responsible for the PPARα-mediated lipid-lowering effects of MLN, which have been firmly established in our prior work (Jia et al., 2022; Jia et al., 2025). However, we acknowledge that this screening cascade may overlook PPARα agonists that lack direct lipid-lowering effects, as well as non-PPARα lipid-lowering agents. Future studies employing unbiased transcriptomic or proteomic approaches will be needed to comprehensively map the full spectrum of MLN’s mechanisms of action.

Notwithstanding the insights provided, this study has limitations. The individual and synergistic contributions of the identified agonists and inhibitors to the overall *in vivo* efficacy warrant further pharmacokinetic-pharmacodynamic analysis. Validation in models that progress to advanced steatohepatitis with fibrosis is needed to define MLN’s therapeutic scope across the disease spectrum. The enzymatic regulators of the observed phospholipid remodeling and the translational relevance of the specific lipidomic signatures are important future research directions. Additionally, the discordance between profound hepatic lipidomic changes and more subtle serum alterations underscores the challenge in identifying translational biomarkers. Future clinical studies should investigate whether the specific phospholipid subspecies and oxylipins altered by MLN could serve as sensitive pharmacodynamic biomarkers for patient stratification and treatment monitoring.

## 5 Conclusion

In conclusion, this study demonstrates that the traditional formula MLN alleviates HFHFD-induced MASLD through an integrated dual-pathway mechanism involving PPARα-mediated lipid homeostasis restoration and direct COX-2 inhibition. By combining serum pharmacochemistry with systems lipidomics and target-based validation, we identified bioavailable constituents within MLN as novel PPARα agonists and COX-2 inhibitors, providing a molecular basis for its multi-target pharmacology. Notably, MLN evolved from the classic TCM formula Simiao Yong’an Decoction, originally indicated for heat-clearing and blood-activation in vasculopathy. Our findings extend its ethnopharmacological relevance by demonstrating, for the first time, its modulatory effects on hepatic lipid metabolism and COX-2-mediated inflammation, thereby supporting the rational repurposing of MLN as an evidence-based therapeutic strategy for early-stage MASLD. Collectively, these results bridge ethnopharmacological knowledge with contemporary metabolic disease management.

## Data Availability

The data presented in the study are deposited in the Science Data Bank (ScienceDB) repository, doi: https://doi.org/10.57760/sciencedb.0133x.

## Glossary

ABR: Achyranthis Bidentatae Radix
*Acadl*: Acyl-CoA dehydrogenase long chain
*Acadm*: Acyl-CoA dehydrogenase medium chain
*Acads*: Acyl-CoA dehydrogenase short chain
*Acadvl*: Acyl-CoA dehydrogenase very long chain
ALT: alanine aminotransferase
AST: aspartate aminotransferase
ARA: arachidonic acid
COX-2: cyclooxygenase-2
*Cpt1a*: carnitine palmitoyltrans-ferase 1A
CYP450: cytochrome p450
DAG: diglyceride
DC: Dendrobium Caulis
DHA: docosahexaenoic acid
DiHET: dihydroxyeicosatrienoic acid
EET: epoxyeicosatrienoic acid
EPA: eicosapentaenoic acid
FF: fenofibrate
GL: glycerolipid
GlcCer: glucosylceramide
GP: glycerophospholipid
HETE: hydroxyeicosatetraenoic acid
HFHFD: high-fat high-fructose high-cholesterol diet
HPLC: High performance liquid chromatography
LA: linoleic acid
LacCer: lactosylceramide
LC-MS/MS: liquid chromatography-mass spectrometry/mass spectrometry
LF: Lonicerae Flos
LJF: Lonicerae Japonicae Flos
LOX: lipoxygenase
LPC: lyso-phosphatidylcholine
LPE: lysophosphatidy-lethanolamine
LPG: lyso-phosphatidylglycerol
LPS: lyso-phosphatidylserine
MAG: monoglyceride
MASH: metabolic dysfunction-associated steatohepatitis
MASLD: metabolic dysfunction-associated steatotic liver disease
MLN: Mailuoning oral liquid
MLNE: MLN excipients
NCD: normal chow diet
PA: phosphatidic acid
PUFAs: poly-unsaturated fatty acids
PC: phosphatidylcholine
PE: phosphatidylethanolamine
**PGE**_**2**_: prostaglandin E_2_
**PGD**_**2**_: prostaglandin D_2_
PPARα: peroxisome proliferator-activated receptor α
PPRE: peroxisome proliferator response element
*Ptgs1*: prostaglandin-endoperoxide synthase 1
*Ptgs2*: prostaglandin-endoperoxide synthase 2
PUFA: polyunsaturated fatty acid
SR: Scrophulariae Radix
TAG: triglyceride
TCM: traditional Chinese medicine
TIC: total ion chromatogram
*Tnf*: Tumor necrosis factor
UHPLC-Q-Exactive Orbitrap HRMS: ultra-high-performance liquid chromatography coupled with Q-Exactive Orbitrap high-resolution mass spectrometry
