# Supplementary material for: Integrated chemical profiling and lipidomics reveal that Mailuoning oral liquid alleviates diet-induced MASLD via PPARα-mediated lipid homeostasis and COX-2 inhibition, with identification of potential active constituents
Source: Front Pharmacol. 2026 Oct 29;17:1939197. doi: 10.3389/fphar.2026.1939197 (PMC13755613; doi:10.3389/fphar.2026.1939197)
Supplement: Supplementary file 1 [file Supplementaryfile1.docx]

**Supporting information**

**Integrated chemical profiling and lipidomics reveal that Mailuoning oral liquid alleviates diet-Induced MASLD via PPARα-mediated lipid homeostasis and COX-2 inhibition, with identification of potential active constituents**

Sijia Wu^1,2†^, Wangya Jia^1†^, Xun Zhou^1^, Linyan Zhu^1^, Keke Wang^1,3^, Jian Li^4,5^, Lili Ji^1^*, Wei Liu^2^*and Hong Xu^1^*

^1^Institute of Chinese Materia Medica, Shanghai University of Traditional Chinese Medicine, The MOE Key Laboratory for Standardization of Chinese Medicines, Shanghai, China, ^2^Department of Pharmacy, The SATCM Third Grade Laboratory of Traditional Chinese Medicine Preparations, Key Laboratory of Liver and Kidney Diseases (Ministry of Education), Shanghai Key Laboratory of Traditional Chinese Clinical Medicine, Shuguang Hospital Affiliated to Shanghai University of Traditional Chinese Medicine, Shanghai, China, ^3^Institute of Chinese Medicinal Materials, Gansu Academy of Agricultural Sciences, lanzhou, Gansu, China,  ^4^Department of Pharmaceutics, China Pharmaceutical University, Nanjing, Jiangsu, China, ^5^Technology Center of Jinling Pharmaceutical Co., Ltd., Nanjing, Jiangsu, China

^†^These authors share first authorship

*CORRESPONDENCE: Hong Xu (xuhongshutcm@163.com),

Wei Liu ([lwhzayl@163.com](mailto:lwhzayl@163.com))

Lili Ji ([jilili@shutcm.edu.cn](mailto:jilili@shutcm.edu.cn)).

**Supplementary table legends**

**Table S1.** Mobile phase elution conditions for characterizing the chemical composition of MLN.

**Table S2.** Mass spectral parameters characterizing the chemical composition of MLN.

**Table S3.** List of primers for real-time PCR.

**Table S4.** Mobile phase elution conditions for non-targeted lipidomics assays.

**Table S5.** MS detection conditions and detection status of lipids in rat serum and liver.

**Table S6.** Mobile phase elution conditions for PUFAs.

**Table S7.** MRM transitions of PUFAs on LC-MS/MS.

**Table S8.** Compounds identified from MLN, serum and liver using UHPLC-Q-Exactive Orbitrap MS.

**Supplementary figure legends**

**Figure S1.** HPLC chromatogram of BS, RCs, and MLN.

**Figure S2.** Total ion chromatograms (TICs) of individual herbal ingredients in MLN. **A1** Positive ion mode of *Achyranthis bidentatae* radix. **A2** Negative ion mode of Achyranthis bidentatae radix. **B1** Positive ion mode of *Scrophulariae* radix. **B2** Negative ion mode of *Scrophulariae* radix. **C1** Positive ion mode of *Dendrobium* caulis. **C2** Negative ion mode of *Dendrobium* caulis. **D1** Positive ion mode of *Lonicerae japonicae* flos. **D2** Negative ion mode of *Lonicerae japonicae* flos. **E1** Positive ion mode of *Lonicerae* flos. **E2** Negative ion mode of *Lonicerae* flos.

**Figure S3.** Changes in feed intake, body weight, and blood glucose levels in rats under the HFHFD model. **A** Feed intake of rats (*n* = 6). **B** Body weight of rats throughout the experiment (*n* = 6). **C** Time course of blood glucose levels analysis after oral glucose administration (*n* = 6).

**Figure S4.** Screening of the lipid-lowering activity of compounds in Mailuoning Oral Liquid (MLN). **A** Information on the compounds. **B** Representative BODIPY fluorescence images. **C** Quantitative analysis of the mean fluorescence intensity (MFI); ART, atorvastatin, was used as the positive control. Data are presented as the mean ± SEM (*n* = 3). ***p* < 0.01, ****p* < 0.001 vs BSA group; #*p* < 0.05, ##*p* < 0.01, ###*p* < 0.001 vs NEFA group.

**Figure S5.** Visualization of the predicted binding pockets of C41 and C136 with COX-2. **A** Predicted binding pocket of C41 with COX-2. **B** Predicted binding pocket of C136 with COX-2.

**Figure S6.** Uncropped images of the original western blots in figure 6 (*n*=3).

**Table S1.** Mobile phase elution conditions for characterizing the chemical composition of MLN.

| Time (min) | Mobile Phase B |
| --- | --- |
| 0–2.0 | 4% |
| 2.0–6.0 | 4–12% |
| 6.0–38.0 | 12–70% |
| 38.0–38.5 | 70% |
| 38.5–39.0 | 70–95% |
| 39.0–43.0 | 95% |
| 43.0–43.1 | 95–4% |
| 43.1–45.0 | 4% |

**Table S2.** Mass spectral parameters characterizing the chemical composition of MLN.

| MS Parameter | Positive Ion Mode | Negative Ion Mode |
| --- | --- | --- |
| Full Scan Resolution | 70,000 FWHM | |
| MS2 Resolution | 17,500 FWHM | |
| Mass Range | m/z 100–1500 | |
| Sheath Gas (Nitrogen) Flow Rate | 35 arb | 40 arb |
| Auxiliary Gas (Nitrogen) Flow Rate | 8 arb | 15 arb |
| Capillary Temperature | 320 °C | |
| Spray Voltage | 3.8 kV | 3.0 kV |
| Stepped Normalized Collision Energy | 10, 20, 40 eV | |

**Table S3.** List of primers for Real-time PCR.

| Gene | Species | Sequence | |
| --- | --- | --- | --- |
|  |  | Forward (5′→3′) | Reverse (5′→3′) |
| *Ppara* | Rat | ACGATGCTGTCCTCCTTGATGAAC | ATGATGTCGCAGAATGGCTTCCTC |
| *Cpt1a* | Rat | CAAGTCAACGGCAGAGCAGAGG | TCATGGCGAGGCGGTACAGG |
| *Acads* | Rat | GCGTACTTCACGGCACAAT | GGGATGGGCTTCAAGATAG |
| *Acadm* | Rat | CGCAATAGAGGCAAAGTA | AGAAATGGCGATGAAAGT |
| *Acadl* | Rat | TGAACTCACAGGCAGAAA | GGGCAAGAAGCTACATAA |
| *Acadvl* | Rat | ACTGCGACTCAACTCTGG | GACTTGGTAATGCCCTAAA |
| *Ptgs1* | Rat | AGCACATTCGGTGGTGATGT | GGGTAATCTGGCACACGGAA |
| *Ptgs2* | Rat | CTCAGCCATGCAGCAAATCC | GGGTGGGCTTCAGCAGTAAT |
| *Actin* | Rat | GATCAAGATCATTGCTCCTCCTG | AGGGTGTAAAACGCAGCTCA |

**Table S4.** Mobile phase elution conditions for non-targeted lipidomics assays.

| Time (min) | Mobile Phase B |
| --- | --- |
| 0–0.5 | 20% |
| 0.5–1.5 | 20%–40% |
| 1.5–3.0 | 40%–60% |
| 3.0–13.0 | 60%–98% |
| 13.0–13.1 | 98%–20% |
| 13.1–17.0 | 20% |

**Table S5.** MS detection conditions and detection status of lipids in rat serum and liver.

| No. | Name | MRM (m/z) | DP | CE | serum | liver |
| --- | --- | --- | --- | --- | --- | --- |
| 1 | PC(14:0/14:0) | 736.5→227.2 | -80 | -50 |  |  |
| 2 | PC(14:0/16:0) | 764.5→227.2 | -80 | -50 |  | + |
| 3 | PC(14:0/16:1) | 762.5→253.2 | -80 | -50 |  | + |
| 4 | PC(14:0/18:0) | 792.6→227.2 | -80 | -50 |  |  |
| 5 | PC(14:0/18:1) | 790.6→281.2 | -80 | -50 |  | + |
| 6 | PC(14:0/18:2) | 788.5→279.2 | -80 | -50 |  | + |
| 7 | PC(14:0/18:3) | 786.5→277.2 | -80 | -50 |  |  |
| 8 | PC(14:0/20:1) | 818.6→309.3 | -80 | -50 |  |  |
| 9 | PC(14:0/20:2) | 816.6→307.3 | -80 | -50 |  |  |
| 10 | PC(14:0/20:3) | 814.6→305.2 | -80 | -50 |  |  |
| 11 | PC(14:0/20:4) | 812.5→303.2 | -80 | -50 |  | + |
| 12 | PC(14:0/20:5) | 810.5→301.2 | -80 | -50 |  |  |
| 13 | PC(14:0/22:4) | 840.6→331.3 | -80 | -50 |  |  |
| 14 | PC(14:0/22:5) | 838.6→329.2 | -80 | -50 |  |  |
| 15 | PC(14:0/22:6) | 836.5→327.2 | -80 | -50 |  |  |
| 16 | PC(16:0/16:0) | 792.6→255.2 | -80 | -50 | + | + |
| 17 | PC(16:0/16:1) | 790.6→253.2 | -80 | -50 | + | + |
| 18 | PC(16:0/18:0) | 820.6→283.3 | -80 | -50 |  |  |
| 19 | PC(16:0/18:1) | 818.6→281.2 | -80 | -50 | + | + |
| 20 | PC(16:0/18:2) | 816.6→279.2 | -80 | -50 | + | + |
| 21 | PC(16:0/18:3) | 814.6→277.2 | -80 | -50 |  | + |
| 22 | PC(16:0/20:1) | 846.6→309.3 | -80 | -50 |  |  |
| 23 | PC(16:0/20:2) | 844.6→307.3 | -80 | -50 |  |  |
| 24 | PC(16:0/20:3) | 842.6→305.2 | -80 | -50 | + | + |
| 25 | PC(16:0/20:4) | 840.6→303.2 | -80 | -50 | + | + |
| 26 | PC(16:0/20:5) | 838.6→301.2 | -80 | -50 |  | + |
| 27 | PC(16:0/22:4) | 868.6→331.3 | -80 | -50 |  |  |
| 28 | PC(16:0/22:5) | 866.6→329.2 | -80 | -50 |  |  |
| 29 | PC(16:0/22:6) | 864.6→327.2 | -80 | -50 |  | + |
| 30 | PC(16:1/16:1) | 788.6→253.2 | -80 | -50 |  | + |
| 31 | PC(16:1/18:0) | 818.6→253.2 | -80 | -50 |  | + |
| 32 | PC(16:1/18:1) | 816.6→281.2 | -80 | -50 | + | + |
| 33 | PC(16:1/18:2) | 814.6→253.2 | -80 | -50 |  | + |
| 34 | PC(16:1/18:3) | 812.5→253.2 | -80 | -50 |  |  |
| 35 | PC(16:1/20:1) | 844.6→253.2 | -80 | -50 |  |  |
| 36 | PC(16:1/20:2) | 842.6→253.2 | -80 | -50 |  |  |
| 37 | PC(16:1/20:3) | 840.6→253.2 | -80 | -50 |  |  |
| 38 | PC(16:1/20:4) | 838.6→253.2 | -80 | -50 |  | + |
| 39 | PC(16:1/20:5) | 836.5→253.2 | -80 | -50 |  |  |
| 40 | PC(16:1/22:4) | 866.6→253.2 | -80 | -50 |  |  |
| 41 | PC(16:1/22:5) | 864.6→253.2 | -80 | -50 |  |  |
| 42 | PC(16:1/22:6) | 862.6→253.2 | -80 | -50 |  |  |
| 43 | PC(18:0/18:0) | 848.6→283.3 | -80 | -50 |  | + |
| 44 | PC(18:0/18:1) | 846.6→281.2 | -80 | -50 | + | + |
| 45 | PC(18:0/18:2) | 844.6→279.2 | -80 | -50 | + | + |
| 46 | PC(18:0/18:3) | 842.6→277.2 | -80 | -50 |  | + |
| 47 | PC(18:0/20:1) | 874.7→309.3 | -80 | -50 |  |  |
| 48 | PC(18:0/20:2) | 872.6→307.3 | -80 | -50 |  |  |
| 49 | PC(18:0/20:3) | 870.6→305.2 | -80 | -50 | + | + |
| 50 | PC(18:0/20:4) | 868.6→303.2 | -80 | -50 | + | + |
| 51 | PC(18:0/20:5) | 866.6→301.2 | -80 | -50 |  |  |
| 52 | PC(18:0/22:4) | 896.6→331.3 | -80 | -50 |  |  |
| 53 | PC(18:0/22:5) | 894.6→329.2 | -80 | -50 |  |  |
| 54 | PC(18:0/22:6) | 892.6→327.2 | -80 | -50 |  |  |
| 55 | PC(18:1/18:1) | 844.6→281.2 | -80 | -50 | + | + |
| 56 | PC(18:1/18:2) | 842.6→279.2 | -80 | -50 | + | + |

| 57 | PC(18:1/18:3) | 840.6→277.2 | -80 | -50 |  |  |
| --- | --- | --- | --- | --- | --- | --- |
| 58 | PC(18:1/20:0) | 874.7→281.2 | -80 | -50 |  |  |
| 59 | PC(18:1/20:1) | 872.6→309.3 | -80 | -50 |  |  |
| 60 | PC(18:1/20:2) | 870.6→307.3 | -80 | -50 |  |  |
| 61 | PC(18:1/20:3) | 868.6→305.2 | -80 | -50 | + | + |
| 62 | PC(18:1/20:4) | 866.6→303.2 | -80 | -50 | + | + |
| 63 | PC(18:1/20:5) | 864.6→301.2 | -80 | -50 |  |  |
| 64 | PC(18:1/22:4) | 894.6→331.3 | -80 | -50 |  |  |
| 65 | PC(18:1/22:5) | 892.6→329.2 | -80 | -50 |  |  |
| 66 | PC(18:1/22:6) | 890.6→327.2 | -80 | -50 |  |  |
| 67 | PC(18:2/18:2) | 840.6→279.2 | -80 | -50 | + | + |
| 68 | PC(18:2/18:3) | 838.6→277.2 | -80 | -50 |  |  |
| 69 | PC(18:2/20:0) | 872.6→311.3 | -80 | -50 |  |  |
| 70 | PC(18:2/20:1) | 870.6→309.3 | -80 | -50 |  |  |
| 71 | PC(18:2/20:2) | 868.6→307.3 | -80 | -50 |  |  |
| 72 | PC(18:2/20:3) | 866.6→305.2 | -80 | -50 |  |  |
| 73 | PC(18:2/20:4) | 864.6→303.2 | -80 | -50 |  | + |
| 74 | PC(18:2/20:5) | 862.6→301.2 | -80 | -50 |  |  |
| 75 | PC(18:2/22:4) | 892.6→331.3 | -80 | -50 |  |  |
| 76 | PC(18:2/22:5) | 890.6→329.2 | -80 | -50 |  |  |
| 77 | PC(18:2/22:6) | 888.6→327.2 | -80 | -50 |  |  |
| 78 | PC(18:3/18:3) | 836.5→277.2 | -80 | -50 |  |  |
| 79 | PC(18:3/20:1) | 868.6→277.2 | -80 | -50 |  |  |
| 80 | PC(18:3/20:2) | 866.6→277.2 | -80 | -50 |  |  |
| 81 | PC(18:3/20:3) | 864.6→277.2 | -80 | -50 |  |  |
| 82 | PC(18:3/20:4) | 862.6→277.2 | -80 | -50 |  |  |
| 83 | PC(18:3/20:5) | 860.5→277.2 | -80 | -50 |  |  |
| 84 | PC(20:0/20:2) | 900.7→307.3 | -80 | -50 |  |  |
| 85 | PC(20:0/20:3) | 898.7→305.2 | -80 | -50 |  |  |
| 86 | PC(20:0/20:4) | 896.6→303.2 | -80 | -50 |  |  |
| 87 | PC(20:0/20:5) | 894.6→301.2 | -80 | -50 |  |  |
| 88 | PC(20:0/22:4) | 924.7→331.3 | -80 | -50 |  |  |
| 89 | PC(20:0/22:5) | 922.7→329.2 | -80 | -50 |  |  |
| 90 | PC(20:0/22:6) | 920.6→327.2 | -80 | -50 |  |  |
| 91 | PC(20:1/20:1) | 900.7→309.3 | -80 | -50 |  |  |
| 92 | PC(20:1/20:2) | 898.7→307.3 | -80 | -50 |  |  |
| 93 | PC(20:1/20:3) | 896.6→305.2 | -80 | -50 |  |  |
| 94 | PC(20:1/20:4) | 894.6→303.2 | -80 | -50 |  |  |
| 95 | PC(20:1/20:5) | 892.6→301.2 | -80 | -50 |  |  |
| 96 | PC(20:1/22:4) | 922.7→331.3 | -80 | -50 |  |  |
| 97 | PC(20:1/22:5) | 920.6→329.3 | -80 | -50 |  |  |
| 98 | PC(20:1/22:6) | 918.6→327.2 | -80 | -50 |  |  |
| 99 | PC(20:2/20:2) | 896.6→307.3 | -80 | -50 |  |  |
| 100 | PC(20:2/20:3) | 894.6→305.2 | -80 | -50 |  |  |
| 101 | PC(20:2/20:4) | 892.6→303.2 | -80 | -50 |  |  |
| 102 | PC(20:2/20:5) | 890.6→301.2 | -80 | -50 |  |  |
| 103 | PC(20:2/22:5) | 918.6→329.2 | -80 | -50 |  |  |
| 104 | PC(20:2/22:6) | 916.6→327.2 | -80 | -50 |  |  |
| 105 | PC(20:3/20:3) | 892.6→305.2 | -80 | -50 |  |  |
| 106 | PC(20:3/20:4) | 890.6→303.2 | -80 | -50 |  |  |
| 107 | PC(20:3/20:5) | 888.6→301.2 | -80 | -50 |  |  |
| 108 | PC(20:4/20:4) | 888.6→303.2 | -80 | -50 |  |  |
| 109 | PC(20:4/20:5) | 886.6→305.2 | -80 | -50 |  |  |
| 110 | PC(20:4/22:4) | 916.6→331.3 | -80 | -50 |  |  |
| 111 | PC(20:4/22:5) | 914.6→329.2 | -80 | -50 |  |  |
| 112 | PC(20:4/22:6) | 912.6→327.2 | -80 | -50 |  |  |
| 113 | PC(20:5/20:5) | 884.5→301.2 | -80 | -50 |  |  |
| 114 | PC(20:5/22:4) | 914.6→331.3 | -80 | -50 |  |  |
| 115 | PC(20:5/22:5) | 912.6→329.2 | -80 | -50 |  |  |
| 116 | PC(20:5/22:6) | 910.6→327.2 | -80 | -50 |  |  |
| 117 | PC(22:5/22:6) | 938.6→327.2 | -80 | -50 |  |  |
| 118 | PC(22:6/22:6) | 936.6→327.2 | -80 | -50 |  |  |
| 119 | PE(14:0/18:1) | 688.5→281.2 | -80 | -50 |  | + |
| 120 | PE(14:0/18:2) | 686.5→279.2 | -80 | -50 |  | + |
| 121 | PE(14:0/20:4) | 710.5→303.2 | -80 | -50 |  | + |
| 122 | PE(14:0/22:4) | 738.5→331.3 | -80 | -50 |  |  |
| 123 | PE(14:0/22:5) | 736.5→329.2 | -80 | -50 |  |  |
| 124 | PE(14:0/22:6) | 734.5→327.2 | -80 | -50 |  |  |
| 125 | PE(16:0/16:0) | 690.5→255.2 | -80 | -50 | + | + |
| 126 | PE(16:0/16:1) | 688.5→253.2 | -80 | -50 | + | + |
| 127 | PE(16:0/18:1) | 716.5→281.2 | -80 | -50 | + | + |
| 128 | PE(16:0/18:2) | 714.5→279.2 | -80 | -50 | + | + |
| 129 | PE(16:0/18:3) | 712.5→277.2 | -80 | -50 |  | + |
| 130 | PE(16:0/20:1) | 744.6→309.3 | -80 | -50 |  |  |
| 131 | PE(16:0/20:2) | 742.5→307.3 | -80 | -50 |  | + |
| 132 | PE(16:0/20:3) | 740.5→305.2 | -80 | -50 |  | + |
| 133 | PE(16:0/20:4) | 738.5→303.2 | -80 | -50 | + | + |
| 134 | PE(16:0/20:5) | 736.5→301.2 | -80 | -50 |  | + |
| 135 | PE(16:0/22:4) | 766.5→331.3 | -80 | -50 |  | + |
| 136 | PE(16:0/22:5) | 764.5→329.2 | -80 | -50 |  | + |
| 137 | PE(16:0/22:6) | 762.5→327.2 | -80 | -50 |  | + |
| 138 | PE(18:0/18:0) | 746.6→283.3 | -80 | -50 | + |  |
| 139 | PE(18:0/18:1) | 744.6→281.2 | -80 | -50 | + | + |
| 140 | PE(18:0/18:2) | 742.5→279.2 | -80 | -50 | + | + |
| 141 | PE(18:0/18:3) | 740.5→277.2 | -80 | -50 |  | + |
| 142 | PE(18:0/20:1) | 772.6→309.3 | -80 | -50 |  |  |
| 143 | PE(18:0/20:2) | 770.6→307.3 | -80 | -50 |  | + |
| 144 | PE(18:0/20:3) | 768.6→305.2 | -80 | -50 | + | + |
| 145 | PE(18:0/20:4) | 766.5→303.2 | -80 | -50 | + | + |
| 146 | PE(18:0/20:5) | 764.5→301.2 | -80 | -50 |  | + |
| 147 | PE(18:0/22:4) | 794.6→331.3 | -80 | -50 |  | + |
| 148 | PE(18:0/22:5) | 792.6→329.2 | -80 | -50 |  | + |
| 149 | PE(18:0/22:6) | 790.5→327.2 | -80 | -50 | + | + |
| 150 | PE(18:1/18:1) | 742.5→281.2 | -80 | -50 | + | + |
| 151 | PE(18:1/18:2) | 740.5→279.2 | -80 | -50 | + | + |
| 152 | PE(18:1/18:3) | 738.5→277.2 | -80 | -50 |  | + |
| 153 | PE(18:1/20:0) | 772.6→311.2 | -80 | -50 |  |  |
| 154 | PE(18:1/20:1) | 770.6→309.3 | -80 | -50 |  | + |
| 155 | PE(18:1/20:2) | 768.6→307.3 | -80 | -50 |  | + |
| 156 | PE(18:1/20:3) | 766.5→305.2 | -80 | -50 |  | + |
| 157 | PE(18:1/20:4) | 764.5→303.2 | -80 | -50 |  | + |
| 158 | PE(18:1/20:5) | 762.5→301.2 | -80 | -50 |  | + |
| 159 | PE(18:1/22:4) | 792.6→331.3 | -80 | -50 |  | + |
| 160 | PE(18:1/22:5) | 790.5→329.2 | -80 | -50 |  | + |
| 161 | PE(18:1/22:6) | 788.5→327.2 | -80 | -50 |  | + |
| 162 | PE(18:2/18:2) | 738.5→279.2 | -80 | -50 |  | + |
| 163 | PE(18:2/18:3) | 736.5→277.2 | -80 | -50 |  | + |
| 164 | PE(18:2/20:0) | 770.6→311.3 | -80 | -50 |  |  |
| 165 | PE(18:2/20:1) | 768.6→309.3 | -80 | -50 |  | + |
| 166 | PE(18:2/20:2) | 766.5→307.3 | -80 | -50 |  |  |
| 167 | PE(18:2/20:3) | 764.5→305.2 | -80 | -50 |  | + |
| 168 | PE(18:2/20:4) | 762.5→303.2 | -80 | -50 |  | + |
| 169 | PE(18:2/22:4) | 790.5→331.3 | -80 | -50 |  |  |
| 170 | PE(18:2/22:5) | 788.5→329.2 | -80 | -50 |  |  |
| 171 | PE(18:2/22:6) | 786.5→327.2 | -80 | -50 |  | + |
| 172 | PE(20:0/20:1) | 800.6→309.3 | -80 | -50 |  |  |
| 173 | PE(20:0/20:2) | 798.6→307.3 | -80 | -50 |  |  |
| 174 | PE(20:0/20:3) | 796.6→305.2 | -80 | -50 | + |  |
| 175 | PE(20:0/20:4) | 794.6→303.2 | -80 | -50 | + | + |
| 176 | PE(20:0/20:5) | 792.6→301.2 | -80 | -50 |  |  |
| 177 | PE(20:0/22:4) | 822.6→331.3 | -80 | -50 |  |  |
| 178 | PE(20:0/22:5) | 820.6→329.2 | -80 | -50 |  |  |
| 179 | PE(20:0/22:6) | 818.6→327.2 | -80 | -50 |  |  |
| 180 | PE(20:1/20:1) | 798.6→309.3 | -80 | -50 |  |  |
| 181 | PE(20:1/20:2) | 796.6→307.3 | -80 | -50 |  |  |
| 182 | PE(20:1/20:3) | 794.6→305.2 | -80 | -50 | + | + |
| 183 | PE(20:1/20:4) | 792.6→303.2 | -80 | -50 | + | + |
| 184 | PE(20:1/20:5) | 790.5→301.2 | -80 | -50 |  |  |
| 185 | PE(20:1/22:4) | 820.6→331.3 | -80 | -50 |  |  |
| 186 | PE(20:1/22:5) | 818.6→329.2 | -80 | -50 |  |  |
| 187 | PE(20:1/22:6) | 816.6→327.2 | -80 | -50 |  |  |
| 188 | PE(20:2/20:2) | 794.6→307.3 | -80 | -50 |  |  |
| 189 | PE(20:2/20:3) | 792.6→305.2 | -80 | -50 |  |  |
| 190 | PE(20:2/20:4) | 790.5→303.2 | -80 | -50 |  | + |
| 191 | PE(20:2/20:5) | 788.5→301.2 | -80 | -50 |  |  |
| 192 | PE(20:2/22:4) | 818.6→331.3 | -80 | -50 |  |  |
| 193 | PE(20:2/22:5) | 816.6→329.2 | -80 | -50 |  |  |
| 194 | PE(20:3/20:3) | 790.5→305.2 | -80 | -50 |  |  |
| 195 | PE(20:3/20:4) | 788.5→303.2 | -80 | -50 |  |  |
| 196 | PE(20:3/20:5) | 786.5→301.2 | -80 | -50 |  |  |
| 197 | PE(20:3/22:4) | 816.6→331.3 | -80 | -50 |  |  |
| 198 | PE(20:3/22:5) | 814.5→329.2 | -80 | -50 |  |  |
| 199 | PE(20:3/22:6) | 812.5→327.2 | -80 | -50 |  |  |
| 200 | PE(20:4/20:4) | 786.5→303.2 | -80 | -50 |  | + |
| 201 | PE(20:4/22:4) | 814.5→331.3 | -80 | -50 |  |  |
| 202 | PE(20:4/22:5) | 812.5→329.2 | -80 | -50 |  |  |
| 203 | PE(20:4/22:6) | 810.5→327.2 | -80 | -50 |  |  |
| 204 | PE(22:4/22:5) | 840.6→329.2 | -80 | -50 |  |  |
| 205 | PE(22:4/22:6) | 838.5→327.2 | -80 | -50 |  |  |
| 206 | PE(22:5/22:5) | 838.5→329.2 | -80 | -50 |  |  |
| 207 | PE(22:5/22:6) | 836.5→327.2 | -80 | -50 |  |  |
| 208 | PE(O-16:0/16:0) | 676.5→255.2 | -80 | -50 |  |  |
| 209 | PE(O-16:0/16:1) | 674.5→253.2 | -80 | -50 |  |  |
| 210 | PE(O-16:0/18:1) | 702.5→281.2 | -80 | -50 |  | + |
| 211 | PE(O-16:0/18:2) | 700.5→279.2 | -80 | -50 |  | + |
| 212 | PE(O-16:0/18:3) | 698.5→277.2 | -80 | -50 |  |  |
| 213 | PE(O-16:0/20:1) | 730.6→309.3 | -80 | -50 |  |  |
| 214 | PE(O-16:0/20:2) | 728.6→307.3 | -80 | -50 |  |  |
| 215 | PE(O-16:0/20:3) | 726.5→305.2 | -80 | -50 |  |  |
| 216 | PE(O-16:0/20:4) | 724.5→303.2 | -80 | -50 |  | + |
| 217 | PE(O-16:0/20:5) | 722.5→301.2 | -80 | -50 |  |  |
| 218 | PE(O-16:0/22:4) | 752.6→331.3 | -80 | -50 |  | + |
| 219 | PE(O-16:0/22:5) | 750.5→329.2 | -80 | -50 |  |  |
| 220 | PE(O-16:0/22:6) | 748.5→327.2 | -80 | -50 |  |  |
| 221 | PE(O-18:0/16:0) | 704.6→255.2 | -80 | -50 |  | + |
| 222 | PE(O-18:0/16:1) | 702.5→253.2 | -80 | -50 |  |  |
| 223 | PE(O-18:0/18:1) | 730.6→281.2 | -80 | -50 |  |  |
| 224 | PE(O-18:0/18:2) | 728.6→279.2 | -80 | -50 |  | + |
| 225 | PE(O-18:0/18:3) | 726.5→277.2 | -80 | -50 |  |  |
| 226 | PE(O-18:0/20:1) | 758.6→309.3 | -80 | -50 |  |  |
| 227 | PE(O-18:0/20:2) | 756.6→307.3 | -80 | -50 |  |  |
| 228 | PE(O-18:0/20:3) | 754.6→305.2 | -80 | -50 |  |  |
| 229 | PE(O-18:0/20:4) | 752.6→303.2 | -80 | -50 |  | + |
| 230 | PE(O-18:0/20:5) | 750.5→301.2 | -80 | -50 |  |  |
| 231 | PE(O-18:0/22:4) | 780.6→331.3 | -80 | -50 |  |  |
| 232 | PE(O-18:0/22:5) | 778.6→329.2 | -80 | -50 |  |  |
| 233 | PE(O-18:0/22:6) | 776.6→327.2 | -80 | -50 |  |  |
| 234 | PG(14:0/18:2) | 717.5→279.2 | -80 | -50 |  | + |
| 235 | PG(14:0/20:1) | 747.5→309.3 | -80 | -50 |  |  |
| 236 | PG(14:0/20:4) | 741.5→303.2 | -80 | -50 |  | + |
| 237 | PG(14:0/22:4) | 769.5→331.3 | -80 | -50 |  |  |
| 238 | PG(14:0/22:6) | 765.5→327.2 | -80 | -50 |  |  |
| 239 | PG(16:0/16:0) | 721.5→255.2 | -80 | -50 |  | + |
| 240 | PG(16:0/18:0) | 749.5→283.3 | -80 | -50 |  |  |
| 241 | PG(16:0/18:1) | 747.5→281.2 | -80 | -50 |  | + |
| 242 | PG(16:0/18:2) | 745.5→279.2 | -80 | -50 |  | + |
| 243 | PG(16:0/20:0) | 777.6→311.3 | -80 | -50 |  |  |
| 244 | PG(16:0/20:1) | 775.5→309.3 | -80 | -50 |  |  |
| 245 | PG(16:0/20:2) | 773.5→307.3 | -80 | -50 |  |  |
| 246 | PG(16:0/20:3) | 771.5→305.2 | -80 | -50 |  |  |
| 247 | PG(16:0/20:4) | 769.5→303.2 | -80 | -50 |  | + |
| 248 | PG(16:0/22:5) | 795.5→329.2 | -80 | -50 |  |  |
| 249 | PG(16:0/22:6) | 793.5→327.2 | -80 | -50 |  |  |
| 250 | PG(16:1/16:1) | 717.5→253.2 | -80 | -50 | + | + |
| 251 | PG(16:1/18:1) | 745.5→281.2 | -80 | -50 | + | + |
| 252 | PG(16:1/18:2) | 743.5→279.2 | -80 | -50 | + | + |
| 253 | PG(16:1/20:0) | 775.5→253.2 | -80 | -50 |  |  |
| 254 | PG(16:1/20:1) | 773.5→309.3 | -80 | -50 |  |  |
| 255 | PG(16:1/20:2) | 771.5→307.3 | -80 | -50 |  |  |
| 256 | PG(16:1/20:3) | 769.5→305.2 | -80 | -50 | + | + |
| 257 | PG(16:1/20:4) | 767.5→303.2 | -80 | -50 | + | + |
| 258 | PG(16:1/20:5) | 765.5→301.2 | -80 | -50 |  | + |
| 259 | PG(16:1/22:4) | 795.5→331.3 | -80 | -50 |  | + |
| 260 | PG(16:1/22:5) | 793.5→329.2 | -80 | -50 |  | + |
| 261 | PG(16:1/22:6) | 791.5→327.2 | -80 | -50 |  | + |
| 262 | PG(18:0/18:0) | 777.6→283.3 | -80 | -50 |  |  |
| 263 | PG(18:0/18:1) | 775.5→281.2 | -80 | -50 |  |  |
| 264 | PG(18:0/18:2) | 773.5→279.2 | -80 | -50 |  | + |
| 265 | PG(18:0/20:1) | 803.6→309.3 | -80 | -50 |  |  |
| 266 | PG(18:0/20:2) | 801.6→307.3 | -80 | -50 |  |  |
| 267 | PG(18:0/20:4) | 797.5→303.2 | -80 | -50 |  |  |
| 268 | PG(18:1/18:1) | 773.5→281.2 | -80 | -50 | + | + |
| 269 | PG(18:1/18:2) | 771.5→279.2 | -80 | -50 | + | + |
| 270 | PG(18:1/18:3) | 769.5→277.2 | -80 | -50 |  |  |
| 271 | PG(18:1/20:1) | 801.6→309.3 | -80 | -50 |  |  |
| 272 | PG(18:1/20:2) | 799.5→307.3 | -80 | -50 |  |  |
| 273 | PG(18:1/20:3) | 797.5→305.2 | -80 | -50 |  | + |
| 274 | PG(18:1/20:4) | 795.5→303.2 | -80 | -50 | + | + |
| 275 | PG(18:1/20:5) | 793.5→301.2 | -80 | -50 |  |  |
| 276 | PG(18:1/22:4) | 823.5→331.3 | -80 | -50 |  |  |
| 277 | PG(18:1/22:5) | 821.5→329.2 | -80 | -50 |  |  |
| 278 | PG(18:1/22:6) | 819.5→327.2 | -80 | -50 |  |  |
| 279 | PG(18:2/18:2) | 769.5→279.2 | -80 | -50 | + |  |
| 280 | PG(18:2/18:3) | 767.5→277.2 | -80 | -50 |  |  |
| 281 | PG(18:2/20:1) | 799.5→309.3 | -80 | -50 |  |  |
| 282 | PG(18:2/20:2) | 797.5→307.3 | -80 | -50 |  |  |
| 283 | PG(18:2/20:3) | 795.5→305.2 | -80 | -50 |  | + |
| 284 | PG(18:2/20:4) | 793.5→303.2 | -80 | -50 |  | + |
| 285 | PG(18:2/22:4) | 821.5→331.3 | -80 | -50 |  |  |
| 286 | PG(18:2/22:5) | 819.5→329.2 | -80 | -50 |  |  |
| 287 | PG(18:3/18:3) | 765.5→277.2 | -80 | -50 |  |  |
| 288 | PG(18:3/20:0) | 799.5→311.3 | -80 | -50 |  |  |
| 289 | PG(18:3/20:1) | 797.5→309.3 | -80 | -50 |  |  |
| 290 | PG(18:3/20:2) | 795.5→307.3 | -80 | -50 |  |  |
| 291 | PG(18:3/20:3) | 793.5→305.2 | -80 | -50 |  |  |
| 292 | PG(18:3/20:4) | 791.5→303.2 | -80 | -50 |  | + |
| 293 | PG(18:3/20:5) | 789.5→301.2 | -80 | -50 |  |  |
| 294 | PG(18:3/22:4) | 819.5→331.3 | -80 | -50 |  |  |
| 295 | PG(20:0/20:1) | 831.6→309.3 | -80 | -50 |  |  |
| 296 | PG(20:0/20:2) | 829.6→307.3 | -80 | -50 |  |  |
| 297 | PG(20:0/20:3) | 827.6→305.2 | -80 | -50 |  |  |
| 298 | PG(20:0/20:4) | 825.6→303.2 | -80 | -50 |  |  |
| 299 | PG(20:0/20:5) | 823.5→301.2 | -80 | -50 |  |  |
| 300 | PG(20:0/22:4) | 853.6→331.3 | -80 | -50 |  |  |
| 301 | PG(20:0/22:5) | 851.6→329.2 | -80 | -50 |  |  |
| 302 | PG(20:0/22:6) | 849.6→327.2 | -80 | -50 |  |  |
| 303 | PG(20:1/20:1) | 829.6→309.3 | -80 | -50 |  |  |
| 304 | PG(20:1/20:2) | 827.6→307.3 | -80 | -50 |  |  |
| 305 | PG(20:1/20:3) | 825.6→305.2 | -80 | -50 |  |  |
| 306 | PG(20:1/20:4) | 823.5→303.2 | -80 | -50 |  |  |
| 307 | PG(20:1/22:4) | 851.6→331.3 | -80 | -50 |  |  |
| 308 | PG(20:1/22:5) | 849.6→329.2 | -80 | -50 |  |  |
| 309 | PG(20:2/22:5) | 847.5→329.2 | -80 | -50 |  |  |
| 310 | PG(20:2/22:6) | 845.5→327.2 | -80 | -50 |  |  |
| 311 | PG(20:3/20:3) | 821.5→305.2 | -80 | -50 |  |  |
| 312 | PG(20:3/20:4) | 819.5→303.2 | -80 | -50 |  |  |
| 313 | PG(20:3/20:5) | 817.5→301.2 | -80 | -50 |  |  |
| 314 | PG(20:3/22:4) | 847.5→331.3 | -80 | -50 |  |  |
| 315 | PG(20:3/22:5) | 845.5→329.2 | -80 | -50 |  |  |
| 316 | PG(20:3/22:6) | 843.5→327.2 | -80 | -50 |  |  |
| 317 | PG(20:4/20:4) | 817.5→303.2 | -80 | -50 |  |  |
| 318 | PG(20:4/20:5) | 815.5→301.2 | -80 | -50 |  |  |
| 319 | PG(20:4/22:4) | 845.5→331.3 | -80 | -50 |  |  |
| 320 | PG(20:4/22:5) | 843.5→329.2 | -80 | -50 |  |  |
| 321 | PG(20:4/22:6) | 841.5→327.2 | -80 | -50 |  |  |
| 322 | PG(20:5/20:5) | 813.5→301.2 | -80 | -50 |  |  |
| 323 | PG(20:5/22:4) | 843.5→331.3 | -80 | -50 |  |  |
| 324 | PG(20:5/22:5) | 841.5→329.2 | -80 | -50 |  |  |
| 325 | PG(20:5/22:6) | 839.5→327.2 | -80 | -50 |  |  |
| 326 | PG(22:4/22:4) | 873.6→331.3 | -80 | -50 |  |  |
| 327 | PG(22:4/22:5) | 871.5→329.2 | -80 | -50 |  |  |
| 328 | PG(22:4/22:6) | 869.5→327.2 | -80 | -50 |  |  |
| 329 | PG(22:5/22:5) | 869.5→329.2 | -80 | -50 |  |  |
| 330 | PG(22:5/22:6) | 867.5→327.2 | -80 | -50 |  |  |
| 331 | PG(22:6/22:6) | 865.5→327.2 | -80 | -50 | + |  |
| 332 | PS(14:0/16:0) | 706.5→255.2 | -80 | -50 |  |  |
| 333 | PS(14:0/16:1) | 704.5→253.2 | -80 | -50 |  |  |
| 334 | PS(14:0/18:0) | 734.5→283.3 | -80 | -50 |  |  |
| 335 | PS(14:0/18:1) | 732.5→281.2 | -80 | -50 |  |  |
| 336 | PS(14:0/18:2) | 730.5→279.2 | -80 | -50 |  |  |
| 337 | PS(14:0/20:4) | 754.5→303.2 | -80 | -50 |  |  |
| 338 | PS(14:0/20:5) | 752.5→301.2 | -80 | -50 |  |  |
| 339 | PS(14:0/22:4) | 782.5→331.3 | -80 | -50 |  |  |
| 340 | PS(14:0/22:5) | 780.5→329.2 | -80 | -50 |  |  |
| 341 | PS(14:0/22:6) | 778.5→327.2 | -80 | -50 |  |  |
| 342 | PS(16:0/16:0) | 734.5→255.2 | -80 | -50 |  |  |
| 343 | PS(16:0/18:0) | 762.5→283.3 | -80 | -50 |  | + |
| 344 | PS(16:0/18:2) | 758.5→279.2 | -80 | -50 |  |  |
| 345 | PS(16:0/20:0) | 790.6→311.3 | -80 | -50 |  |  |
| 346 | PS(16:0/20:4) | 782.5→303.2 | -80 | -50 |  |  |
| 347 | PS(16:0/22:4) | 810.5→331.3 | -80 | -50 |  |  |
| 348 | PS(16:0/22:6) | 806.5→327.2 | -80 | -50 |  |  |
| 349 | PS(16:1/16:1) | 730.5→253.2 | -80 | -50 |  |  |
| 350 | PS(16:1/18:0) | 760.5→283.3 | -80 | -50 |  |  |
| 351 | PS(16:1/18:1) | 758.5→281.2 | -80 | -50 |  | + |
| 352 | PS(16:1/18:2) | 756.5→279.2 | -80 | -50 |  |  |
| 353 | PS(16:1/18:3) | 754.5→277.2 | -80 | -50 |  |  |
| 354 | PS(16:1/20:3) | 782.5→305.2 | -80 | -50 |  |  |
| 355 | PS(16:1/20:4) | 780.5→303.2 | -80 | -50 |  | + |
| 356 | PS(16:1/22:4) | 808.5→331.3 | -80 | -50 |  |  |
| 357 | PS(16:1/22:5) | 806.5→329.2 | -80 | -50 |  |  |
| 358 | PS(16:1/22:6) | 804.5→327.2 | -80 | -50 |  |  |
| 359 | PS(18:0/18:0) | 790.6→283.3 | -80 | -50 |  | + |
| 360 | PS(18:0/18:1) | 788.5→281.2 | -80 | -50 |  | + |
| 361 | PS(18:0/18:2) | 786.5→279.2 | -80 | -50 |  | + |
| 362 | PS(18:0/20:0) | 818.6→311.3 | -80 | -50 |  |  |
| 363 | PS(18:0/20:1) | 816.6→309.3 | -80 | -50 |  |  |
| 364 | PS(18:0/20:2) | 814.6→307.3 | -80 | -50 |  |  |
| 365 | PS(18:0/20:4) | 810.5→303.2 | -80 | -50 |  | + |
| 366 | PS(18:0/22:4) | 838.6→331.3 | -80 | -50 |  |  |
| 367 | PS(18:0/22:5) | 836.5→329.2 | -80 | -50 |  |  |
| 368 | PS(18:0/22:6) | 834.5→327.2 | -80 | -50 |  |  |
| 369 | PS(18:1/18:2) | 784.5→279.2 | -80 | -50 |  |  |
| 370 | PS(18:1/20:2) | 812.5→307.3 | -80 | -50 |  |  |
| 371 | PS(18:1/20:3) | 810.5→305.2 | -80 | -50 |  |  |
| 372 | PS(18:1/22:4) | 836.5→331.3 | -80 | -50 |  |  |
| 373 | PS(18:1/22:5) | 834.5→329.2 | -80 | -50 |  |  |
| 374 | PS(18:1/22:6) | 832.5→327.2 | -80 | -50 |  |  |
| 375 | PS(18:2/18:2) | 782.5→279.2 | -80 | -50 |  |  |
| 376 | PS(18:2/18:3) | 780.5→277.2 | -80 | -50 |  |  |
| 377 | PS(18:2/20:3) | 808.5→305.2 | -80 | -50 |  |  |
| 378 | PS(18:2/20:4) | 806.5→303.2 | -80 | -50 |  |  |
| 379 | PS(18:2/20:5) | 804.5→301.2 | -80 | -50 |  |  |
| 380 | PS(18:2/22:4) | 834.5→331.3 | -80 | -50 |  |  |
| 381 | PS(18:2/22:6) | 830.5→327.2 | -80 | -50 |  |  |
| 382 | PS(18:3/18:3) | 778.5→277.2 | -80 | -50 |  |  |
| 383 | PS(18:3/20:1) | 810.5→309.3 | -80 | -50 |  |  |
| 384 | PS(18:3/20:2) | 808.5→307.3 | -80 | -50 |  |  |
| 385 | PS(18:3/20:3) | 806.5→305.2 | -80 | -50 |  |  |
| 386 | PS(18:3/20:4) | 804.5→303.2 | -80 | -50 |  | + |
| 387 | PS(18:3/20:5) | 802.5→301.2 | -80 | -50 |  |  |
| 388 | PS(18:3/22:4) | 832.5→331.3 | -80 | -50 |  |  |
| 389 | PS(18:3/22:5) | 830.5→329.2 | -80 | -50 |  |  |
| 390 | PS(18:3/22:6) | 828.5→327.2 | -80 | -50 |  |  |
| 391 | PS(20:0/20:1) | 844.6→309.3 | -80 | -50 |  |  |
| 392 | PS(20:0/20:2) | 842.6→307.3 | -80 | -50 |  |  |
| 393 | PS(20:0/20:3) | 840.6→305.2 | -80 | -50 |  |  |
| 394 | PS(20:0/20:4) | 838.6→303.2 | -80 | -50 |  | + |
| 395 | PS(20:0/20:5) | 836.5→301.2 | -80 | -50 |  |  |
| 396 | PS(20:0/22:4) | 866.6→331.3 | -80 | -50 |  |  |
| 397 | PS(20:0/22:5) | 864.6→329.2 | -80 | -50 |  |  |
| 398 | PS(20:0/22:6) | 862.6→327.2 | -80 | -50 |  |  |
| 399 | PS(20:1/22:4) | 864.6→331.3 | -80 | -50 |  |  |
| 400 | PS(20:1/22:6) | 860.5→327.2 | -80 | -50 |  |  |
| 401 | PS(20:2/20:4) | 834.5→303.2 | -80 | -50 |  |  |
| 402 | PS(20:2/20:5) | 832.5→301.2 | -80 | -50 |  |  |
| 403 | PS(20:2/22:4) | 890.6→331.3 | -80 | -50 |  |  |
| 404 | PS(20:2/22:5) | 888.6→329.2 | -80 | -50 |  |  |
| 405 | PS(20:2/22:6) | 886.6→327.2 | -80 | -50 |  |  |
| 406 | PS(20:3/20:3) | 834.5→305.2 | -80 | -50 |  |  |
| 407 | PS(20:3/20:4) | 832.5→303.2 | -80 | -50 |  |  |
| 408 | PS(20:3/20:5) | 830.5→301.2 | -80 | -50 |  |  |
| 409 | PS(20:3/22:4) | 860.5→331.3 | -80 | -50 |  |  |
| 410 | PS(20:3/22:5) | 858.5→329.2 | -80 | -50 |  |  |
| 411 | PS(20:3/22:6) | 856.5→327.2 | -80 | -50 |  |  |
| 412 | PS(20:4/20:4) | 830.5→303.2 | -80 | -50 |  |  |
| 413 | PS(20:4/20:5) | 828.5→301.2 | -80 | -50 |  |  |
| 414 | PS(20:4/22:4) | 858.5→331.3 | -80 | -50 |  |  |
| 415 | PS(20:4/22:5) | 856.5→329.2 | -80 | -50 |  |  |
| 416 | PS(20:4/22:6) | 854.5→327.2 | -80 | -50 |  |  |
| 417 | PS(20:5/20:5) | 826.5→301.2 | -80 | -50 |  |  |
| 418 | PS(20:5/22:4) | 856.5→331.3 | -80 | -50 |  |  |
| 419 | PS(20:5/22:5) | 854.5→329.2 | -80 | -50 |  |  |
| 420 | PS(20:5/22:6) | 852.5→327.2 | -80 | -50 |  |  |
| 421 | PS(22:4/22:4) | 886.6→331.3 | -80 | -50 |  |  |
| 422 | PS(22:4/22:5) | 884.5→329.2 | -80 | -50 |  |  |
| 423 | PS(22:4/22:6) | 882.5→327.2 | -80 | -50 |  |  |
| 424 | PS(22:6/22:6) | 878.5→327.2 | -80 | -50 |  |  |
| 425 | PA(14:0/18:0) | 647.5→283.3 | -80 | -50 |  |  |
| 426 | PA(14:0/18:1) | 645.5→281.2 | -80 | -50 |  | + |
| 427 | PA(16:0/16:0) | 647.5→255.2 | -80 | -50 |  | + |
| 428 | PA(16:0/16:1) | 645.5→253.2 | -80 | -50 |  |  |
| 429 | PA(16:0/17:0) | 661.5→269.2 | -80 | -50 |  |  |
| 430 | PA(16:0/18:0) | 675.5→283.3 | -80 | -50 |  |  |
| 431 | PA(16:0/18:1) | 673.5→281.2 | -80 | -50 | + | + |
| 432 | PA(16:0/20:1) | 701.5→309.3 | -80 | -50 |  |  |
| 433 | PA(16:0/20:2) | 699.5→307.3 | -80 | -50 |  |  |
| 434 | PA(16:0/20:3) | 697.5→305.2 | -80 | -50 |  | + |
| 435 | PA(16:0/20:4) | 695.5→303.2 | -80 | -50 |  | + |
| 436 | PA(16:0/20:5) | 693.5→301.2 | -80 | -50 |  |  |
| 437 | PA(16:1/18:0) | 673.5→283.3 | -80 | -50 |  |  |
| 438 | PA(18:0/18:1) | 701.5→281.2 | -80 | -50 |  | + |
| 439 | PA(18:0/18:2) | 699.5→279.2 | -80 | -50 |  | + |
| 440 | PA(18:0/18:3) | 697.5→277.2 | -80 | -50 |  |  |
| 441 | PA(18:0/20:1) | 729.5→309.3 | -80 | -50 |  |  |
| 442 | PA(18:0/20:2) | 727.5→307.3 | -80 | -50 |  |  |
| 443 | PA(18:0/20:3) | 725.5→305.2 | -80 | -50 |  | + |
| 444 | PA(18:0/20:4) | 723.5→303.2 | -80 | -50 |  | + |
| 445 | PA(18:0/20:5) | 721.5→301.2 | -80 | -50 |  |  |
| 446 | PA(18:0/22:4) | 751.5→331.3 | -80 | -50 |  | + |
| 447 | PA(18:0/22:5) | 749.5→329.2 | -80 | -50 |  | + |
| 448 | PA(18:0/22:6) | 747.5→327.2 | -80 | -50 |  |  |
| 449 | PA(18:1/18:1) | 699.5→281.2 | -80 | -50 | + | + |
| 450 | PA(18:1/18:2) | 697.5→279.2 | -80 | -50 |  | + |
| 451 | PA(18:1/18:3) | 695.5→277.2 | -80 | -50 |  |  |
| 452 | PA(18:1/20:1) | 727.5→309.3 | -80 | -50 |  |  |
| 453 | PA(18:1/20:2) | 725.5→307.3 | -80 | -50 |  |  |
| 454 | PA(18:1/20:3) | 723.5→305.2 | -80 | -50 |  | + |
| 455 | PA(18:1/20:4) | 721.5→303.2 | -80 | -50 |  | + |
| 456 | PA(18:1/20:5) | 719.5→301.2 | -80 | -50 |  |  |
| 457 | PA(18:1/22:4) | 749.5→331.3 | -80 | -50 |  |  |
| 458 | PA(18:1/22:5) | 747.5→329.2 | -80 | -50 |  |  |
| 459 | PA(18:1/22:6) | 745.5→327.2 | -80 | -50 |  |  |
| 460 | PA(18:2/18:2) | 695.5→279.2 | -80 | -50 |  | + |
| 461 | PA(18:2/19:0) | 713.5→297.3 | -80 | -50 |  |  |
| 462 | PA(18:2/20:0) | 727.5→311.3 | -80 | -50 |  |  |
| 463 | PA(18:2/20:1) | 725.5→309.3 | -80 | -50 |  |  |
| 464 | PA(18:2/20:2) | 723.5→307.3 | -80 | -50 |  |  |
| 465 | PA(18:2/20:3) | 721.5→305.2 | -80 | -50 |  |  |
| 466 | PA(18:2/20:4) | 719.5→303.2 | -80 | -50 |  |  |
| 467 | PA(18:3/18:3) | 691.4→277.2 | -80 | -50 |  |  |
| 468 | PA(18:3/20:5) | 715.4→301.2 | -80 | -50 |  |  |
| 469 | PA(20:0/20:1) | 757.6→309.3 | -80 | -50 |  |  |
| 470 | PA(20:0/20:2) | 755.6→307.3 | -80 | -50 |  |  |
| 471 | PA(20:0/20:3) | 753.5→305.2 | -80 | -50 |  |  |
| 472 | PA(20:0/20:4) | 751.5→303.2 | -80 | -50 |  | + |
| 473 | PA(20:0/20:5) | 749.5→301.2 | -80 | -50 |  |  |
| 474 | PA(20:0/22:4) | 779.5→331.3 | -80 | -50 |  | + |
| 475 | PA(20:0/22:5) | 777.5→329.2 | -80 | -50 |  |  |
| 476 | PA(20:0/22:6) | 775.5→327.2 | -80 | -50 |  |  |
| 477 | PA(20:1/20:1) | 755.6→309.3 | -80 | -50 |  |  |
| 478 | PA(20:1/20:2) | 753.5→307.3 | -80 | -50 |  |  |
| 479 | PA(20:1/20:3) | 751.5→305.2 | -80 | -50 |  | + |
| 480 | PA(20:1/20:4) | 749.5→303.2 | -80 | -50 |  |  |
| 481 | PA(20:1/20:5) | 747.5→301.2 | -80 | -50 |  |  |
| 482 | PA(20:1/22:4) | 777.5→331.3 | -80 | -50 |  | + |
| 483 | PA(20:1/22:5) | 775.5→329.2 | -80 | -50 |  |  |
| 484 | PA(20:1/22:6) | 773.5→327.2 | -80 | -50 |  |  |
| 485 | PA(20:2/20:4) | 747.5→303.2 | -80 | -50 |  | + |
| 486 | PA(20:2/20:5) | 745.5→301.2 | -80 | -50 |  |  |
| 487 | PA(20:2/22:4) | 803.6→331.3 | -80 | -50 |  |  |
| 488 | PA(20:2/22:5) | 801.5→329.2 | -80 | -50 |  |  |
| 489 | PA(20:2/22:6) | 799.5→327.2 | -80 | -50 |  |  |
| 490 | PA(20:3/20:4) | 745.5→303.2 | -80 | -50 |  |  |
| 491 | PA(20:3/22:6) | 769.5→327.2 | -80 | -50 |  |  |
| 492 | PA(20:5/20:5) | 739.4→301.2 | -80 | -50 |  |  |
| 493 | PA(20:5/22:4) | 769.5→331.3 | -80 | -50 |  |  |
| 494 | PA(20:5/22:6) | 765.5→327.2 | -80 | -50 |  | + |
| 495 | PA(22:5/22:5) | 795.5→329.2 | -80 | -50 |  |  |
| 496 | PA(22:5/22:6) | 793.5→327.2 | -80 | -50 |  |  |
| 497 | PA(22:6/22:6) | 791.5→327.2 | -80 | -50 |  | + |
| 498 | PI(14:0/16:0) | 781.5→255.2 | -80 | -60 |  |  |
| 499 | PI(14:0/18:1) | 807.5→281.2 | -80 | -60 |  |  |
| 500 | PI(14:0/18:2) | 805.5→279.2 | -80 | -60 |  |  |
| 501 | PI(14:0/20:3) | 831.5→305.2 | -80 | -60 |  |  |
| 502 | PI(14:0/20:4) | 829.5→303.2 | -80 | -60 |  |  |
| 503 | PI(14:0/22:4) | 857.5→331.3 | -80 | -60 |  |  |
| 504 | PI(14:0/22:5) | 855.5→329.2 | -80 | -60 |  |  |
| 505 | PI(14:0/22:6) | 853.5→327.2 | -80 | -60 |  |  |
| 506 | PI(16:0/16:1) | 807.5→253.2 | -80 | -60 |  |  |
| 507 | PI(16:0/18:1) | 835.5→281.2 | -80 | -60 |  | + |
| 508 | PI(16:0/18:2) | 833.5→279.2 | -80 | -60 |  | + |
| 509 | PI(16:0/18:3) | 814.6→277.2 | -80 | -60 |  | + |
| 510 | PI(16:0/20:1) | 863.6→309.3 | -80 | -60 |  |  |
| 511 | PI(16:0/20:2) | 861.6→307.3 | -80 | -60 |  |  |
| 512 | PI(16:0/20:3) | 859.5→305.2 | -80 | -60 |  | + |
| 513 | PI(16:0/20:4) | 857.5→303.2 | -80 | -60 |  | + |
| 514 | PI(16:0/20:5) | 855.5→301.2 | -80 | -60 |  |  |
| 515 | PI(16:0/22:4) | 885.6→331.3 | -80 | -60 |  |  |
| 516 | PI(16:0/22:5) | 883.5→329.2 | -80 | -60 |  |  |
| 517 | PI(16:0/22:6) | 881.5→327.2 | -80 | -60 |  |  |
| 518 | PI(16:1/18:0) | 835.5→283.3 | -80 | -60 |  | + |
| 519 | PI(16:1/18:1) | 833.5→281.2 | -80 | -60 |  |  |
| 520 | PI(16:1/18:2) | 831.5→279.2 | -80 | -60 |  |  |
| 521 | PI(16:1/18:3) | 829.5→277.2 | -80 | -60 |  |  |
| 522 | PI(16:1/20:0) | 863.6→253.2 | -80 | -60 |  |  |
| 523 | PI(16:1/20:1) | 861.5→309.3 | -80 | -60 |  |  |
| 524 | PI(16:1/20:2) | 859.5→307.3 | -80 | -60 |  |  |
| 525 | PI(16:1/20:3) | 857.5→305.2 | -80 | -60 |  |  |
| 526 | PI(16:1/20:4) | 855.5→303.2 | -80 | -60 |  |  |
| 527 | PI(16:1/20:5) | 853.5→301.2 | -80 | -60 |  |  |
| 528 | PI(16:1/22:4) | 883.5→331.3 | -80 | -60 |  |  |
| 529 | PI(16:1/22:5) | 881.5→329.2 | -80 | -60 |  |  |
| 530 | PI(16:1/22:6) | 879.5→327.2 | -80 | -60 |  |  |
| 531 | PI(18:0/18:0) | 865.6→283.3 | -80 | -60 |  |  |
| 532 | PI(18:0/18:1) | 863.6→281.2 | -80 | -60 |  |  |
| 533 | PI(18:0/18:2) | 861.6→279.2 | -80 | -60 |  |  |
| 534 | PI(18:0/18:3) | 859.5→277.2 | -80 | -60 |  |  |
| 535 | PI(18:0/20:0) | 893.6→311.3 | -80 | -60 |  |  |
| 536 | PI(18:0/20:1) | 891.6→309.3 | -80 | -60 |  |  |
| 537 | PI(18:0/20:2) | 889.6→307.3 | -80 | -60 |  |  |
| 538 | PI(18:0/20:3) | 887.6→305.2 | -80 | -60 |  | + |
| 539 | PI(18:0/20:4) | 885.6→303.2 | -80 | -60 | + | + |
| 540 | PI(18:0/20:5) | 883.5→301.2 | -80 | -60 |  |  |
| 541 | PI(18:0/22:4) | 913.6→331.3 | -80 | -60 |  |  |
| 542 | PI(18:0/22:5) | 911.6→329.2 | -80 | -60 |  |  |
| 543 | PI(18:0/22:6) | 909.6→327.2 | -80 | -60 |  |  |
| 544 | PI(18:1/18:1) | 861.6→281.2 | -80 | -60 |  | + |
| 545 | PI(18:1/18:2) | 859.5→279.2 | -80 | -60 |  |  |
| 546 | PI(18:1/18:3) | 857.5→277.2 | -80 | -60 |  |  |
| 547 | PI(18:1/20:1) | 889.6→309.3 | -80 | -60 |  |  |
| 548 | PI(18:1/20:2) | 887.6→307.3 | -80 | -60 |  |  |
| 549 | PI(18:1/20:3) | 885.6→305.2 | -80 | -60 |  |  |
| 550 | PI(18:1/20:4) | 883.5→303.2 | -80 | -60 |  |  |
| 551 | PI(18:1/20:5) | 881.5→301.2 | -80 | -60 |  |  |
| 552 | PI(18:1/22:4) | 911.6→331.3 | -80 | -60 |  |  |
| 553 | PI(18:1/22:5) | 909.6→329.2 | -80 | -60 |  |  |
| 554 | PI(18:1/22:6) | 907.5→327.2 | -80 | -60 |  |  |
| 555 | PI(18:2/18:2) | 857.5→279.2 | -80 | -60 |  |  |
| 556 | PI(18:2/18:3) | 855.5→277.2 | -80 | -60 |  |  |
| 557 | PI(18:2/20:0) | 889.6→311.3 | -80 | -60 |  |  |
| 558 | PI(18:2/20:1) | 887.6→309.3 | -80 | -60 |  |  |
| 559 | PI(18:2/20:2) | 885.6→307.3 | -80 | -60 |  |  |
| 560 | PI(18:2/20:3) | 883.5→305.2 | -80 | -60 |  |  |
| 561 | PI(18:2/20:4) | 881.5→303.2 | -80 | -60 |  |  |
| 562 | PI(18:2/22:4) | 909.6→331.3 | -80 | -60 |  |  |
| 563 | PI(18:2/22:6) | 905.5→327.2 | -80 | -60 |  |  |
| 564 | LPC(14:0) | 526.3→227.2 | -80 | -50 | + |  |
| 565 | LPC(16:0) | 554.3→255.2 | -80 | -50 | + | + |
| 566 | LPC(16:1) | 552.3→253.2 | -80 | -50 | + | + |
| 567 | LPC(18:0) | 582.4→283.3 | -80 | -50 | + | + |
| 568 | LPC(18:1) | 580.4→281.2 | -80 | -50 | + | + |
| 569 | LPC(18:2) | 578.3→279.2 | -80 | -50 | + | + |
| 570 | LPC(18:3) | 576.3→277.2 | -80 | -50 | + | + |
| 571 | LPC(20:0) | 610.4→311.3 | -80 | -50 | + | + |
| 572 | LPC(20:1) | 608.4→309.3 | -80 | -50 | + | + |
| 573 | LPC(20:2) | 606.4→307.3 | -80 | -50 | + |  |
| 574 | LPC(20:3) | 604.4→305.2 | -80 | -50 | + | + |
| 575 | LPC(20:4) | 602.3→303.2 | -80 | -50 | + | + |
| 576 | LPC(20:5) | 600.3→301.2 | -80 | -50 | + |  |
| 577 | LPC(22:4) | 630.4→331.3 | -80 | -50 | + | + |
| 578 | LPC(22:5) | 628.4→329.2 | -80 | -50 | + |  |
| 579 | LPC(22:6) | 626.3→327.2 | -80 | -50 | + | + |
| 580 | LPE(16:0) | 452.3→255.2 | -80 | -50 | + | + |
| 581 | LPE(16:1) | 450.3→253.2 | -80 | -50 | + | + |
| 582 | LPE(17:0) | 466.3→269.2 | -80 | -50 | + | + |
| 583 | LPE(18:0) | 480.3→283.3 | -80 | -50 | + | + |
| 584 | LPE(18:1) | 478.3→281.2 | -80 | -50 | + | + |
| 585 | LPE(18:2) | 476.3→279.2 | -80 | -50 | + | + |
| 586 | LPE(18:3) | 474.3→277.2 | -80 | -50 |  | + |
| 587 | LPE(19:0) | 494.3→297.3 | -80 | -50 | + | + |
| 588 | LPE(20:0) | 508.3→311.3 | -80 | -50 | + | + |
| 589 | LPE(20:1) | 506.3→309.3 | -80 | -50 | + | + |
| 590 | LPE(20:2) | 504.3→307.3 | -80 | -50 | + | + |
| 591 | LPE(20:3) | 502.3→305.2 | -80 | -50 | + | + |
| 592 | LPE(20:4) | 500.3→303.2 | -80 | -50 | + | + |
| 593 | LPE(22:4) | 528.3→331.3 | -80 | -50 |  | + |
| 594 | LPE(22:5) | 526.3→329.2 | -80 | -50 |  | + |
| 595 | LPE(22:6) | 524.3→327.2 | -80 | -50 |  | + |
| 596 | LPI(16:0) | 571.3→255.2 | -80 | -50 |  |  |
| 597 | LPI(18:2) | 595.3→279.2 | -80 | -50 |  |  |
| 598 | LPI(18:3) | 593.3→277.2 | -80 | -50 |  |  |
| 599 | LPI(20:2) | 623.3→307.3 | -80 | -50 |  |  |
| 600 | LPI(20:3) | 621.3→305.2 | -80 | -50 |  | + |
| 601 | LPI(20:4) | 619.3→303.2 | -80 | -50 |  | + |
| 602 | LPG(14:0) | 455.2→227.2 | -80 | -50 |  |  |
| 603 | LPG(18:0) | 511.3→283.3 | -80 | -50 |  | + |
| 604 | LPG(18:1) | 509.3→281.2 | -80 | -50 |  | + |
| 605 | LPG(18:2) | 507.3→279.2 | -80 | -50 |  | + |
| 606 | LPS(16:0) | 496.3→255.2 | -80 | -50 |  |  |
| 607 | LPS(18:0) | 524.3→283.3 | -80 | -50 |  |  |
| 608 | LPS(18:1) | 522.3→281.2 | -80 | -50 |  |  |
| 609 | LPS(18:2) | 520.3→279.2 | -80 | -50 |  |  |
| 610 | FFA(14:0) | 227.1→227.1 | -80 | -10 | + | + |
| 611 | FFA(14:1) | 225.1→225.1 | -80 | -10 | + | + |
| 612 | FFA(16:0) | 255.1→255.1 | -80 | -10 | + | + |
| 613 | FFA(16:1) | 253.1→253.1 | -80 | -10 | + | + |
| 614 | FFA(16:2) | 251.1→251.1 | -80 | -10 | + | + |
| 615 | FFA(18:0) | 283.2→283.2 | -80 | -10 | + |  |
| 616 | FFA(18:1) | 281.2→281.2 | -80 | -10 | + | + |
| 617 | FFA(18:2) | 279.2→279.2 | -80 | -10 | + | + |
| 618 | FFA(18:3) | 277.2→277.2 | -80 | -10 |  | + |
| 619 | FFA(20:0) | 311.2→311.2 | -80 | -10 | + |  |
| 620 | FFA(20:1) | 309.2→309.2 | -80 | -10 | + | + |
| 621 | FFA(20:2) | 307.2→307.2 | -80 | -10 | + | + |
| 622 | FFA(20:3) | 305.2→305.2 | -80 | -10 | + | + |
| 623 | FFA(20:4) | 303.2→303.2 | -80 | -10 | + | + |
| 624 | FFA(20:5) | 301.2→301.2 | -80 | -10 | + | + |
| 625 | FFA(22:4) | 331.3→331.3 | -80 | -10 | + | + |
| 626 | FFA(22:5) | 329.2→329.2 | -80 | -10 | + | + |
| 627 | FFA(22:6) | 327.2→327.2 | -80 | -10 | + | + |
| 628 | FFA(24:0) | 367.3→367.3 | -80 | -10 | + |  |
| 629 | FFA(24:1) | 365.3→365.3 | -80 | -10 | + | + |
| 630 | TAG(40:0/FA14:0) | 712.6→467.4 | 60 | 38 | + | + |
| 631 | TAG(40:0/FA16:0) | 712.6→439.4 | 60 | 38 | + | + |
| 632 | TAG(42:0/FA14:0) | 740.7→495.4 | 60 | 38 | + | + |
| 633 | TAG(42:0/FA16:0) | 740.7→467.4 | 60 | 38 | + | + |
| 634 | TAG(42:1/FA14:0) | 738.7→493.4 | 60 | 38 | + | + |
| 635 | TAG(42:1/FA16:0) | 738.7→465.4 | 60 | 38 | + | + |
| 636 | TAG(42:1/FA16:1) | 738.7→467.4 | 60 | 38 | + | + |
| 637 | TAG(42:1/FA18:1) | 738.7→439.4 | 60 | 38 | + | + |
| 638 | TAG(42:2/FA18:2) | 736.7→439.4 | 60 | 38 | + | + |
| 639 | TAG(44:0/FA14:0) | 768.7→523.5 | 60 | 38 | + | + |
| 640 | TAG(44:0/FA16:0) | 768.7→495.4 | 60 | 38 | + | + |
| 641 | TAG(44:0/FA18:0) | 768.7→467.4 | 60 | 38 | + | + |
| 642 | TAG(44:1/FA14:0) | 766.7→521.5 | 60 | 38 | + | + |
| 643 | TAG(44:1/FA16:0) | 766.7→493.4 | 60 | 38 | + | + |
| 644 | TAG(44:1/FA16:1) | 766.7→495.4 | 60 | 38 | + | + |
| 645 | TAG(44:1/FA18:1) | 766.7→467.4 | 60 | 38 | + | + |
| 646 | TAG(44:2/FA14:0) | 764.7→519.4 | 60 | 38 |  | + |
| 647 | TAG(44:2/FA16:0) | 764.7→491.4 | 60 | 38 |  | + |
| 648 | TAG(44:2/FA16:1) | 764.7→493.4 | 60 | 38 | + | + |
| 649 | TAG(44:2/FA18:1) | 764.7→465.4 | 60 | 38 | + | + |
| 650 | TAG(44:2/FA18:2) | 764.7→467.4 | 60 | 38 | + | + |
| 651 | TAG(44:3/FA18:2) | 762.7→465.4 | 60 | 38 |  | + |
| 652 | TAG(45:0/FA14:0) | 782.7→537.5 | 60 | 38 |  | + |
| 653 | TAG(45:0/FA16:0) | 782.7→509.5 | 60 | 38 |  | + |
| 654 | TAG(45:1/FA16:0) | 780.7→507.4 | 60 | 38 |  | + |
| 655 | TAG(45:1/FA18:1) | 780.7→481.4 | 60 | 38 |  | + |
| 656 | TAG(46:0/FA14:0) | 796.7→551.5 | 60 | 38 | + | + |
| 657 | TAG(46:0/FA16:0) | 796.7→523.5 | 60 | 38 | + | + |
| 658 | TAG(46:1/FA14:0) | 794.7→549.5 | 60 | 38 | + | + |
| 659 | TAG(46:1/FA16:0) | 794.7→521.4 | 60 | 38 | + | + |
| 660 | TAG(46:1/FA16:1) | 794.7→523.5 | 60 | 38 | + | + |
| 661 | TAG(46:1/FA18:0) | 794.7→493.4 | 60 | 38 |  | + |
| 662 | TAG(46:1/FA18:1) | 794.7→495.4 | 60 | 38 | + | + |
| 663 | TAG(46:2/FA14:0) | 792.7→547.5 | 60 | 38 | + | + |
| 664 | TAG(46:2/FA16:0) | 792.7→519.4 | 60 | 38 | + | + |
| 665 | TAG(46:2/FA16:1) | 792.7→521.4 | 60 | 38 | + | + |
| 666 | TAG(46:2/FA18:1) | 792.7→493.4 | 60 | 38 | + | + |
| 667 | TAG(46:2/FA18:2) | 792.7→495.4 | 60 | 38 | + | + |
| 668 | TAG(46:3/FA14:0) | 790.7→545.5 | 60 | 38 |  | + |
| 669 | TAG(46:3/FA16:0) | 790.7→517.4 | 60 | 38 |  | + |
| 670 | TAG(46:3/FA16:1) | 790.7→519.4 | 60 | 38 | + | + |
| 671 | TAG(46:3/FA18:1) | 790.7→491.4 | 60 | 38 |  | + |
| 672 | TAG(46:3/FA18:2) | 790.7→493.4 | 60 | 38 | + | + |
| 673 | TAG(46:3/FA18:3) | 790.7→495.4 | 60 | 38 |  | + |
| 674 | TAG(46:4/FA18:2) | 788.7→491.4 | 60 | 38 |  | + |
| 675 | TAG(47:0/FA14:0) | 810.7→565.5 | 60 | 38 |  | + |
| 676 | TAG(47:0/FA16:0) | 810.7→537.4 | 60 | 38 |  | + |
| 677 | TAG(47:0/FA17:0) | 810.7→523.5 | 60 | 38 |  | + |
| 678 | TAG(47:1/FA14:0) | 808.7→563.5 | 60 | 38 |  | + |
| 679 | TAG(47:1/FA16:0) | 808.7→535.4 | 60 | 38 |  | + |
| 680 | TAG(47:1/FA16:1) | 808.7→537.4 | 60 | 38 |  | + |
| 681 | TAG(47:1/FA18:1) | 808.7→509.4 | 60 | 38 |  | + |
| 682 | TAG(47:2/FA14:0) | 806.7→561.5 | 60 | 38 |  | + |
| 683 | TAG(47:2/FA16:1) | 806.7→535.4 | 60 | 38 |  | + |
| 684 | TAG(47:2/FA18:1) | 806.7→507.4 | 60 | 38 |  | + |
| 685 | TAG(47:2/FA18:2) | 806.7→509.4 | 60 | 38 |  | + |
| 686 | TAG(48:0/FA14:0) | 824.7→579.5 | 60 | 38 |  | + |
| 687 | TAG(48:0/FA16:0) | 824.7→551.4 | 60 | 38 | + | + |
| 688 | TAG(48:0/FA18:0) | 824.7→523.5 | 60 | 38 |  | + |
| 689 | TAG(48:1/FA14:0) | 822.7→577.5 | 60 | 38 | + | + |
| 690 | TAG(48:1/FA16:0) | 822.7→549.4 | 60 | 38 | + | + |
| 691 | TAG(48:1/FA16:1) | 822.7→551.4 | 60 | 38 | + | + |
| 692 | TAG(48:1/FA18:0) | 822.7→521.4 | 60 | 38 | + | + |
| 693 | TAG(48:1/FA18:1) | 822.7→523.5 | 60 | 38 |  | + |
| 694 | TAG(48:2/FA14:0) | 820.7→575.5 | 60 | 38 | + | + |
| 695 | TAG(48:2/FA16:0) | 820.7→547.4 | 60 | 38 | + | + |
| 696 | TAG(48:2/FA16:1) | 820.7→549.4 | 60 | 38 | + | + |
| 697 | TAG(48:2/FA18:0) | 820.7→519.4 | 60 | 38 | + | + |
| 698 | TAG(48:2/FA18:1) | 820.7→521.4 | 60 | 38 | + | + |
| 699 | TAG(48:2/FA18:2) | 820.7→523.5 | 60 | 38 | + | + |
| 700 | TAG(48:3/FA14:0) | 818.7→573.5 | 60 | 38 | + | + |
| 701 | TAG(48:3/FA16:0) | 818.7→545.4 | 60 | 38 | + | + |
| 702 | TAG(48:3/FA16:1) | 818.7→547.4 | 60 | 38 | + | + |
| 703 | TAG(48:3/FA18:1) | 818.7→519.4 | 60 | 38 | + | + |
| 704 | TAG(48:3/FA18:2) | 818.7→521.4 | 60 | 38 | + | + |
| 705 | TAG(48:3/FA18:3) | 818.7→523.5 | 60 | 38 |  | + |
| 706 | TAG(48:4/FA14:0) | 816.7→571.5 | 60 | 38 |  | + |
| 707 | TAG(48:4/FA16:0) | 816.7→543.4 | 60 | 38 |  | + |
| 708 | TAG(48:4/FA16:1) | 816.7→545.4 | 60 | 38 |  | + |
| 709 | TAG(48:4/FA18:1) | 816.7→517.4 | 60 | 38 |  | + |
| 710 | TAG(48:4/FA18:2) | 816.7→519.4 | 60 | 38 | + | + |
| 711 | TAG(48:4/FA18:3) | 816.7→521.4 | 60 | 38 |  | + |
| 712 | TAG(48:4/FA20:4) | 816.7→495.4 | 60 | 38 | + | + |
| 713 | TAG(48:5/FA18:2) | 814.7→517.4 | 60 | 38 |  | + |
| 714 | TAG(48:5/FA18:3) | 814.7→519.4 | 60 | 38 |  | + |
| 715 | TAG(49:0/FA16:0) | 838.8→565.5 | 60 | 38 |  | + |
| 716 | TAG(49:0/FA18:0) | 838.8→537.5 | 60 | 38 |  |  |
| 717 | TAG(49:1/FA14:0) | 836.8→591.6 | 60 | 38 |  | + |
| 718 | TAG(49:1/FA16:0) | 836.8→563.5 | 60 | 38 |  | + |
| 719 | TAG(49:1/FA16:1) | 836.8→565.5 | 60 | 38 |  | + |
| 720 | TAG(49:1/FA18:1) | 836.8→537.5 | 60 | 38 |  | + |
| 721 | TAG(49:2/FA14:0) | 834.8→589.6 | 60 | 38 |  | + |
| 722 | TAG(49:2/FA16:0) | 834.8→561.5 | 60 | 38 |  | + |
| 723 | TAG(49:2/FA16:1) | 834.8→563.5 | 60 | 38 |  | + |
| 724 | TAG(49:2/FA18:1) | 834.8→535.5 | 60 | 38 |  | + |
| 725 | TAG(49:2/FA18:2) | 834.8→537.5 | 60 | 38 |  | + |
| 726 | TAG(49:3/FA16:0) | 832.8→559.5 | 60 | 38 |  | + |
| 727 | TAG(49:3/FA16:1) | 832.8→561.5 | 60 | 38 |  | + |
| 728 | TAG(49:3/FA18:2) | 832.8→535.5 | 60 | 38 |  | + |
| 729 | TAG(49:3/FA18:3) | 832.8→537.5 | 60 | 38 |  |  |
| 730 | TAG(50:0/FA14:0) | 852.8→607.6 | 60 | 38 |  | + |
| 731 | TAG(50:0/FA16:0) | 852.8→579.5 | 60 | 38 |  | + |
| 732 | TAG(50:0/FA18:0) | 852.8→551.5 | 60 | 38 |  | + |
| 733 | TAG(50:1/FA14:0) | 850.8→605.6 | 60 | 38 | + | + |
| 734 | TAG(50:1/FA16:0) | 850.8→577.5 | 60 | 38 | + | + |
| 735 | TAG(50:1/FA16:1) | 850.8→579.5 | 60 | 38 | + | + |
| 736 | TAG(50:1/FA18:0) | 850.8→549.5 | 60 | 38 | + | + |
| 737 | TAG(50:1/FA18:1) | 850.8→551.5 | 60 | 38 | + | + |
| 738 | TAG(50:1/FA20:1) | 850.8→523.5 | 60 | 38 |  |  |
| 739 | TAG(50:2/FA14:0) | 848.8→603.6 | 60 | 38 | + | + |
| 740 | TAG(50:2/FA16:0) | 848.8→575.5 | 60 | 38 | + | + |
| 741 | TAG(50:2/FA16:1) | 848.8→577.5 | 60 | 38 | + | + |
| 742 | TAG(50:2/FA18:0) | 848.8→547.5 | 60 | 38 |  | + |
| 743 | TAG(50:2/FA18:1) | 848.8→549.5 | 60 | 38 | + | + |
| 744 | TAG(50:2/FA18:2) | 848.8→551.5 | 60 | 38 | + | + |
| 745 | TAG(50:2/FA20:2) | 848.8→523.5 | 60 | 38 |  |  |
| 746 | TAG(50:3/FA14:0) | 846.8→601.6 | 60 | 38 | + | + |
| 747 | TAG(50:3/FA16:0) | 846.8→573.5 | 60 | 38 | + | + |
| 748 | TAG(50:3/FA16:1) | 846.8→575.5 | 60 | 38 | + | + |
| 749 | TAG(50:3/FA18:0) | 846.8→545.5 | 60 | 38 |  | + |
| 750 | TAG(50:3/FA18:1) | 846.8→547.5 | 60 | 38 | + | + |
| 751 | TAG(50:3/FA18:2) | 846.8→549.5 | 60 | 38 | + | + |
| 752 | TAG(50:3/FA18:3) | 846.8→551.5 | 60 | 38 |  | + |
| 753 | TAG(50:3/FA20:3) | 846.8→523.5 | 60 | 38 |  | + |
| 754 | TAG(50:4/FA14:0) | 844.6→599.4 | 60 | 38 | + | + |
| 755 | TAG(50:4/FA16:0) | 844.6→571.3 | 60 | 38 |  | + |
| 756 | TAG(50:4/FA16:1) | 844.6→573.3 | 60 | 38 | + | + |
| 757 | TAG(50:4/FA18:1) | 844.6→545.3 | 60 | 38 |  | + |
| 758 | TAG(50:4/FA18:2) | 844.6→547.3 | 60 | 38 | + | + |
| 759 | TAG(50:4/FA18:3) | 844.6→549.3 | 60 | 38 | + | + |
| 760 | TAG(50:4/FA20:3) | 844.6→521.3 | 60 | 38 |  | + |
| 761 | TAG(50:4/FA20:4) | 844.6→523.3 | 60 | 38 | + | + |
| 762 | TAG(50:5/FA14:0) | 842.6→597.4 | 60 | 38 |  | + |
| 763 | TAG(50:5/FA16:0) | 842.6→569.3 | 60 | 38 |  | + |
| 764 | TAG(50:5/FA16:1) | 842.6→571.3 | 60 | 38 |  | + |
| 765 | TAG(50:5/FA18:1) | 842.6→543.3 | 60 | 38 |  | + |
| 766 | TAG(50:5/FA18:2) | 842.6→545.3 | 60 | 38 |  | + |
| 767 | TAG(50:5/FA18:3) | 842.6→547.3 | 60 | 38 |  | + |
| 768 | TAG(50:5/FA20:4) | 842.6→521.3 | 60 | 38 |  | + |
| 769 | TAG(50:5/FA20:5) | 842.6→523.3 | 60 | 38 |  |  |
| 770 | TAG(50:6/FA20:4) | 840.7→519.4 | 60 | 38 |  | + |
| 771 | TAG(51:0/FA16:0) | 866.8→593.5 | 60 | 38 |  | + |
| 772 | TAG(51:0/FA18:0) | 866.8→565.5 | 60 | 38 |  | + |
| 773 | TAG(51:1/FA16:0) | 864.8→591.5 | 60 | 38 |  | + |
| 774 | TAG(51:1/FA18:0) | 864.8→563.5 | 60 | 38 |  | + |
| 775 | TAG(51:1/FA18:1) | 864.8→565.5 | 60 | 38 |  | + |
| 776 | TAG(51:2/FA16:0) | 862.8→589.5 | 60 | 38 |  | + |
| 777 | TAG(51:2/FA16:1) | 862.8→591.5 | 60 | 38 |  |  |
| 778 | TAG(51:2/FA18:1) | 862.8→563.5 | 60 | 38 |  |  |
| 779 | TAG(51:2/FA18:2) | 862.8→565.5 | 60 | 38 |  |  |
| 780 | TAG(51:3/FA16:1) | 860.8→589.5 | 60 | 38 |  |  |
| 781 | TAG(51:3/FA17:0) | 860.8→573.5 | 60 | 38 |  |  |
| 782 | TAG(51:3/FA18:2) | 860.8→563.5 | 60 | 38 |  |  |
| 783 | TAG(51:3/FA18:3) | 860.8→565.5 | 60 | 38 |  |  |
| 784 | TAG(51:4/FA16:1) | 858.8→587.5 | 60 | 38 |  |  |
| 785 | TAG(51:4/FA18:2) | 858.8→561.5 | 60 | 38 |  |  |
| 786 | TAG(51:4/FA18:3) | 858.8→563.5 | 60 | 38 |  |  |
| 787 | TAG(51:5/FA18:2) | 856.8→559.5 | 60 | 38 |  |  |
| 788 | TAG(51:5/FA18:3) | 856.8→561.5 | 60 | 38 |  |  |
| 789 | TAG(52:0/FA16:0) | 880.8→607.5 | 60 | 38 |  |  |
| 790 | TAG(52:0/FA18:0) | 880.8→579.5 | 60 | 38 |  |  |
| 791 | TAG(52:1/FA16:0) | 878.8→605.5 | 60 | 38 |  |  |
| 792 | TAG(52:1/FA16:1) | 878.8→607.5 | 60 | 38 |  |  |
| 793 | TAG(52:1/FA18:0) | 878.8→577.5 | 60 | 38 |  |  |
| 794 | TAG(52:1/FA18:1) | 878.8→579.5 | 60 | 38 |  |  |
| 795 | TAG(52:1/FA20:1) | 878.8→551.5 | 60 | 38 |  |  |
| 796 | TAG(52:2/FA14:0) | 876.8→631.6 | 60 | 38 |  |  |
| 797 | TAG(52:2/FA16:0) | 876.8→603.5 | 60 | 38 |  |  |
| 798 | TAG(52:2/FA16:1) | 876.8→605.5 | 60 | 38 |  |  |
| 799 | TAG(52:2/FA18:0) | 876.8→575.5 | 60 | 38 |  |  |
| 800 | TAG(52:2/FA18:1) | 876.8→577.5 | 60 | 38 |  |  |
| 801 | TAG(52:2/FA18:2) | 876.8→579.5 | 60 | 38 |  |  |
| 802 | TAG(52:2/FA20:0) | 876.8→547.5 | 60 | 38 |  |  |
| 803 | TAG(52:2/FA20:1) | 876.8→549.5 | 60 | 38 |  |  |
| 804 | TAG(52:2/FA20:2) | 876.8→551.5 | 60 | 38 |  |  |
| 805 | TAG(52:3/FA14:0) | 874.8→629.6 | 60 | 38 |  |  |
| 806 | TAG(52:3/FA16:0) | 874.8→601.5 | 60 | 38 |  | + |
| 807 | TAG(52:3/FA16:1) | 874.8→603.5 | 60 | 38 |  | + |
| 808 | TAG(52:3/FA18:0) | 874.8→573.5 | 60 | 38 |  |  |
| 809 | TAG(52:3/FA18:1) | 874.8→575.5 | 60 | 38 |  | + |
| 810 | TAG(52:3/FA18:2) | 874.8→577.5 | 60 | 38 |  |  |
| 811 | TAG(52:3/FA18:3) | 874.8→579.5 | 60 | 38 |  |  |
| 812 | TAG(52:3/FA20:0) | 874.8→545.5 | 60 | 38 |  |  |
| 813 | TAG(52:3/FA20:1) | 874.8→547.5 | 60 | 38 |  |  |
| 814 | TAG(52:3/FA20:2) | 874.8→549.5 | 60 | 38 |  |  |
| 815 | TAG(52:3/FA20:3) | 874.8→551.5 | 60 | 38 |  |  |
| 816 | TAG(52:4/FA14:0) | 872.8→627.6 | 60 | 38 |  |  |
| 817 | TAG(52:4/FA16:0) | 872.8→599.5 | 60 | 38 |  |  |
| 818 | TAG(52:4/FA16:1) | 872.8→601.5 | 60 | 38 |  |  |
| 819 | TAG(52:4/FA18:0) | 872.8→571.5 | 60 | 38 |  |  |
| 820 | TAG(52:4/FA18:1) | 872.8→573.5 | 60 | 38 |  |  |
| 821 | TAG(52:4/FA18:2) | 872.8→575.5 | 60 | 38 |  |  |
| 822 | TAG(52:4/FA18:3) | 872.8→577.5 | 60 | 38 |  |  |
| 823 | TAG(52:4/FA20:0) | 872.8→543.5 | 60 | 38 |  |  |
| 824 | TAG(52:4/FA20:2) | 872.8→547.5 | 60 | 38 |  |  |
| 825 | TAG(52:4/FA20:3) | 872.8→549.5 | 60 | 38 |  |  |
| 826 | TAG(52:4/FA20:4) | 872.8→551.5 | 60 | 38 |  |  |
| 827 | TAG(52:4/FA22:4) | 872.8→523.5 | 60 | 38 |  |  |
| 828 | TAG(52:5/FA14:0) | 870.8→625.6 | 60 | 38 |  |  |
| 829 | TAG(52:5/FA16:0) | 870.8→597.5 | 60 | 38 |  |  |
| 830 | TAG(52:5/FA16:1) | 870.8→599.5 | 60 | 38 |  |  |
| 831 | TAG(52:5/FA18:1) | 870.8→571.5 | 60 | 38 |  |  |
| 832 | TAG(52:5/FA18:2) | 870.8→573.5 | 60 | 38 |  |  |
| 833 | TAG(52:5/FA18:3) | 870.8→575.5 | 60 | 38 |  |  |
| 834 | TAG(52:5/FA20:3) | 870.8→547.5 | 60 | 38 |  |  |
| 835 | TAG(52:5/FA20:4) | 870.8→549.5 | 60 | 38 |  |  |
| 836 | TAG(52:5/FA20:5) | 870.8→551.5 | 60 | 38 |  |  |
| 837 | TAG(52:5/FA22:5) | 870.8→523.5 | 60 | 38 |  |  |
| 838 | TAG(52:6/FA14:0) | 868.8→623.6 | 60 | 38 |  |  |
| 839 | TAG(52:6/FA16:0) | 868.8→595.5 | 60 | 38 |  |  |
| 840 | TAG(52:6/FA16:1) | 868.8→597.5 | 60 | 38 |  |  |
| 841 | TAG(52:6/FA18:1) | 868.8→569.5 | 60 | 38 |  |  |
| 842 | TAG(52:6/FA18:2) | 868.8→571.5 | 60 | 38 |  |  |
| 843 | TAG(52:6/FA18:3) | 868.8→573.5 | 60 | 38 |  |  |
| 844 | TAG(52:6/FA20:4) | 868.8→547.5 | 60 | 38 |  |  |
| 845 | TAG(52:6/FA20:5) | 868.8→549.5 | 60 | 38 |  |  |
| 846 | TAG(52:6/FA22:6) | 868.8→523.5 | 60 | 38 |  |  |
| 847 | TAG(52:7/FA16:0) | 866.7→593.4 | 60 | 38 |  |  |
| 848 | TAG(52:7/FA18:1) | 866.7→567.4 | 60 | 38 |  |  |
| 849 | TAG(52:7/FA20:5) | 866.7→547.4 | 60 | 38 |  |  |
| 850 | TAG(52:7/FA22:6) | 866.7→521.4 | 60 | 38 |  |  |
| 851 | TAG(52:8/FA18:2) | 864.8→567.5 | 60 | 38 |  |  |
| 852 | TAG(53:0/FA16:0) | 894.8→621.5 | 60 | 38 |  |  |
| 853 | TAG(53:1/FA16:0) | 892.8→619.5 | 60 | 38 |  |  |
| 854 | TAG(53:1/FA18:0) | 892.8→591.5 | 60 | 38 |  |  |
| 855 | TAG(53:1/FA18:1) | 892.8→593.5 | 60 | 38 |  |  |
| 856 | TAG(53:2/FA16:0) | 890.8→617.5 | 60 | 38 |  |  |
| 857 | TAG(53:2/FA18:1) | 890.8→591.5 | 60 | 38 |  |  |
| 858 | TAG(53:2/FA18:2) | 890.8→593.5 | 60 | 38 |  |  |
| 859 | TAG(53:3/FA16:0) | 888.8→615.5 | 60 | 38 |  |  |
| 860 | TAG(53:3/FA18:2) | 888.8→591.5 | 60 | 38 |  |  |
| 861 | TAG(53:4/FA16:0) | 886.8→613.5 | 60 | 38 |  |  |
| 862 | TAG(53:4/FA18:2) | 886.8→589.5 | 60 | 38 |  |  |
| 863 | TAG(53:4/FA18:3) | 886.8→591.5 | 60 | 38 |  |  |
| 864 | TAG(53:4/FA20:4) | 886.8→565.5 | 60 | 38 |  |  |
| 865 | TAG(53:5/FA20:4) | 884.8→563.5 | 60 | 38 |  |  |
| 866 | TAG(53:6/FA20:4) | 882.8→561.5 | 60 | 38 |  |  |
| 867 | TAG(54:0/FA16:0) | 908.8→635.5 | 60 | 38 |  |  |
| 868 | TAG(54:0/FA18:0) | 908.8→607.5 | 60 | 38 |  |  |
| 869 | TAG(54:1/FA16:0) | 906.8→633.5 | 60 | 38 |  |  |
| 870 | TAG(54:1/FA18:0) | 906.8→605.5 | 60 | 38 |  |  |
| 871 | TAG(54:1/FA18:1) | 906.8→607.5 | 60 | 38 |  |  |
| 872 | TAG(54:1/FA20:0) | 906.8→577.5 | 60 | 38 |  |  |
| 873 | TAG(54:1/FA20:1) | 906.8→579.5 | 60 | 38 |  |  |
| 874 | TAG(54:2/FA16:0) | 904.8→631.5 | 60 | 38 |  |  |
| 875 | TAG(54:2/FA18:0) | 904.8→603.5 | 60 | 38 |  |  |
| 876 | TAG(54:2/FA18:1) | 904.8→605.5 | 60 | 38 |  |  |
| 877 | TAG(54:2/FA18:2) | 904.8→607.5 | 60 | 38 |  |  |
| 878 | TAG(54:2/FA20:0) | 904.8→575.5 | 60 | 38 |  |  |
| 879 | TAG(54:2/FA20:1) | 904.8→577.5 | 60 | 38 |  |  |
| 880 | TAG(54:2/FA20:2) | 904.8→579.5 | 60 | 38 |  |  |
| 881 | TAG(54:3/FA16:0) | 902.8→629.5 | 60 | 38 |  |  |
| 882 | TAG(54:3/FA16:1) | 902.8→631.5 | 60 | 38 |  |  |
| 883 | TAG(54:3/FA18:0) | 902.8→601.5 | 60 | 38 |  |  |
| 884 | TAG(54:3/FA18:1) | 902.8→603.5 | 60 | 38 |  |  |
| 885 | TAG(54:3/FA18:2) | 902.8→605.5 | 60 | 38 |  |  |
| 886 | TAG(54:3/FA18:3) | 902.8→607.5 | 60 | 38 |  |  |
| 887 | TAG(54:3/FA20:1) | 902.8→575.5 | 60 | 38 |  |  |
| 888 | TAG(54:3/FA20:2) | 902.8→577.5 | 60 | 38 |  |  |
| 889 | TAG(54:3/FA20:3) | 902.8→579.5 | 60 | 38 |  |  |
| 890 | TAG(54:4/FA16:0) | 900.8→627.5 | 60 | 38 |  |  |
| 891 | TAG(54:4/FA16:1) | 900.8→629.5 | 60 | 38 |  |  |
| 892 | TAG(54:4/FA18:0) | 900.8→599.5 | 60 | 38 |  |  |
| 893 | TAG(54:4/FA18:1) | 900.8→601.5 | 60 | 38 |  |  |
| 894 | TAG(54:4/FA18:2) | 900.8→603.5 | 60 | 38 |  |  |
| 895 | TAG(54:4/FA18:3) | 900.8→605.5 | 60 | 38 |  |  |
| 896 | TAG(54:4/FA20:1) | 900.8→573.5 | 60 | 38 |  |  |
| 897 | TAG(54:4/FA20:2) | 900.8→575.5 | 60 | 38 |  |  |
| 898 | TAG(54:4/FA20:3) | 900.8→577.5 | 60 | 38 |  |  |
| 899 | TAG(54:4/FA20:4) | 900.8→579.5 | 60 | 38 |  |  |
| 900 | TAG(54:4/FA22:4) | 900.8→551.5 | 60 | 38 |  |  |
| 901 | TAG(54:5/FA16:0) | 898.8→625.5 | 60 | 38 |  |  |
| 902 | TAG(54:5/FA16:1) | 898.8→627.5 | 60 | 38 |  |  |
| 903 | TAG(54:5/FA18:0) | 898.8→597.5 | 60 | 38 |  |  |
| 904 | TAG(54:5/FA18:1) | 898.8→599.5 | 60 | 38 |  |  |
| 905 | TAG(54:5/FA18:2) | 898.8→601.5 | 60 | 38 |  |  |
| 906 | TAG(54:5/FA18:3) | 898.8→603.5 | 60 | 38 |  |  |
| 907 | TAG(54:5/FA20:2) | 898.8→573.5 | 60 | 38 |  |  |
| 908 | TAG(54:5/FA20:3) | 898.8→575.5 | 60 | 38 |  |  |
| 909 | TAG(54:5/FA20:4) | 898.8→577.5 | 60 | 38 |  |  |
| 910 | TAG(54:5/FA20:5) | 898.8→579.5 | 60 | 38 |  |  |
| 911 | TAG(54:5/FA22:4) | 898.8→549.5 | 60 | 38 |  |  |
| 912 | TAG(54:5/FA22:5) | 898.8→551.5 | 60 | 38 |  |  |
| 913 | TAG(54:6/FA16:0) | 896.8→623.5 | 60 | 38 |  |  |
| 914 | TAG(54:6/FA16:1) | 896.8→625.5 | 60 | 38 |  |  |
| 915 | TAG(54:6/FA18:1) | 896.8→597.5 | 60 | 38 |  |  |
| 916 | TAG(54:6/FA18:2) | 896.8→599.5 | 60 | 38 |  |  |
| 917 | TAG(54:6/FA18:3) | 896.8→601.5 | 60 | 38 |  |  |
| 918 | TAG(54:6/FA20:3) | 896.8→573.5 | 60 | 38 |  |  |
| 919 | TAG(54:6/FA20:4) | 896.8→575.5 | 60 | 38 |  |  |
| 920 | TAG(54:6/FA20:5) | 896.8→577.5 | 60 | 38 |  |  |
| 921 | TAG(54:6/FA22:5) | 896.8→549.5 | 60 | 38 |  |  |
| 922 | TAG(54:6/FA22:6) | 896.8→551.5 | 60 | 38 |  |  |
| 923 | TAG(54:7/FA16:1) | 894.8→623.5 | 60 | 38 |  |  |
| 924 | TAG(54:7/FA18:1) | 894.8→595.5 | 60 | 38 |  |  |
| 925 | TAG(54:7/FA18:2) | 894.8→597.5 | 60 | 38 |  |  |
| 926 | TAG(54:7/FA18:3) | 894.8→599.5 | 60 | 38 |  |  |
| 927 | TAG(54:7/FA20:4) | 894.8→573.5 | 60 | 38 |  |  |
| 928 | TAG(54:7/FA20:5) | 894.8→575.5 | 60 | 38 |  |  |
| 929 | TAG(54:7/FA22:5) | 894.8→547.5 | 60 | 38 |  |  |
| 930 | TAG(54:7/FA22:6) | 894.8→549.5 | 60 | 38 |  |  |
| 931 | TAG(54:8/FA18:2) | 892.8→595.5 | 60 | 38 |  |  |
| 932 | TAG(54:8/FA18:3) | 892.8→597.5 | 60 | 38 |  |  |
| 933 | TAG(54:8/FA20:4) | 892.8→571.5 | 60 | 38 |  |  |
| 934 | TAG(54:8/FA20:5) | 892.8→573.5 | 60 | 38 |  |  |
| 935 | TAG(54:8/FA22:6) | 892.8→547.5 | 60 | 38 |  |  |
| 936 | TAG(55:1/FA16:0) | 920.9→647.6 | 60 | 38 |  |  |
| 937 | TAG(55:1/FA18:1) | 920.9→621.6 | 60 | 38 |  |  |
| 938 | TAG(55:2/FA18:1) | 918.8→619.5 | 60 | 38 |  |  |
| 939 | TAG(55:2/FA18:2) | 918.8→621.5 | 60 | 38 |  |  |
| 940 | TAG(55:3/FA18:1) | 916.8→617.5 | 60 | 38 |  |  |
| 941 | TAG(55:3/FA18:2) | 916.8→619.5 | 60 | 38 |  |  |
| 942 | TAG(55:4/FA18:1) | 914.8→615.5 | 60 | 38 |  |  |
| 943 | TAG(55:4/FA18:2) | 914.8→617.5 | 60 | 38 |  |  |
| 944 | TAG(55:5/FA18:2) | 912.8→615.5 | 60 | 38 |  |  |
| 945 | TAG(55:5/FA20:4) | 912.8→591.5 | 60 | 38 |  |  |
| 946 | TAG(55:7/FA22:6) | 908.8→563.5 | 60 | 38 |  |  |
| 947 | TAG(56:10/FA18:2) | 916.7→619.4 | 60 | 38 |  |  |
| 948 | TAG(56:1/FA16:0) | 934.9→661.6 | 60 | 38 |  |  |
| 949 | TAG(56:1/FA18:1) | 934.9→635.6 | 60 | 38 |  |  |
| 950 | TAG(56:2/FA16:0) | 932.9→659.6 | 60 | 38 |  |  |
| 951 | TAG(56:2/FA18:0) | 932.9→631.6 | 60 | 38 |  |  |
| 952 | TAG(56:2/FA20:0) | 932.9→603.6 | 60 | 38 |  |  |
| 953 | TAG(56:2/FA20:1) | 932.9→605.6 | 60 | 38 |  |  |
| 954 | TAG(56:3/FA16:0) | 930.8→657.5 | 60 | 38 |  |  |
| 955 | TAG(56:3/FA18:0) | 930.8→629.5 | 60 | 38 |  |  |
| 956 | TAG(56:3/FA18:1) | 930.8→631.5 | 60 | 38 |  |  |
| 957 | TAG(56:3/FA18:2) | 930.8→633.5 | 60 | 38 |  |  |
| 958 | TAG(56:3/FA20:0) | 930.8→601.5 | 60 | 38 |  |  |
| 959 | TAG(56:3/FA20:1) | 930.8→603.5 | 60 | 38 |  |  |
| 960 | TAG(56:3/FA20:2) | 930.8→605.5 | 60 | 38 |  |  |
| 961 | TAG(56:4/FA16:0) | 928.8→655.5 | 60 | 38 |  |  |
| 962 | TAG(56:4/FA18:0) | 928.8→627.5 | 60 | 38 |  |  |
| 963 | TAG(56:4/FA18:1) | 928.8→629.5 | 60 | 38 |  |  |
| 964 | TAG(56:4/FA18:2) | 928.8→631.5 | 60 | 38 |  |  |
| 965 | TAG(56:4/FA20:1) | 928.8→601.5 | 60 | 38 |  |  |
| 966 | TAG(56:4/FA20:2) | 928.8→603.5 | 60 | 38 |  |  |
| 967 | TAG(56:4/FA20:3) | 928.8→605.5 | 60 | 38 |  |  |
| 968 | TAG(56:4/FA20:4) | 928.8→607.5 | 60 | 38 |  |  |
| 969 | TAG(56:4/FA22:4) | 928.8→579.5 | 60 | 38 |  |  |
| 970 | TAG(56:5/FA16:0) | 926.8→653.5 | 60 | 38 |  |  |
| 971 | TAG(56:5/FA18:0) | 926.8→625.5 | 60 | 38 |  |  |
| 972 | TAG(56:5/FA18:1) | 926.8→627.5 | 60 | 38 |  |  |
| 973 | TAG(56:5/FA18:2) | 926.8→629.5 | 60 | 38 |  |  |
| 974 | TAG(56:5/FA20:1) | 926.8→599.5 | 60 | 38 |  |  |
| 975 | TAG(56:5/FA20:2) | 926.8→601.5 | 60 | 38 |  |  |
| 976 | TAG(56:5/FA20:3) | 926.8→603.5 | 60 | 38 |  |  |
| 977 | TAG(56:5/FA20:4) | 926.8→605.5 | 60 | 38 |  |  |
| 978 | TAG(56:5/FA22:4) | 926.8→577.5 | 60 | 38 |  |  |
| 979 | TAG(56:5/FA22:5) | 926.8→579.5 | 60 | 38 |  |  |
| 980 | TAG(56:6/FA16:0) | 924.8→651.5 | 60 | 38 |  |  |
| 981 | TAG(56:6/FA18:0) | 924.8→623.5 | 60 | 38 |  |  |
| 982 | TAG(56:6/FA18:1) | 924.8→625.5 | 60 | 38 |  |  |
| 983 | TAG(56:6/FA18:2) | 924.8→627.5 | 60 | 38 |  |  |
| 984 | TAG(56:6/FA18:3) | 924.8→629.5 | 60 | 38 |  |  |
| 985 | TAG(56:6/FA20:2) | 924.8→599.5 | 60 | 38 |  |  |
| 986 | TAG(56:6/FA20:3) | 924.8→601.5 | 60 | 38 |  |  |
| 987 | TAG(56:6/FA20:4) | 924.8→603.5 | 60 | 38 |  |  |
| 988 | TAG(56:6/FA20:5) | 924.8→605.5 | 60 | 38 |  |  |
| 989 | TAG(56:6/FA22:4) | 924.8→575.5 | 60 | 38 |  |  |
| 990 | TAG(56:6/FA22:5) | 924.8→577.5 | 60 | 38 |  |  |
| 991 | TAG(56:6/FA22:6) | 924.8→579.5 | 60 | 38 |  |  |
| 992 | TAG(56:7/FA16:0) | 922.8→649.5 | 60 | 38 |  |  |
| 993 | TAG(56:7/FA16:1) | 922.8→651.5 | 60 | 38 |  |  |
| 994 | TAG(56:7/FA18:0) | 922.8→621.5 | 60 | 38 |  |  |
| 995 | TAG(56:7/FA18:1) | 922.8→623.5 | 60 | 38 |  |  |
| 996 | TAG(56:7/FA18:2) | 922.8→625.5 | 60 | 38 |  |  |
| 997 | TAG(56:7/FA18:3) | 922.8→627.5 | 60 | 38 |  |  |
| 998 | TAG(56:7/FA20:3) | 922.8→599.5 | 60 | 38 |  |  |
| 999 | TAG(56:7/FA20:4) | 922.8→601.5 | 60 | 38 |  |  |
| 1000 | TAG(56:7/FA20:5) | 922.8→603.5 | 60 | 38 |  |  |
| 1001 | TAG(56:7/FA22:4) | 922.8→573.5 | 60 | 38 |  |  |
| 1002 | TAG(56:7/FA22:5) | 922.8→575.5 | 60 | 38 |  |  |
| 1003 | TAG(56:7/FA22:6) | 922.8→577.5 | 60 | 38 |  |  |
| 1004 | TAG(56:8/FA16:0) | 920.8→647.5 | 60 | 38 |  |  |
| 1005 | TAG(56:8/FA16:1) | 920.8→649.5 | 60 | 38 |  |  |
| 1006 | TAG(56:8/FA18:1) | 920.8→621.5 | 60 | 38 |  |  |
| 1007 | TAG(56:8/FA18:2) | 920.8→623.5 | 60 | 38 |  |  |
| 1008 | TAG(56:8/FA18:3) | 920.8→625.5 | 60 | 38 |  |  |
| 1009 | TAG(56:8/FA20:4) | 920.8→599.5 | 60 | 38 |  |  |
| 1010 | TAG(56:8/FA20:5) | 920.8→601.5 | 60 | 38 |  |  |
| 1011 | TAG(56:8/FA22:5) | 920.8→573.5 | 60 | 38 |  |  |
| 1012 | TAG(56:8/FA22:6) | 920.8→575.5 | 60 | 38 |  |  |
| 1013 | TAG(56:9/FA18:3) | 918.8→623.5 | 60 | 38 |  |  |
| 1014 | TAG(56:9/FA20:4) | 918.8→597.5 | 60 | 38 |  |  |
| 1015 | TAG(56:9/FA20:5) | 918.8→599.5 | 60 | 38 |  |  |
| 1016 | TAG(56:9/FA22:6) | 918.8→573.5 | 60 | 38 |  |  |
| 1017 | TAG(57:10/FA22:6) | 928.7→583.4 | 60 | 38 |  |  |
| 1018 | TAG(57:2/FA18:1) | 946.9→647.6 | 60 | 38 |  |  |
| 1019 | TAG(57:3/FA18:2) | 944.9→647.6 | 60 | 38 |  |  |
| 1020 | TAG(58:10/FA18:2) | 944.8→647.5 | 60 | 38 |  |  |
| 1021 | TAG(58:10/FA20:4) | 944.8→623.5 | 60 | 38 |  |  |
| 1022 | TAG(58:10/FA20:5) | 944.8→625.5 | 60 | 38 |  |  |
| 1023 | TAG(58:10/FA22:5) | 944.8→597.5 | 60 | 38 |  |  |
| 1024 | TAG(58:10/FA22:6) | 944.8→599.5 | 60 | 38 |  |  |
| 1025 | TAG(58:2/FA18:1) | 960.9→661.6 | 60 | 38 |  |  |
| 1026 | TAG(58:3/FA18:1) | 958.9→659.6 | 60 | 38 |  |  |
| 1027 | TAG(58:5/FA18:1) | 954.9→655.6 | 60 | 38 |  |  |
| 1028 | TAG(58:6/FA16:0) | 952.8→679.5 | 60 | 38 |  |  |
| 1029 | TAG(58:6/FA18:0) | 952.8→651.5 | 60 | 38 |  |  |
| 1030 | TAG(58:6/FA18:1) | 952.8→653.5 | 60 | 38 |  |  |
| 1031 | TAG(58:6/FA20:4) | 952.8→631.5 | 60 | 38 |  |  |
| 1032 | TAG(58:6/FA22:4) | 952.8→603.5 | 60 | 38 |  |  |
| 1033 | TAG(58:6/FA22:5) | 952.8→605.5 | 60 | 38 |  |  |
| 1034 | TAG(58:7/FA20:4) | 950.8→629.5 | 60 | 38 |  |  |
| 1035 | TAG(58:7/FA22:4) | 950.8→601.5 | 60 | 38 |  |  |
| 1036 | TAG(58:7/FA22:5) | 950.8→603.5 | 60 | 38 |  |  |
| 1037 | TAG(58:7/FA22:6) | 950.8→605.5 | 60 | 38 |  |  |
| 1038 | TAG(58:8/FA20:3) | 948.8→625.5 | 60 | 38 |  |  |
| 1039 | TAG(58:8/FA20:4) | 948.8→627.5 | 60 | 38 |  |  |
| 1040 | TAG(58:8/FA22:5) | 948.8→601.5 | 60 | 38 |  |  |
| 1041 | TAG(58:8/FA22:6) | 948.8→603.5 | 60 | 38 |  |  |
| 1042 | TAG(58:9/FA20:4) | 946.8→625.5 | 60 | 38 |  |  |
| 1043 | TAG(58:9/FA22:5) | 946.8→599.5 | 60 | 38 |  |  |
| 1044 | TAG(58:9/FA22:6) | 946.8→601.5 | 60 | 38 |  |  |
| 1045 | DAG(14:0/15:0) | 544.5→285.2 | 60 | 26 |  | + |
| 1046 | DAG(14:0/16:0) | 558.5→285.2 | 60 | 26 |  | + |
| 1047 | DAG(14:0/16:1) | 556.5→285.2 | 60 | 26 |  | + |
| 1048 | DAG(14:0/18:0) | 586.5→285.2 | 60 | 26 |  | + |
| 1049 | DAG(14:0/18:1) | 584.5→285.2 | 60 | 26 |  | + |
| 1050 | DAG(14:0/18:2) | 582.5→285.2 | 60 | 26 |  | + |
| 1051 | DAG(14:0/19:0) | 600.6→285.2 | 60 | 26 |  | + |
| 1052 | DAG(14:0/20:0) | 614.6→285.2 | 60 | 26 |  |  |
| 1053 | DAG(14:0/20:3) | 608.5→285.2 | 60 | 26 |  | + |
| 1054 | DAG(14:0/22:6) | 630.5→285.2 | 60 | 26 |  | + |
| 1055 | DAG(16:0/16:0) | 586.5→313.3 | 60 | 26 | + | + |
| 1056 | DAG(16:0/16:1) | 584.5→313.3 | 60 | 26 |  | + |
| 1057 | DAG(16:0/18:0) | 614.6→313.3 | 60 | 26 | + | + |
| 1058 | DAG(16:0/18:1) | 612.6→313.3 | 60 | 26 | + | + |
| 1059 | DAG(16:0/18:2) | 610.5→313.3 | 60 | 26 | + | + |
| 1060 | DAG(16:0/18:3) | 608.5→313.3 | 60 | 26 |  | + |
| 1061 | DAG(16:0/20:0) | 642.6→313.3 | 60 | 26 |  | + |
| 1062 | DAG(16:0/20:1) | 640.6→313.3 | 60 | 26 |  | + |
| 1063 | DAG(16:0/20:2) | 638.6→313.3 | 60 | 26 |  | + |
| 1064 | DAG(16:0/20:3) | 636.6→313.3 | 60 | 26 |  | + |
| 1065 | DAG(16:0/20:4) | 634.5→313.3 | 60 | 26 |  | + |
| 1066 | DAG(16:0/20:5) | 632.5→313.3 | 60 | 26 |  | + |
| 1067 | DAG(16:0/22:4) | 662.6→313.3 | 60 | 26 |  | + |
| 1068 | DAG(16:0/22:5) | 660.6→313.3 | 60 | 26 |  |  |
| 1069 | DAG(16:0/22:6) | 658.5→313.3 | 60 | 26 |  | + |
| 1070 | DAG(16:1/16:1) | 582.5→311.3 | 60 | 26 |  | + |
| 1071 | DAG(16:1/18:0) | 612.6→311.3 | 60 | 26 |  | + |
| 1072 | DAG(16:1/18:1) | 610.5→311.3 | 60 | 26 | + | + |
| 1073 | DAG(16:1/18:2) | 608.5→311.3 | 60 | 26 |  | + |
| 1074 | DAG(16:1/18:3) | 606.5→311.3 | 60 | 26 |  | + |
| 1075 | DAG(16:1/19:0) | 626.6→311.3 | 60 | 26 |  |  |
| 1076 | DAG(16:1/20:0) | 640.6→311.3 | 60 | 26 |  |  |
| 1077 | DAG(16:1/20:1) | 638.6→311.3 | 60 | 26 |  | + |
| 1078 | DAG(16:1/20:2) | 636.6→311.3 | 60 | 26 |  | + |
| 1079 | DAG(16:1/20:3) | 634.5→311.3 | 60 | 26 |  | + |
| 1080 | DAG(16:1/20:4) | 632.5→311.3 | 60 | 26 |  | + |
| 1081 | DAG(16:1/22:6) | 656.5→311.3 | 60 | 26 |  | + |
| 1082 | DAG(17:0/17:0) | 614.6→327.3 | 60 | 26 |  | + |
| 1083 | DAG(18:0/18:0) | 642.6→341.3 | 60 | 26 | + | + |
| 1084 | DAG(18:0/18:1) | 640.6→341.3 | 60 | 26 |  | + |
| 1085 | DAG(18:0/18:2) | 638.6→341.3 | 60 | 26 |  | + |
| 1086 | DAG(18:0/18:3) | 636.6→341.3 | 60 | 26 |  |  |
| 1087 | DAG(18:0/20:0) | 670.6→341.3 | 60 | 26 |  |  |
| 1088 | DAG(18:0/20:1) | 668.6→341.3 | 60 | 26 |  |  |
| 1089 | DAG(18:0/20:2) | 666.6→341.3 | 60 | 26 |  |  |
| 1090 | DAG(18:0/20:3) | 664.6→341.3 | 60 | 26 |  | + |
| 1091 | DAG(18:0/20:4) | 662.6→341.3 | 60 | 26 |  | + |
| 1092 | DAG(18:0/20:5) | 660.6→341.3 | 60 | 26 |  | + |
| 1093 | DAG(18:0/22:5) | 688.6→341.3 | 60 | 26 |  | + |
| 1094 | DAG(18:0/22:6) | 686.5→341.3 | 60 | 26 | + | + |
| 1095 | DAG(18:1/18:1) | 638.6→339.3 | 60 | 26 | + | + |
| 1096 | DAG(18:1/18:2) | 636.6→339.3 | 60 | 26 | + | + |
| 1097 | DAG(18:1/18:3) | 634.5→339.3 | 60 | 26 |  | + |
| 1098 | DAG(18:1/20:0) | 668.6→339.3 | 60 | 26 |  | + |
| 1099 | DAG(18:1/20:1) | 666.6→339.3 | 60 | 26 |  | + |
| 1100 | DAG(18:1/20:2) | 664.6→339.3 | 60 | 26 |  | + |
| 1101 | DAG(18:1/20:3) | 662.6→339.3 | 60 | 26 | + |  |
| 1102 | DAG(18:1/20:4) | 660.6→339.3 | 60 | 26 |  | + |
| 1103 | DAG(18:1/20:5) | 658.5→339.3 | 60 | 26 |  | + |
| 1104 | DAG(18:1/22:4) | 688.6→339.3 | 60 | 26 | + | + |
| 1105 | DAG(18:1/22:5) | 686.6→339.3 | 60 | 26 | + | + |
| 1106 | DAG(18:1/22:6) | 684.6→339.3 | 60 | 26 | + | + |
| 1107 | DAG(18:2/18:2) | 634.5→337.3 | 60 | 26 | + | + |
| 1108 | DAG(18:2/18:3) | 632.5→337.3 | 60 | 26 |  | + |
| 1109 | DAG(18:2/19:0) | 652.6→337.3 | 60 | 26 |  | + |
| 1110 | DAG(18:2/20:0) | 666.6→337.3 | 60 | 26 |  |  |
| 1111 | DAG(18:2/20:1) | 664.6→337.3 | 60 | 26 |  | + |
| 1112 | DAG(18:2/20:2) | 662.6→337.3 | 60 | 26 |  | + |
| 1113 | DAG(18:2/20:3) | 660.6→337.3 | 60 | 26 |  | + |
| 1114 | DAG(18:2/20:4) | 658.5→337.3 | 60 | 26 | + | + |
| 1115 | DAG(18:2/20:5) | 656.5→337.3 | 60 | 26 |  | + |
| 1116 | DAG(18:2/22:4) | 686.6→337.3 | 60 | 26 |  | + |
| 1117 | DAG(18:2/22:5) | 684.6→337.3 | 60 | 26 |  | + |
| 1118 | DAG(18:2/22:6) | 682.5→337.3 | 60 | 26 | + | + |
| 1119 | MAG 14:0 | 320.4→211.2 | 60 | 18 |  |  |
| 1120 | MAG 16:0 | 348.4→239.2 | 60 | 18 | + | + |
| 1121 | MAG 16:1 | 346.4→237.2 | 60 | 18 |  |  |
| 1122 | MAG 18:0 | 376.4→267.2 | 60 | 18 | + | + |
| 1123 | MAG 18:1 | 374.4→265.2 | 60 | 18 |  | + |
| 1124 | MAG 18:2 | 372.4→263.2 | 60 | 18 |  | + |
| 1125 | MAG 18:3 | 370.4→261.2 | 60 | 18 |  |  |
| 1126 | MAG 20:0 | 404.5→295.3 | 60 | 20 |  |  |
| 1127 | MAG 20:2 | 400.4→291.2 | 60 | 20 |  |  |
| 1128 | MAG 20:3 | 398.4→289.2 | 60 | 20 |  |  |
| 1129 | MAG 20:4 | 396.4→287.2 | 60 | 20 |  |  |
| 1130 | MAG 20:5 | 394.4→285.2 | 60 | 20 |  |  |
| 1131 | MAG 22:0 | 432.5→323.3 | 60 | 20 |  |  |
| 1132 | MAG 22:4 | 424.4→315.2 | 60 | 20 |  |  |
| 1133 | MAG 22:6 | 420.4→311.2 | 60 | 20 |  |  |
| 1134 | MAG 24:0 | 460.5→351.3 | 60 | 20 |  |  |
| 1135 | MAG 17:0 | 362.4→253.2 | 60 | 18 |  |  |
| 1136 | SM(d18:1/14:0) | 675.5→184.1 | 60 | 43 | + | + |
| 1137 | SM(d18:1/16:0) | 703.6→184.1 | 60 | 43 | + | + |
| 1138 | SM(d18:1/18:0) | 731.6→184.1 | 60 | 43 | + | + |
| 1139 | SM(d18:1/18:1) | 729.6→184.1 | 60 | 43 | + | + |
| 1140 | SM(d18:1/20:0) | 759.6→184.1 | 60 | 43 | + | + |
| 1141 | SM(d18:1/20:1) | 757.6→184.1 | 60 | 43 | + | + |
| 1142 | SM(d18:1/22:0) | 787.7→184.1 | 60 | 43 | + | + |
| 1143 | SM(d18:1/22:1) | 785.7→184.1 | 60 | 43 | + | + |
| 1144 | SM(d18:1/24:0) | 815.7→184.1 | 60 | 43 | + | + |
| 1145 | SM(d18:1/24:1) | 813.7→184.1 | 60 | 43 | + | + |
| 1146 | SM(d18:1/26:0) | 843.7→184.1 | 60 | 43 |  |  |
| 1147 | SM(d18:1/26:1) | 841.7→184.1 | 60 | 43 | + | + |
| 1148 | CER(d18:1/14:0) | 510.6→264.4 | 60 | 43 |  | + |
| 1149 | CER(d18:1/16:0) | 538.6→264.4 | 60 | 43 |  | + |
| 1150 | CER(d18:1/18:0) | 566.7→264.4 | 60 | 43 |  | + |
| 1151 | CER(d18:1/20:0) | 594.6→264.4 | 60 | 43 |  | + |
| 1152 | CER(d18:1/22:0) | 622.7→264.4 | 60 | 43 |  | + |
| 1153 | CER(d18:1/22:1) | 620.7→264.4 | 60 | 43 |  | + |
| 1154 | CER(d18:1/24:0) | 650.8→264.4 | 60 | 43 | + | + |
| 1155 | CER(d18:1/24:1) | 648.8→264.4 | 60 | 43 | + | + |
| 1156 | CER(d18:1/26:0) | 678.9→264.4 | 60 | 43 |  | + |
| 1157 | CER(d18:1/26:1) | 676.9→264.4 | 60 | 43 |  | + |
| 1158 | GlcCer(d18:1/16:0) | 700.7→264.4 | 60 | 43 | + | + |
| 1159 | GlcCer(d18:1/20:0) | 756.7→264.4 | 60 | 43 |  |  |
| 1160 | GlcCer(d18:1/22:0) | 784.9→264.4 | 60 | 43 |  |  |
| 1161 | GlcCer(d18:1/22:1) | 782.8→264.4 | 60 | 43 |  |  |
| 1162 | GlcCer(d18:1/24:0) | 812.9→264.4 | 60 | 43 |  |  |
| 1163 | GlcCer(d18:1/24:1) | 810.9→264.4 | 60 | 43 |  |  |
| 1164 | GlcCer(d18:1/26:0) | 840.9→264.4 | 60 | 43 |  |  |
| 1165 | GlcCer(d18:1/26:1) | 838.9→264.4 | 60 | 43 |  |  |
| 1166 | LacCer(d18:1/14:0) | 834.9→264.4 | 60 | 43 |  |  |
| 1167 | LacCer(d18:1/16:0) | 862.9→264.4 | 60 | 43 |  |  |
| 1168 | LacCer(d18:1/18:1) | 888.2→264.4 | 60 | 43 |  |  |
| 1169 | LacCer(d18:1/20:0) | 918.2→264.4 | 60 | 43 |  |  |
| 1170 | LacCer(d18:1/20:1) | 916.2→264.4 | 60 | 43 |  |  |
| 1171 | LacCer(d18:1/22:0) | 946.2→264.4 | 60 | 43 |  |  |
| 1172 | LacCer(d18:1/24:0) | 974.8→264.4 | 60 | 43 |  |  |
| 1173 | LacCer(d18:1/24:1) | 972.9→264.4 | 60 | 43 |  |  |
| 1174 | CE(20:0) | 698.7→369.4 | 60 | 25 |  |  |
| 1175 | CE(14:0) | 614.6→369.4 | 60 | 25 |  | + |
| 1176 | CE(16:0) | 642.6→369.4 | 60 | 25 |  | + |
| 1177 | CE(16:1) | 640.6→369.4 | 60 | 25 | + | + |
| 1178 | CE(18:0) | 670.6→369.4 | 60 | 25 |  | + |
| 1179 | CE(18:1) | 668.6→369.4 | 60 | 25 | + | + |
| 1180 | CE(18:2) | 666.6→369.4 | 60 | 25 | + | + |
| 1181 | CE(18:3) | 664.6→369.4 | 60 | 25 | + | + |
| 1182 | CE(20:2) | 694.6→369.4 | 60 | 25 |  | + |
| 1183 | CE(20:3) | 692.6→369.4 | 60 | 25 | + | + |
| 1184 | CE(20:4) | 690.6→369.4 | 60 | 25 | + | + |
| 1185 | CE(20:5) | 688.6→369.4 | 60 | 25 | + | + |
| 1186 | CE(22:4) | 718.6→369.4 | 60 | 25 |  | + |
| 1187 | CE(22:5) | 716.6→369.4 | 60 | 25 |  | + |
| 1188 | CE(22:6) | 714.6→369.4 | 60 | 25 | + | + |

**Note:** **CE**, cholesterylester; **CER**, ceramide; **DAG**, diglyceride; **FFA**, free fatty acid; **GlcCer**, glucosylceramide; **LacCer**, lactosylceramide; **LPC**, lyso-phosphatidylcholine; **LPE,** lysophosphatidy-lethanolamine; **LPG**, lyso-phosphatidylglycerol; **LPI**, lyso-phosphatidylinositol; **LPS**, lyso-phosphatidylserine; **MAG**, monoglyceride; **PA**, phosphatidic acid; **PC**, phosphatidylcholine; **PE**, phosphatidylethanolamine; **PE**, phosphatidylethanolamine; **PG**, phosphatidyl-glycerol; **PI**, phosphatidyl-inositol; **PS**, phosphatidylserine; **SM**, sphingomyelin; **TAG**, triglyceride.

**Table S6.** Mobile phase elution conditions for PUFAs.

| Time (min) | Mobile Phase B |
| --- | --- |
| 0–10.0 | 48%–75% |
| 10.0–10.1 | 75%–90% |
| 10.1–12.0 | 90% |
| 12.0–12.1 | 90%–48% |
| 12.1–14.0 | 48% |

**Table S7.** MRM transitions of PUFAs on LC-MS/MS.

| **Name** | **MRM (*m/z*)** | **DP** | **EP** | **CE** | **CXP** |
| --- | --- | --- | --- | --- | --- |
| EPA | 301.1→257.0 | -101.99 | -3.73 | -17.32 | -10.96 |
| ARA | 303.0→59.0 | -106.66 | -7.67 | -34.12 | -5.08 |
| 14,15-EET | 319.2→175.1 | -95.01 | -9.63 | -19.19 | -6.15 |
| 11,12-EET | 319.0→167.0 | -78.21 | -9.71 | -20.11 | -5.05 |
| 5-HETE | 319.1→301.3 | -71.92 | -12.86 | -15.3 | -7.01 |
| 15-HETE | 319.0→300.9 | -86.15 | -14.33 | -18.06 | -15.09 |
| 11(S)-HETE | 319.0→167.1 | -91.18 | -3.03 | -22.02 | -6.09 |
| 8,9-EET | 319.2→257.1 | -91.8 | -7.17 | -18.69 | -13.63 |
| DHA | 327.1→59.0 | -142.88 | -6.21 | -43.24 | -5.19 |
| PGE_2_ | 351.1→315.2 | -65.99 | -6.8 | -17.68 | -10.06 |
| PGD_2_ | 351.0→315.1 | -62.01 | -4.9 | -15.75 | -13.01 |
| 14(15)-DiHET | 337.1→207.0 | -75.03 | -7.05 | -25.79 | -8.02 |
| 11(12)-DiHET | 337.0→167.0 | -77.97 | -10.67 | -25.83 | -6.36 |

**Table S8.** Compounds identified from MLN, serum and liver using UHPLC-Q-Exactive Orbitrap MS.

| No. | Compounds | Category | RT/min | Molecular formula | Ion mode | Measured mass /Da | Calculated mass /Da | Error /ppm | Fragment ions | Serum-HFHFD area/10e5 | Serum-MLN area/10e5 | Liver-HFHFD area/10e5 | Liver-MLN area/10e5 | Source |
| --- | --- | --- | --- | --- | --- | --- | --- | --- | --- | --- | --- | --- | --- | --- |
| 1 | Betaine* | Others | 0.88 | C_5_H_11_NO_2_ | [M+H]+ | 118.08650 | 118.08626 | 2.073 | 139.98788;72.08154;59.07383;58.00600;55.05516 | 16282.22 | 18143.91 | 54485.59 | 85689.63 | ALL |
| 2 | Gluconic acid | Organic acid | 0.96 | C_6_H_12_O_7_ | [M-H]- | 195.04977 | 195.04993 | -0.816 | 177.03967;159.02855;141.01799;129.01791;87.00717;80.96357;75.00715;59.01225 | 185.80 | 116.70 | 8967.78 | 11893.56 | ALL |
| 3 | glc-glc-glc | Others | 0.97 | C_18_H_32_O_16_ | [M+Na]+ | 527.15845 | 527.15826 | 0.368 | 365.10550;347.09491;288.68216;221.44142;203.05264;185.04163;157.22758;82.53469 | 0.00 | 21.83 | 1364.73 | 82.71 | ALL |
| 4 | Sucrose | Others | 0.97 | C_12_H_22_O_11_ | [M-H]- | 341.10925 | 341.10784 | 4.14 | 198.32870;179.05515;161.04445;143.03365;131.03383;119.03352;113.02292;101.02286;89.02280;71.01226;59.01230 | 0.00 | 0.00 | 0.00 | 113.28 | ALL |
| 5 | Shikimic acid | Organic acid | 1.05 | C_7_H_10_O_5_ | [M-H]- | 173.04446 | 173.04555 | 0.059 | 155.03383;137.02298;111.04367;93.03302;73.02789 | 0.00 | 0.00 | 0.00 | 0.00 | DC;LJF;LF |
| 6 | Erythrose-glucoside | Others | 1.87 | C_10_H_18_O_8_ | [M-H]- | 265.09290 | 265.09179 | 4.172 | 179.05495;143.03355;131.03343;119.03361;113.02297;101.02293;89.02293;71.01230;59.01228 | 0.33 | 5.14 | 0.85 | 12.81 | ABR;SR;LJF;LF |
| 7 | Sucrose acetate | Others | 1.87 | C_14_H_24_O_12_ | [M-H]- | 383.11914 | 383.11840 | 1.925 | 341.10849;323.09897;179.05515;161.04468;113.02283;101.02310;89.02293;71.01221;59.01228 | 0.00 | 0.00 | 0.00 | 0.00 | ABR;SR;LJF |
| 8 | Adenosine | Others | 1.95 | C_10_H_13_N_5_O_4_ | [M+H]+ | 268.10352 | 268.10403 | -1.904 | 224.33896;178.65515;150.40453;136.06183;115.03965;85.02908;57.03441 | 96.77 | 100.14 | 8976.51 | 3423.68 | ALL |
| 9 | Gallic acid* | Organic acid | 2.04 | C_7_H_6_O_5_ | [M-H]- | 169.01309 | 169.01315 | -0.354 | 162.38057;128.02469;125.022298;115.95132;125.02298 | 0.00 | 0.00 | 2.84 | 70.31 | SR;DC;LJF;LF |
| 10 | Dihydro-hydroxy-caffeoylquinic acid | Organic acid | 2.15 | C_16_H_20_O_10_ | [M-H]- | 371.09827 | 371.09727 | 2.686 | 353.08783;191.05521;179.03374;173.04434;135.04373 | 0.00 | 0.00 | 0.00 | 0.00 | LJF;LF |
| 11 | Gastrodin* | Others | 2.95 | C_13_H_18_O_7_ | [M+HCOO-]- | 331.10376 | 331.10236 | 4.233 | 285.09875;237.63257;;161.04469;146.81441;123.04386 | 0.00 | 0.72 | 0.00 | 0.00 | LJF;LF |
| 12 | Aucubin* | Iridoids | 4.16 | C_15_H_22_O_9_ | [M+HCOO-]- | 391.12485 | 391.12349 | 3.483 | 345.11859;331.45206;250.69888;183.06514;165.05467; | 0.00 | 0.00 | 0.00 | 0.00 | SR |
| 13 | 3,4-Dihydroxybenzoic acid* | Organic acid | 4.34 | C_7_H_6_O_4_ | [M-H]- | 153.01811 | 153.01824 | -0.818 | 131.144196;120.95836;109.02795;103.88098;81.06294 | 12.15 | 30.75 | 18.75 | 152.27 | ALL |
| 14 | Gentisic acid 5-O-β-D-glucopyranoside | Organic acid | 4.66 | C_13_H_16_O_9_ | [M-H]- | 315.07227 | 315.07106 | 3.845 | 170.59512;153.01804;109.02789;91.24710;58.31604 | 0.00 | 33.35 | 0.00 | 25.45 | ALL |
| 15 | Leonuriside A | Others | 5.42 | C_14_H_20_O_9_ | [M-H]- | 331.10376 | 331.10346 | 4.233 | 299.07794;209.04445;168.04170;153.01811;113.02294 | 0.00 | 2.83 | 0.00 | 0.00 | ALL |
| 16 | Pantothenic acid | Organic acid | 5.84 | C_9_H_17_O_5_N | [M+H]+ | 220.11771 | 220.11795 | -1.086 | 202.10754;192.06535;184.09676;166.08624;142.08643;124.07597;116.03461;103.07592;98.02422;90.05559;85.06554 | 3891.82 | 2934.27 | 17464.60 | 24979.07 | ALL |
| 17 | 4-Hydroxybenzoic acid | Organic acid | 6.57 | C_7_H_6_O_3_ | [M-H]- | 137.02290 | 137.02332 | -3.069 | 109.02771;93.03291;81.03280;58.47685 | 0.00 | 0.00 | 0.00 | 0.00 | ABR;SR;LJF |
| 18 | Neochlorogenic acid* | Organic acid | 6.61 | C_16_H_18_O_9_ | [M-H]- | 353.08731 | 353.08671 | 1.704 | 241.83678;191.05525;179.03392;146.93723;135.04393; | 0.00 | 60.83 | 0.00 | 471.67 | LJF;LF |
| 19 | Harpagide* | Iridoids | 7.28 | C_15_H_24_O_10_ | [M+HCOO-]- | 409.13528 | 409.13405 | 3.000 | 363.13019;253.61256;244.40417;201.07648;183.06573;179.05510;166.48149;157.05016;139.03868;125.02361;119.03404 | 0.00 | 33.99 | 0.00 | 98.72 | SR |
| 20 | Ajugol* | Iridoids | 7.58 | C_15_H_24_O_9_ | [M+HCOO-]- | 393.14029 | 393.13914 | 2.930 | 347.13461;322.10327;185.08119;167.07013;149.05989 | 0.00 | 0.22 | 0.00 | 0.00 | SR |
| 21 | Syringic acid-glucoside | Organic acid | 7.76 | C_15_H_20_O_10_ | [M-H]- | 359.09708 | 359.09727 | -0.538 | 271.09720;242.05768;197.04460;182.02109;153.05452;138.03088;123.00724 | 0.00 | 2.94 | 0.00 | 13.95 | ALL |
| 22 | Esculin | Others | 8.05 | C_15_H_16_O_9_ | [M-H]- | 339.07230 | 339.07106 | 3.662 | 208.88730;192.82446;177.01808;133.02817;80.56728 | 0.00 | 0.00 | 0.00 | 2.87 | LF |
| 23 | 4-Hydroxy-benzaldehyde* | Others | 8.87 | C_7_H_6_O_2_ | [M-H]- | 121.02824 | 121.02841 | -1.371 | 108.02023;93.03319;76.96864;61.91845;55.85628 | 0.00 | 0.00 | 0.00 | 0.00 | ALL |
| 24 | Dihydrocaffeic acid | Organic acid | 9.18 | C_9_H_10_O_4_ | [M-H]- | 181.04951 | 181.04954 | -0.140 | 137.05939;135.04362;121.02802;119.04847;109.02808;92.91832;71.01237;59.01226 | 0.00 | 0.00 | 0.00 | 0.00 | DC;LJF;LF |
| 25 | Caffeoylquinic acid-glc | Organic acid | 9.54 | C_22_H_28_O_14_ | [M-H]- | 515.14148 | 515.13953 | 3.782 | 374.51697;323.07669;290.10297;191.05542;161.02280 | 0.00 | 0.00 | 0.00 | 0.00 | LJF;LF |
| 26 | Lonijaposide M | Iridoids | 9.56 | C_25_H_31_NO_11_ | [M+H]+ | 522.19714 | 522.19699 | 0.293 | 404.46555;360.14508;318.82230;290.10226;273.10043;246.11227;204.06506;179.09396;160.07558;87.04461 | 0.00 | 2.12 | 0.00 | 10.42 | LJF |
| 27 | Shashenoside I | Phenylpropanoids | 9.58 | C_22_H_32_O_13_ | [M+HCOO-]- | 549.18240 | 549.18140 | 1.827 | 503.17691;471.09421;234.83035;179.05516;161.04378 | 0.00 | 0.00 | 0.00 | 0.00 | DC;LJF |
| 28 | Chlorogenic acid* | Organic acid | 9.68 | C_16_H_18_O_9_ | [M-H]- | 353.08676 | 353.08671 | 0.146 | 272.54733;191.055508;102.68142 | 2.75 | 54.12 | 10.37 | 361.05 | LJF;LF |
| 29 | Baihuaqianhuoside | Phenylpropanoids | 9.69 | C_16_H_22_O_8_ | [M+HCOO-]- | 387.13135 | 387.12857 | 7.173 | 341.11005;318.93808;179.07021;164.04678;71.-1182 | 0.00 | 0.00 | 0.00 | 6.01 | DC;LJF |
| 30 | Caffeic acid* | Organic acid | 9.75 | C_9_H_8_O_4_ | [M-H]- | 179.03386 | 179.03389 | -0.141 | 135.04373;122.89320;107.04871;90.92284;74.65268 | 0.00 | 0.00 | 0.00 | 675.81 | SR;DC;LJF;LF |
| 31 | 4-Hydroxycinnamic acid glucoside | Organic acid | 10.54 | C_15_H_18_O_8_ | [M-H]- | 325.09317 | 325.09179 | 4.233 | 203.48367;179.68307;163.03880;119.04877;72.06716 | 0.00 | 16.33 | 0.00 | 6.80 | ALL |
| 32 | Cryptochlorogenic acid* | Organic acid | 10.65 | C_16_H_18_O_9_ | [M-H]- | 353.08777 | 353.08671 | 3.006 | 306.76962;247.38544;210.69199;191.05511;179.03404;173.04443;135.04370;117.46970;93.03281;82.58212 | 2.32 | 87.30 | 1.80 | 392.70 | LJF;LF |
| 33 | Loganic acid* | Iridoids | 10.83 | C_16_H_24_O_10_ | [M-H]- | 375.12906 | 375.12857 | 1.297 | 299.30563;281.72446;213.07648;169.08629;157.59608;113.02283;89.02293;69.03297 | 0.00 | 79.73 | 0.00 | 0.00 | LJF;LF |
| 34 | Darendoside A** | Others | 10.92 | C_19_H_28_O_11_ | [M+Na]+ | 455.15268 | 455.15238 | 0.653 | 388.06265;295.35495;205.90685;187.86061;116.43134; | 0.00 | 0.00 | 0.00 | 0.76 | SR;DC;LJF;LF |
| 35 | 2-Methoxyphenyl-β-d-glucopyranoside** | Others | 10.99 | C_13_H_18_O_7_ | [M+Na]+ | 309.09418 | 309.09447 | -0.951 | 276.87308;218.86821;208.88365;149.89738;72.93784 | 0.20 | 23.98 | 0.00 | 7.23 | DC;LJF;LF |
| 36 | 4-(β-d-Glucopyranosyloxy)-3,5-dimethoxyphenylpropanone** | Phenylpropanoids | 11.21 | C_17_H_24_O_9_ | [M+Na]+ | 395.13116 | 395.13125 | -0.236 | 277.82678;232.07019;185.04137;123.99703;81.87772 | 0.00 | 0.00 | 0.00 | 3.67 | SR;DC;LJF;LF |
| 37 | Dehydro-secologanin | Iridoids | 11.30 | C_17_H_22_O_10_ | [M-H]- | 385.11346 | 385.11292 | 1.394 | 330.91324;265.06998;223.06073;208.03700;179.07013;164.04677;161.05952;149.02315;119.03274;89.02264 | 0.00 | 0.00 | 0.00 | 0.00 | ABR;SR;LJF |
| 38 | Geniposidic acid-diglucoside | Iridoids | 11.55 | C_28_H_42_O_21_ | [M-H]- | 713.21411 | 713.21348 | 0.877 | 341.10886;319.59583;269.14404;179.05550;139.00214;119.03337;101.02259;89.02293;71.01207;59.01216 | 0.00 | 0.00 | 0.00 | 0.32 | LJF |
| 39 | 1,3-Dicaffeoylquinic acid* | Organic acid | 12.25 | C_25_H_24_O_12_ | [M-H]- | 515.11957 | 515.11840 | 2.266 | 366.40842;353.08804;335.07782;261.09760;228.34540;191.05537;179.03419;173.04485;161.02348;135.04404;111.04384 | 0.00 | 0.00 | 0.00 | 11.61 | LJF;LF |
| 40 | Secologanic acid | Iridoids | 12.28 | C_16_H_22_O_10_ | [M-H]- | 373.11365 | 373.11292 | 1.948 | 193.04965;167.07024;149.05946;123.04362;119.03329;101.02290;97.02794;89.02280;71.01227;59.01225 | 0.00 | 42.93 | 0.00 | 96.16 | LJF;LF |
| 41 | Secologanoside* | Iridoids | 12.45 | C_16_H_22_O_11_ | [M-H]- | 389.10895 | 389.10784 | 2.858 | 345.11880;209.04448;165.5450;121.06444;89.02283 | 0.75 | 260.91 | 1.67 | 690.23 | LJF;LF |
| 42 | p-Hydroxycinnamic acid | Organic acid | 12.96 | C_9_H_8_O_3_ | [M-H]- | 163.03891 | 163.04007 | -0.372 | 119.04878;93.03299;56.821003 | 4.52 | 84.17 | 9.97 | 26.62 | SR;DC;LJF;LF |
| 43 | Geniposide* | Iridoids | 13.61 | C_17_H_24_O_10_ | [M+HCOO-]- | 433.13510 | 433.13405 | 2.418 | 387.13037;379.12219;295.54059;278.53006;225.07626;171.18869;162.07379;123.04364;101.02288;85.02010 | 0.00 | 3.68 | 0.00 | 8.87 | LJF;LF |
| 44 | 5-Hydroxymethyl-2-furaldehyde | Others | 13.84 | C_6_H_6_O_3_ | [M+H]+ | 127.03928 | 127.03897 | 2.435 | 110.02393;109.02879;99.04457;97.02896;85.02907;81.03422;71.04995;55.01876 | 0.00 | 0.00 | 0.00 | 110.18 | DC;LJF;LF |
| 45 | Secologanoside A | Iridoids | 13.86 | C_21_H_32_O_14_ | [M-H]- | 507.17163 | 507.17083 | 1.574 | 357.11972;327.10840;283.11899;195.06563;177.05492;149.04417;125.02289;101.02268;89.02272;71.01218;59.01224 | 0.00 | 7.95 | 0.00 | 38.95 | LJF;LF |
| 46 | Sweroside* | Iridoids | 13.86 | C_16_H_22_O_9_ | [M+HCOO-]- | 403.12439 | 403.12349 | 2.238 | 357.11880;195.06583;179.05492;125.02295;119.03365 | 0.00 | 140.31 | 0.00 | 229.99 | SR;LJF;LF |
| 47 | Longifloroside B | Lignans | 14.08 | C_27_H_36_O_13_ | [M+HCOO-]- | 613.21442 | 613.21270 | 2.810 | 567.20941;512.93585;405.15521;357.13394;225.07631;195.06546;145.79199;116.20657;56.20686 | 0.00 | 0.00 | 0.00 | 0.00 | DC |
| 48 | Loganin* | Iridoids | 14.91 | C_17_H_26_O_10_ | [M+HCOO-]- | 435.15109 | 435.14970 | 3.188 | 338.32324;315.91068;295.78174;227.09213;217.00078;141.21744;127.03879;115.98772;101.02261;100.10978;57.13235 | 0.00 | 24.18 | 0.00 | 30.84 | LJF;LF |
| 49 | Hygrine | Lignans | 14.93 | C_8_H_15_NO | [M+HCOO-]- | 186.11263 | 186.11247 | 0.860 | 142.12215;125.09580;117.81173;99.92410;97.06413;82.28408;54.63834 | 0.00 | 0.00 | 283.70 | 206.68 | SR;DC;LJF;LF |
| 50 | Ferulic acid* | Organic acid | 14.96 | C_10_H_10_O_4_ | [M+H]+ | 195.06375 | 195.06519 | -7.358 | 186.05499;177.05461;163.03876;149.05986;145.02843;135.04404;117.03362;89.03906;70.06579;58.06603 | 0.00 | 0.00 | 0.00 | 44.19 | ABR;SR;DC |
| 51 | Apigenin-8-C-(2''-O-β-D-glucopyranosyl)-α-L-arabinopyranoside** | Flavone | 15.83 | C_26_H_28_O_14_ | [M-H]- | 563.14075 | 563.14063 | 2.163 | 473.10983;443.09930;383.07739;353.06683;325.07297;297.07645;151.32442;91.80633 | 0.00 | 0.00 | 0.00 | 1.84 | DC |
| 52 | (+)-Lyoniresinol 9'-O-glucoside* | Lignans | 15.96 | C_28_H_38_O_13_ | [M-H]- | 581.22455 | 581.22287 | 2.895 | 419.17303;404.14893;389.12463;371.11478;344.82861;233.08217;174.89592;153.05527;138.03078;101.02312;89.02280;71.01224;59.01227 | 0.00 | 0.00 | 0.00 | 0.35 | SR;LJF;LF |
| 53 | Citrusin B** | Lignans | 16.12 | C_27_H_36_O_13_ | [M+Na]+ | 591.20508 | 591.20481 | 0.453 | 429.15344;365.12048;351.10492;232.07076;175.03653 | 0.00 | 0.00 | 0.00 | 0.40 | DC |
| 54 | Phenylethyl-glc-xyl | Others | 16.21 | C_19_H_28_O_10_ | [M+HCOO-]- | 461.16626 | 461.16535 | 1.967 | 415.16095;191.05553;179.05498;149.04424;131.03357;113.02287;101.02283;89.02283;71.01221;59.01225 | 0.00 | 2.70 | 0.00 | 15.55 | SR;LJF;LF |
| 55 | Vicenin-1 | Flavone | 16.30 | C_26_H_28_O_14_ | [M-H]- | 563.14063 | 563.14063 | 1.950 | 473.10889;443,09833;383.07730;365.06613;353.06671;325.07245;297.07660;216.90010117.03304 | 0.58 | 3.43 | 0.00 | 4.24 | DC |
| 56 | Salicylic acid* | Organic acid | 16.39 | C_7_H_6_O_3_ | [M-H]- | 137.02318 | 137.02332 | -1.026 | 109.61245;94.03644;93.03314;84.50281;72.69837;65.03808 | 40.89 | 872.35 | 33.63 | 130.14 | ALL |
| 57 | Apigenin 6-C-α-L-arabinopyranosyl-8-C-β-D-xylopyranoside | Flavone | 16.52 | C_25_H_26_O_13_ | [M-H]- | 533.12994 | 533.13006 | 1.825 | 473.10693;443.09927;383.07716;353.06665;325.07162;237.97357;132.17905 | 0.00 | 0.00 | 0.00 | 0.22 | DC |
| 58 | Foliachinenoside C** | Lignans | 16.59 | C_28_H_38_O_14_ | [M+Na]+ | 621.21539 | 621.21538 | 0.021 | 459.16162;395.13129;232.07054;205.04730;185.04234 | 0.00 | 0.00 | 0.00 | 0.00 | DC;LJF |
| 59 | Schaftoside* | Flavone | 16.67 | C_26_H_28_O_14_ | [M+H]+ | 565.15588 | 565.15518 | 1.235 | 547.14583;529.13586;457.10986;415.10300;337.07037;295.05829;218.62770;171.57892;90.36653 | 0.00 | 1.35 | 0.00 | 4.68 | DC |
| 60 | Secoxyloganin* | Iridoids | 16.75 | C_17_H_24_O_11_ | [M-H]- | 403.12460 | 403.12349 | 2.759 | 371.09836;223.06059;191.03416;179.05545;165.05452;139.00220;121.02798;101.02275;89.02270;71.012;59.01223 | 0.00 | 327.50 | 0.29 | 881.25 | LJF;LF |
| 61 | Rubrosterone* | Sterones | 17.13 | C_19_H_26_O_5_ | [M+H]+ | 335.18524 | 335.18530 | -0.180 | 317.17453;299.16425;281.15326;257.15421;239.14316;187.11189;171.08017;131.08565 | 0.00 | 0.00 | 0.00 | 0.00 | ABR |
| 62 | (-)-Medioresinol di-O-β-D-glucopyranoside | Lignans | 17.27 | C_33_H_44_O_17_ | [M+HCOO-]- | 757.25562 | 757.25496 | 0.878 | 636.02179;603.09271;549.19720;531.18642;433.14920;369.13345;179.05516;89.02298 | 0.00 | 0.00 | 0.00 | 0.16 | DC |
| 63 | Isoquercetin | Flavone | 17.33 | C_21_H_20_O_12_ | [M-H]- | 463.08804 | 463.08820 | 2.025 | 343.04727;301.03513;220.55437;210.85855;175.00262;151.00252;140.32289;121.02814;60.30630 | 0.00 | 0.00 | 0.00 | 0.75 | LJF;LF |
| 64 | Syringaresinol 4', 4''- di- O-*β*- D- glucopyranoside | Lignans | 17.59 | C_34_H_46_O_18_ | [M+HCOO-]- | 787.26715 | 787.26552 | 2.070 | 705.82361;579.20813;417.15549;181.04962;91.80566 | 0.00 | 0.34 | 0.00 | 2.38 | DC |
| 65 | (-)-Syringaresinol-4''-O-*β*-D-monoglucopyranoside | Lignans | 17.59 | C_28_H_36_O_13_ | [M-H]- | 579.20361 | 579.20832 | -6.228 | 417.15591;387.10797;181.04965;166.02599 | 0.00 | 0.00 | 0.00 | 0.00 | DC |
| 66 | Achyranthesterone A | Sterones | 17.84 | C_27_H_44_O_8_ | [M+HCOO-]- | 541.30170 | 541.30072 | 1.803 | 495.39617;403.24875;319.19131;175.09642;157.08484;145.08545;139.07492;113.05915;99.04284;87.04293;85.02789;83.04861;73.02719 | 0.00 | 0.46 | 0.00 | 13.11 | ABR |
| 67 | Dihydrodehydrodiconiferyl alcohol 4-O-β-D-glucopyranoside | Others | 17.98 | C_26_H_34_O_11_ | [M+HCOO-]- | 567.20801 | 567.20722 | 1.397 | 521.19604;374.90991;359.15018;349.81744;341.13882;329.13974;317.88812;176.81839;137.89977 | 0.00 | 0.00 | 0.00 | 3.71 | LF |
| 68 | Corymbosin/5-hydroxy-7,3',4',5'-tetramethoxyflavone | Flavone | 18.02 | C_19_H_18_O_7_ | [M+HCOO-]- | 403.10150 | 403.10236 | -2.130 | 371.09680;244.23090;223.06117;209.04543;193.04967;178.02589;165.05479;150.03065;134.03577;121.02821;101.02261;89.02290;59.01212 | 0.00 | 0.00 | 0.00 | 0.00 | DC;LJF |
| 69 | Isoschaftoside* or Vicenin 3* | Flavone | 18.25 | C_26_H_28_O_14_ | [M+H]+ | 565.15564 | 565.15518 | 0.811 | 547.14038;481.11401;469.11267;397.08929;379.08102;337.07025;325.07028;295.05902;222.28273;130.06612;65.23441 | 0.00 | 0.00 | 0.00 | 0.95 | DC |
| 70 | Manglieside E** | Lignans | 18.42 | C_28_H_38_O_13_ | [M-H]- | 581.22595 | 581.22397 | 5.303 | 419.17172;389.16116;208.07367;91.81672 | 0.00 | 0.00 | 0.00 | 0.00 | DC |
| 71 | L-Phenylalaninosecologanin | Iridoids | 18.47 | C_26_H_35_NO_11_ | [M+H]+ | 538.22827 | 538.22829 | -0.032 | 376.17480;351.95193;341.13776;256.13263;228.10196;211.09657;141.05458;85.02894 | 0.00 | 0.00 | 0.00 | 28.93 | LJF;LF |
| 72 | (1′S,6′R)-8′-hydroxyabscisic acid-β-d-glucoside** | Others | 18.49 | C_21_H_30_O_10_ | [M-H]- | 441.17767 | 441.17552 | 4.865 | 415.53534;330.13321;223.34196;113.02280;89.02304;59.01242 | 0.00 | 0.00 | 0.00 | 0.14 | DC;LJF;LF |
| 73 | Cynaroside* | Flavone | 18.54 | C_21_H_20_O_11_ | [M+H]+ | 449.10736 | 449.10784 | -1.064 | 310.21762;299.61813;287.05505;135.53621;91.98315 | 0.00 | 0.00 | 0.00 | 27.72 | LJF;LF |
| 74 | (E)-Aldosecologanin | Iridoids | 18.60 | C_34_H_46_O_19_ | [M+HCOO-]- | 803.26166 | 803.26043 | 1.526 | 757.25372;595.20215;525.15948;423.06613;267.14413;289.11588;179.05522;119.03336;101.02278;89.02293;71.01218 | 0.00 | 0.00 | 0.00 | 0.21 | LJF |
| 75 | Luteolin-glc-rha-xyl | Flavone | 18.60 | C_32_H_38_O_19_ | [M-H]- | 725.19336 | 725.19236 | 1.386 | 538.64569;335.79929;285.03958;284.03156;191.05501 | 0.00 | 0.00 | 0.00 | 0.17 | LJF |
| 76 | Isochlorogenic acid B | Organic acid | 18.73 | C_25_H_24_O_12_ | [M-H]- | 515.11993 | 515.11840 | 2.965 | 353.08820;335.07767;203.03423;191.05533;179.03415;173.04466;161.02350;155.03397;135.04395;111.04364;93.03396 | 6.44 | 15.19 | 5.74 | 285.67 | LJF;LF |
| 77 | 6-O-Methylaucubin | Iridoids | 18.78 | C_16_H_24_O_9_ | [M-H]- | 359.13467 | 359.13366 | 2.816 | 230.78349;197.08096;153.09091;135.08009;109.06436;89.02271;71.01231;59.01238 | 0.00 | 25.07 | 0.00 | 20.21 | LJF;LF |
| 78 | Rutin* | Flavone | 18.95 | C_27_H_30_O_16_ | [M-H]- | 609.14697 | 609.14501 | 3.216 | 535.73035;449.83334;300.02786;271.02438;178.99780 | 0.66 | 0.31 | 1.03 | 2.49 | LJF;LF |
| 79 | Apigenin-6,8-di-C-β-d-xylopyranoside** | Flavone | 19.03 | C_25_H_26_O_13_ | [M-H]- | 533.13037 | 533.13006 | 2.631 | 443.10117;383.07916;353.06781;285.73132;215.05264;96.19996 | 0.00 | 0.75 | 0.00 | 7.02 | DC |
| 80 | Acteoside* | Phenylpropanoids | 19.17 | C_29_H_36_O_15_ | [M-H]- | 623.19849 | 623.19705 | 2.316 | 461.16571;206.87395;179.03316;161.02315;145.53267;135.04390;113.02273;85.02817;71.01230 | 0.00 | 0.00 | 0.00 | 0.00 | SR |
| 81 | Isoviolanthin** | Flavone | 19.50 | C_27_H_30_O_14_ | [M-H]- | 577.15674 | 577.15628 | 2.700 | 457.11734;383.07791;353.06693;297.07776;117.03304 | 0.00 | 0.65 | 0.00 | 2.27 | DC |
| 82 | Dehydrodiconiferyl alcohol-4- *β*-D-glucoside | Flavone | 19.64 | C_26_H_32_O_11_ | [M+HCOO-]- | 565.19604 | 565.19157 | 7.913 | 519.18640;501.17587;403.13892;388.11578;339.11819;254.75406;181.0494;119.03344;89.02282 | 0.00 | 0.00 | 0.00 | 0.00 | DC |
| 83 | Coniferyl ρ-coumarate** | Phenylpropanoids | 19.70 | C_19_H_22_O_5_ | [M+Na]+ | 353.13824 | 353.13595 | 6.499 | 321.11212;289.08575;261.09091;233.09622;191.07028;167.07033;131.04912;107.04964;79.05479 | 0.00 | 0.00 | 0.00 | 0.00 | DC |
| 84 | (7S,8R,8'R)-4,4',9-trihydroxy-3,3',5,5'-tetramethoxy-7,9'-epoxylignan-7'-one-4'-O-*β*-d-glucopyranoside** | Lignans | 19.72 | C_28_H_36_O_14_ | [M-H]- | 595.20282 | 595.20323 | 1.156 | 549.19861;531.18805;369.13504;179.05525;119.03381;89.02298 | 0.00 | 0.00 | 0.00 | 0.00 | DC |
| 85 | Lonicerin* | Flavone | 19.80 | C_27_H_30_O_15_ | [M-H]- | 593.15179 | 593.15119 | 2.855 | 447.09525;343.19504;285.04041;284.03262 | 0.00 | 2.56 | 0.00 | 7.99 | LJF;LF |
| 86 | Isoviolanthin isomer | Flavone | 19.93 | C_27_H_30_O_14_ | [M-H]- | 577.15594 | 577.15628 | 1.314 | 457.11554;383.07748;353.06653;168.21115 | 0.00 | 0.00 | 0.00 | 0.00 | DC |
| 87 | Lirioresionol** | Lignans | 20.11 | C_22_H_26_O_8_ | [M-H]- | 417.15591 | 417.15549 | 3.634 | 387.10977;181.04976;166.02611;137.02327;67.82862 | 0.00 | 0.00 | 0.00 | 0.00 | DC |
| 88 | (+)-Syringaresinol-*β*-D-glucoside* | Lignans | 20.11 | C_28_H_36_O_13_ | [M-H]- | 579.21155 | 580.21504 | 7.480 | 417.15604;402.13135;387.10812;181.04980;166.02617;152.04640;119.91298;64.03983 | 0.00 | 0.00 | 0.00 | 2.16 | ALL |
| 89 | Sinapyl ρ-coumarate** | Phenylpropanoids | 20.13 | C_20_H_24_O_6_ | [M+Na]+ | 383.14893 | 383.14651 | 6.317 | 351.12286;337.10602;291.10147;276.07898;229.08604 | 0.00 | 0.00 | 0.00 | 0.00 | DC |
| 90 | Acanthoside B** | Lignans | 20.13 | C_28_H_36_O_13_ | [M+Na]+ | 603.20685 | 603.20481 | 3.378 | 441.05070;331.09940;185.04176 | 0.00 | 0.00 | 0.00 | 0.00 | ALL |
| 91 | Threoninosecologanin | Iridoids | 20.56 | C_20_H_27_NO_11_ | [M+H]+ | 458.16553 | 458.16569 | -0.343 | 296.11276;278.10193;226.07100;208.06046;180.06544;164.03406;130.04999;124.03942;85.02924 | 0.00 | 7.72 | 0.00 | 31.83 | LJF;LF |
| 92 | Hexyl-xyl-glc | Others | 20.62 | C_17_H_32_O_10_ | [M+HCOO-]- | 395.19193 | 441.19665 | 3.869 | 395.19193;263.14932;131.03336;101.02289 | 0.00 | 0.00 | 0.00 | 0.00 | SR;LJF;LF |
| 93 | Apigenin-7-O-D-glucoside* | Flavone | 20.65 | C_21_H_20_O_10_ | [M-H]- | 431.09872 | 431.09837 | 3.356 | 381.42337;375.47385;268.03806;252.09727;190.38748;172.89259;125.45736;87.87401;77.59495;69.41560;65.50101 | 1.08 | 6.97 | 0.00 | 9.80 | LJF;LF |
| 94 | Xylostosidine | Others | 20.82 | C_18_H_25_NSO_8_ | [M+H]+ | 416.13889 | 416.13736 | 3.667 | 254.08446;236.07529;184.04272;149.05986;124.03958 | 0.00 | 1.88 | 0.00 | 11.33 | LJF;LF |
| 95 | Coumaryl-caffeoylquinic acid | Organic acid | 20.94 | C_25_H_24_O_11_ | [M-H]- | 499.12448 | 499.12349 | 1.988 | 431.09863;353.08829;337.09323;319.08145;191.05518;173.04420;163.03885;119.04842 | 0.00 | 0.00 | 0.00 | 2.65 | LJF;LF |
| 96 | Shashenoside I** | Phenylpropanoids | 21.12 | C_22_H_32_O_13_ | [M-H]- | 503.17795 | 503.17702 | 4.040 | 457.16995;293.08783;209.15964;149.04453;125.02295;89.02298;71.01238 | 0.64 | 0.34 | 0.00 | 8.67 | DC;LJF;LF |
| 97 | Dendromoniliside E | Others | 21.12 | C_28_H_38_O_14_ | [M+HCOO-]- | 643.22510 | 643.22326 | 2.858 | 597.21930;435.16507;273.11310;181.29228;68.84463 | 0.00 | 0.00 | 0.00 | 0.00 | DC |
| 98 | Isochlorogenic acid A* | Organic acid | 21.30 | C_25_H_24_O_12_ | [M-H]- | 515.12042 | 515.11840 | 3.917 | 353.08817;287.67682;205.64626;191.05531;179.03397;173.04449;155.03444;135.04396;117.62712;87.59566;55.39125 | 21.91 | 32.27 | 18.71 | 178.71 | LJF;LF |
| 99 | Azelaic acid | Organic acid | 21.52 | C_9_H_16_O_4_ | [M-H]- | 187.09657 | 187.09649 | 0.452 | 169.08471;143.10657;125.09873;123.08028;99.92411;97.06437;91.98110;57.03297 | 669.17 | 841.93 | 1180.88 | 1241.16 | ABR;SR;DC |
| 100 | Polypodine B | Sterones | 21.55 | C_27_H_44_O_8_ | [M+HCOO-]- | 541.30273 | 541.30072 | 3.706 | 495.29648;417.28354;335.18842;245.15471;159.10149 | 0.00 | 0.72 | 0.00 | 18.57 | ABR;DC |
| 101 | 20-Hydroxyecdysone* | Sterones | 21.59 | C_27_H_44_O_7_ | [M+HCOO-]- | 525.30646 | 525.30581 | 1.239 | 479.30161;319.19095;301.18317;224.48500;184.17638;159.10139;83.04843 | 0.00 | 11.52 | 0.00 | 254.12 | ABR |
| 102 | Rhoifolin | Flavone | 21.64 | C_27_H_30_O_14_ | [M+H]+ | 579.17047 | 579.17083 | -0.625 | 433.11075;338.753336;271.05991;85.02898 | 0.00 | 0.00 | 0.00 | 12.98 | LJF;LF |
| 103 | (25R)-Inokosterone* | Sterones | 21.73 | C_27_H_44_O_7_ | [M+HCOO-]- | 525.30682 | 525.30581 | 1.924 | 479.30298;468.29034;345.52133;319.19229;159.10168 | 0.00 | 2.01 | 0.00 | 63.34 | ABR |
| 104 | Chrysoeriol-7-O-neohesperidoside | Flavone | 22.22 | C_28_H_32_O_15_ | [M+H]+ | 609.17993 | 609.18140 | -1.467 | 527.93597;463.12454;301.07053;286.04721;258.05249;85.02901 | 0.00 | 0.00 | 0.00 | 13.92 | DC;LJF;LF |
| 105 | (25S)-Inokosterone* | Sterones | 22.35 | C_27_H_44_O_7_ | [M+HCOO-]- | 525.30634 | 525.30581 | 1.010 | 479.30136;155.50378;142.21011;81.84521;72.96316 | 0.00 | 2.66 | 0.00 | 76.13 | ABR |
| 106 | Tricin 7-O-neohesperidoside | Flavone | 22.47 | C_29_H_34_O_16_ | [M+H]+ | 639.19269 | 639.19196 | 1.140 | 379.18390;331.08105;315.04910;270.05203;112.55640 | 0.00 | 0.00 | 0.00 | 0.37 | LJF;LF |
| 107 | (Z)-Aldosecologanin | Iridoids | 22.67 | C_34_H_46_O_19_ | [M+HCOO-]- | 803.26184 | 803.26043 | 1.750 | 757.25580;595.20166;525.16016;493.17148;273.95932;179.05464;119.03366;101.02286;89.02281;71.01214;59.01230 | 0.00 | 0.00 | 0.00 | 0.51 | LJF;LF |
| 108 | Hydroxydecanoic acid-glc-xyl | Organic acid | 23.03 | C_21_H_38_O_12_ | [M-H]- | 481.22803 | 481.22795 | 0.160 | 435.25839;349.18591;266.62891;187.13289;101.02254 | 0.00 | 0.00 | 0.00 | 4.64 | LJF;LF |
| 109 | Angoroside C* | Phenylpropanoids | 23.14 | C_36_H_48_O_19_ | [M-H]- | 783.27112 | 783.27061 | 0.657 | 607.22430;589.20605;533.93878;267.83588;193.04930;175.03885;160.01491;134.03606;89.02283 | 0.00 | 18.66 | 0.00 | 236.15 | SR |
| 110 | (-)-Syringaresinol* | Lignans | 23.14 | C_22_H_26_O_8_ | [M+Na]+ | 441.15085 | 441.15199 | -2.582 | 366.08484;199.29308;93.77714;57.10016;53.49615;51.64207 | 0.00 | 1.28 | 0.00 | 30.35 | DC |
| 111 | Dihydro-hydroxy-caffeoyl-caffeoylquinic acid ethyl ester | Organic acid | 23.17 | C_27_H_30_O_13_ | [M-H]- | 561.15985 | 561.16027 | -0.744 | 529.77203;515.11841;399.12915;369.10010;353.08820;223.47485;191.05533;179.03369;173.04453;161.02287;135.04344;93.03284;83.36228 | 0.00 | 0.00 | 0.00 | 0.22 | LJF;LF |
| 112 | Ethyl Caffeate | Organic acid | 23.61 | C_11_H_12_O_4_ | [M-H]- | 207.06531 | 207.06519 | 0.602 | 189.07639;162.92413;113.02280;97.02797;85.02796;79.95580;59.01218 | 0.00 | 0.00 | 0.00 | 4.59 | LJF;LF |
| 113 | Quercetin* | Flavone | 24.23 | C_15_H_10_O_7_ | [M-H]- | 301.03513 | 301.03428 | 2.827 | 273.03973;268.94556;178.99757;151.00237;121.02786;107.01222;88.53249;63.51027;58.28025 | 0.00 | 0.00 | 0.00 | 0.00 | LF |
| 114 | Martynoside | Phenylpropanoids | 24.43 | C_31_H_40_O_15_ | [M-H]- | 651.23053 | 651.22835 | 3.352 | 475.18259;323.80307;193.04988;175.03909;160.01550 | 0.00 | 0.00 | 0.00 | 0.15 | SR |
| 115 | Luteolin* | Flavone | 25.12 | C_15_H_10_O_6_ | [M-H]- | 285.04080 | 285.03936 | 5.036 | 267.02875;241.04965;217.04979;199.03929;175.03944;151.00258;133.02802;125.08750;86.61503;81.11841;70.63555 | 0.00 | 5.52 | 0.00 | 20.31 | LF |
| 116 | Isomartynoside | Phenylpropanoids | 25.62 | C_31_H_40_O_15_ | [M-H]- | 651.22943 | 651.22835 | 1.663 | 559.07172;475.17737;384.29715;270.72885;193.04962;175.03893;160.01500;113.02299 | 0.00 | 0.00 | 0.00 | 1.69 | SR |
| 117 | 8-O-courmaroylharpagide | Iridoids | 25.70 | C_24_H_30_O_12_ | [M-H]- | 509.16534 | 509.16535 | -0.025 | 463.21738;183.06508;163.03888;145.02817;119.04878; | 0.00 | 0.00 | 0.00 | 7.10 | SR |
| 118 | Cistanoside D | Phenylpropanoids | 25.92 | C_31_H_40_O_15_ | [M-H]- | 651.22943 | 651.22835 | 1.663 | 599.07172;475.17737;455.82147;384.29715;270.72885;193.04962;175.03893;160.01500;134.03564;113.02299;89.02266 | 0.00 | 0.00 | 0.00 | 1.69 | SR |
| 119 | L-Phenylalaninosecologanin B | Iridoids | 27.99 | C_25_H_31_NO_10_ | [M+H]+ | 506.20190 | 506.20207 | -0.341 | 467.38983;396.86429;344.14914;326.13831;298.14355;280.13284;274.10709;228.10179;179.07030;132.08081 | 0.00 | 0.62 | 0.00 | 33.14 | LJF |
| 120 | Isorhamnetin* | Flavone | 28.12 | C_16_H_12_O_7_ | [M-H]- | 315.05145 | 315.04993 | 4.827 | 300.02795;219.84558;158.86084;151.00252;144.65231 | 0.00 | 0.00 | 0.00 | 0.00 | LF |
| 121 | Harpagoside* | Iridoids | 29.30 | C_24_H_30_O_11_ | [M+HCOO-]- | 539.17505 | 539.17592 | -1.609 | 493.17093;345.11874;165.05447;147.04370;119.03358 | 0.00 | 2.28 | 0.00 | 107.42 | SR |
| 122 | Cinnamic acid* | Organic acid | 29.32 | C_9_H_8_O_2_ | [M+H]+ | 149.05968 | 149.05971 | -0.175 | 149.02338;131.04916;121.06502;107.04948;95.04958;93.07041;91.05478;84.96032;81.03413;79.05487;65.03936 | 0.00 | 0.00 | 0.00 | 61.46 | SR |
| 123 | 8-O-methoxy-cinnamoylharpagide | Iridoids | 30.03 | C_25_H_32_O_12_ | [M+HCOO-]- | 569.18726 | 569.18648 | 1.367 | 523.18219;422.56564;345.11945;283.40613;183.06474;179.05544;177.05475;165.05476;119.04896;89.02295;71.01215 | 0.00 | 0.00 | 0.00 | 2.17 | SR |
| 124 | 3,4'-Dyhydroxy4,5-dimethylbenzyl** | Bibenzyls | 31.10 | C_16_H_18_O_4_ | [M+H]+ | 275.12820 | 275.12779 | 1.506 | 167.07036;135.08049;121.06509;105.07031;79.05481 | 0.00 | 0.00 | 0.00 | 0.00 | DC |
| 125 | Amentoflavone* | Flavone | 33.36 | C_30_H_18_O_10_ | [M-H]- | 537.08337 | 537.08162 | 3.253 | 443.04263;417.06180;399.05161;375.05157;331.06122;307.06381;283.80472;203.03510;159.04407;137.08911;117.03339;93.53972;85.60241;73.75997;69.48489 | 0.00 | 65.48 | 0.00 | 0.00 | LF |
| 126 | Tinosporaic acid A | Phenylpropanoids | 33.54 | C_16_H_20_O_6_ | [M-H]- | 307.11691 | 307.11871 | -2.295 | 239.12892;195.13834;154.06256;102.95538 | 0.12 | 0.67 | 0.00 | 0.00 | ABR;SR;LJF;LF |
| 127 | Macranthoidin B* | Triterpenoid saponins | 34.12 | C_65_H_106_O_32_ | [M-H]- | 1397.65906 | 1397.65835 | 0.510 | 1073.55322;969.18982;749.45105;603.38910;471.34857 | 0.00 | 8.37 | 0.00 | 16.59 | LJF;LF |
| 128 | Macranthoside B | Triterpenoid saponins | 34.14 | C_53_H_86_O_22_ | [M+H]+ | 1075.56799 | 1075.56835 | -0.360 | 1006.50580;716.66559;619.41974;540.37445;455.35245;437.34116;309.11768;203.17990;145.04961;129.05465;85.02906 | 0.00 | 1.74 | 0.00 | 43.76 | LJF;LF |
| 129 | Macranthoidin A | Triterpenoid saponins | 34.69 | C_59_H_96_O_27_ | [M+HCOO-]- | 1281.61035 | 1281.61100 | -0.653 | 1235.60693;1069.51685;911.50269;749.45349;603.39063;471.34982;423.32605;149.07695 | 0.00 | 1.51 | 0.00 | 38.26 | LJF;LF |
| 130 | Dipsacoside B* | Triterpenoid saponins | 35.11 | C_53_H_86_O_22_ | [M+HCOO-]- | 1119.55005 | 1119.55818 | -7.751 | 1073.55298;749.44824;585.37903;471.34729;323.09790 | 0.00 | 9.15 | 0.37 | 274.74 | LJF;LF |
| 131 | Aurantiamide acetate* | Others | 35.59 | C_27_H_28_N_2_O_4_ | [M+H]+ | 445.21527 | 445.21218 | 6.932 | 385.19269;252.10176;224.10736;194.11800;177.09135;134.09677;117.07048;105.03413;91.05493 | 3.84 | 3.04 | 6.52 | 12.35 | LJF |
| 132 | Ginsenoside Ro | Triterpenoid saponins | 38.15 | C_48_H_76_O_19_ | [M-H]- | 955.49097 | 955.48971 | 1.323 | 793.42604;652.93909;544.25513;259.18738;101.02286 | 0.00 | 5.86 | 0.00 | 257.90 | ABR |
| 133 | Chikusetsusaponin IV | Triterpenoid saponins | 38.98 | C_47_H_74_O_18_ | [M-H]- | 925.47601 | 925.47914 | -3.384 | 793.44208;763.42822;701.42810;569.38806;455.35370;352.83273;192.40178;130.99731 | 0.00 | 0.00 | 0.00 | 15.29 | ABR |
| 134 | 3-O-β-D-Glc-(1-2)-[[2-carboxy-1-(carboxymethl)-2-hydroxyethyl]-(1-3)]-β-D-GluA-28-O-β-D-Glc oleanolicacid | Triterpenoid saponins | 38.98 | C_47_H_72_O_19_ | [M-H]- | 939.45935 | 939.45841 | 1.005 | 893.36481;847.36603;777.44525;732.49451;569.38855;551.37457;516.98633;455.35394;403.56821;278.75281;184.95215;146.96436;139.00201;113.02293;99.00732;91.98858 | 0.00 | 0.00 | 0.00 | 0.00 | ABR |
| 135 | Achyranthoside E | Triterpenoid saponins | 39.23 | C_46_H_70_O_19_ | [M-H]- | 925.44971 | 925.44276 | 7.514 | 793.43852;763.41986;701.42639;613.38267;613.37897;569.38428;497.37076;455.35391;130.99715;113.02282;103.00212;85.02783;76.00719;72.99153 | 0.00 | 0.00 | 0.00 | 0.23 | ABR |
| 136 | Chikusetsusaponin iva* | Triterpenoid saponins | 39.49 | C_42_H_66_O_14_ | [M-H]- | 793.43933 | 793.43688 | 2.447 | 732.00146;631.38397;569.38403;503.54239;497.36603;455.35275;360.21872;157.01338;113.02304;99.00731;85.02800;75.00727;71.01233;57.03306 | 0.63 | 0.68 | 0.00 | 331.86 | ABR |
| 137 | Hederagenin-28-O-β-D-glucopyranosylester | Triterpenoid saponins | 40.58 | C_36_H_58_O_9_ | [M+HCOO-]- | 679.40692 | 679.40519 | 2.548 | 633.39587;471.34811;357.43277;207.05020;129.73296 | 0.00 | 0.00 | 0.00 | 4.35 | LJF;LF |
| 138 | Zingibroside R1 | Triterpenoid saponins | 41.15 | C_42_H_66_O_14_ | [M-H]- | 793.43848 | 793.43688 | 2.013 | 613.37756;569.38440;551.36853;455.35427;351.06741;284.17206;208.30905;113.02302;101.02287;71.01226 | 81.33 | 30.27 | 0.00 | 18.62 | ABR |
| 139 | 28-Desglucosyl-chikusetsusaponinIV | Triterpenoid saponins | 41.29 | C_41_H_64_O_13_ | [M-H]- | 763.42731 | 763.42632 | 1.299 | 732.23169;613.37598;569.38538;455.35080;326.37790;130.94339;71.01233 | 0.00 | 0.00 | 0.00 | 0.00 | ABR |
| 140 | Oleanolic acid 3-O-β-D-glucopyranoside | Triterpenoid saponins | 41.37 | C_36_H_58_O_8_ | [M+HCOO-]- | 663.41156 | 663.41027 | 1.938 | 617.40674;575.96576;455.35321;361.22232;267.9521;207.04996;101.02236;59.01176 | 0.00 | 0.00 | 0.00 | 0.00 | ABR |
| 141 | 28-Desglucosyl-chikusetsusaponinIVa | Triterpenoid saponins | 41.40 | C_36_H_56_O_9_ | [M-H]- | 631.38513 | 631.38406 | 1.695 | 555.37024;509.36163;455.35382;232.23070;157.01299;113.022296;85.02799;75.00722 | 0.00 | 0.00 | 0.00 | 177.21 | ABR |
| 142 | Oleanolic acid* | Triterpenoid saponins | 42.21 | C_30_H_48_O_3_ | [M-H]- | 455.35373 | 455.35197 | 3.861 | 385.13327;371.06903;360.28629;180.40370;95.66034;91.98051;84.78593;57.23545 | 0.00 | 6.54 | 0.00 | 4.03 | ABR;LJF;LF |
| 143 | 3-O-Methylgigantol** | Bibenzyls | 43.03 | C_17_H_20_O_4_ | [M+Na]+ | 311.12616 | 311.12538 | 2.506 | 225.04326;157.72334;142.93871;97.10167;69.07059 | 0.00 | 0.00 | 0.00 | 0.00 | DC |

* Identified by comparison with standards. * *Identified by comparison with the pre-isolated compounds of the subject group.

**Figure S1.** HPLC chromatogram of BS, RCs, and MLN. 1. Harpagide；2. Chlorogenic acid; 3. *β*-ecdysterone; 4. Cymaroside; 5. Isochlorogenic A; 6. Harpagoside; 7. Macranthoidin B; 8. Dipsacoside B.

**Figure S2. Total ion chromatograms (TICs) of individual herbal ingredients in MLN. A1** Positive ion mode of *Achyranthis bidentatae* radix. **A2** Negative ion mode of *Achyranthis bidentata*e radix. **B1** Positive ion mode of *Scrophulariae* radix. **B2** Negative ion mode of *Scrophulariae* radix. **C1** Positive ion mode of *Dendrobium* caulis. **C2** Negative ion mode of *Dendrobium* caulis. **D1** Positive ion mode of *Lonicerae japonicae* flos. **D2** Negative ion mode of *Lonicerae japonicae* flos. **E1** Positive ion mode of *Lonicerae* flos. **E2** Negative ion mode of *Lonicerae* flos.

**Figure S3. Changes in feed intake, body weight, and blood glucose levels in rats under the HFHFD model.** A Feed intake of rats (*n* = 6). B Body weight of rats throughout the experiment (*n* = 6). C Time course of blood glucose levels analysis after oral glucose administration (*n* = 6).

**Figure S4. Screening of the lipid-lowering activity of compounds in Mailuoning Oral Liquid (MLN).** A Information on the compounds. B Representative BODIPY fluorescence images. C Quantitative analysis of the mean fluorescence intensity (MFI); ART, atorvastatin, was used as the positive control. Data are presented as the mean ± SEM (*n* = 3). ***p* < 0.01, ****p* < 0.001 vs BSA group; #*p* < 0.05, ##*p* < 0.01, ###*p* < 0.001 vs NEFA group.

**Figure S5. Visualization of the predicted binding pockets of C41 and C136 with COX-2.** A Predicted binding pocket of C41 with COX-2. B Predicted binding pocket of C136 with COX-2.

**Figure S6.** Uncropped images of the original western blots in figure 6 (*n*=3).
